# Characterization of biological variation of peripheral blood immune cytome in an Indian cohort

**DOI:** 10.1038/s41598-019-51294-7

**Published:** 2019-10-14

**Authors:** Parna Kanodia, Gurvinder Kaur, Poonam Coshic, Kabita Chatterjee, Teresa Neeman, Anna George, Satyajit Rath, Vineeta Bal, Savit B. Prabhu

**Affiliations:** 1 National Institute of Immunology, Aruna Asaf Ali Marg, New Delhi, India; 2 Laboratory Oncology, Dr. B. R. Ambedkar Institute Rotary Cancer Hospital, All India Institute of Medical Sciences, New Delhi, India; 3 Department of Transfusion Medicine, All India Institute of Medical Sciences, New Delhi, India; 4 Department of Statistics, Australian National University, ACT, Canberra, Australia; 5 Wellcome Trust Research Laboratory, Christian Medical College, Vellore, Tamil Nadu India

**Keywords:** Functional clustering, Immunology

## Abstract

Immune parameters show characteristic normal baseline levels and variance in the population. We characterised the degree of inter-individual and within-individual variation over one-year time period in 33 immune cell subsets by flow cytometry in peripheral blood mononuclear cells from 43 healthy young adult volunteers. Our analysis revealed that immune subsets that showed low variability between individuals also showed low short-term fluctuations within-individuals, as well as concordance in siblings. However, when baseline levels and degree of fluctuation were considered together, individuals failed to cluster into discreet groups. Together, the data reveal complex inter-relationships between immune subsets in individuals, and provide insights into the observed heterogeneity between individuals and between multiple immune subsets.

## Introduction

Individuals in a population show considerable heterogeneity in immune responses^1,2^. Multiple factors determine such differences including genetic diversity^3,4^, geographical differences^5^ or environmental factors^6,7^. Immune responses are coordinated by the complex interplay of multiple immune cell subsets^8^. Mechanisms of regulation of steady-state immune cellular levels in individuals would also be expected to reflect in the degree of variation they show in the population. We can conceive population-level immune system heterogeneity at two levels: (1) the differences that are observed across immune subsets^9^ and (2) the differences observed across individuals^10^.

Deep immune phenotyping of large human populations have been conducted previously and have provided considerable insight into genetic and environmental factors regulating immune cytome^3,6,7,11,12^. However, some of these studies differ in their conclusions with regard to the relative importance of genetics/environment in determining immune parameters. For example, one recent genome wide association study (GWAS) in 1000 unrelated individuals reported innate immune parameters to be more significantly determined by genetic influences compared to adaptive immune parameters^13^. Another twin-based study suggested adaptive immune parameters to be more under genetic control^14^. Methodological differences could account for some of these differences. But, even in such studies, the proportion of variance that can be conclusively attributed to genetic or environmental factors is low for many subsets^13^ indicating under-appreciated complexity in their regulation.

Although most of the previously quoted studies have been conducted on caucasian ethnicities, differences in population genetic structure and environmental exposures could give rise to differences across geographical areas. We therefore extended these previous approaches and attempted to understand the structure and pattern of cellular immune parameters in a cohort of young adults from National Capital Region (NCR), India. We describe differences between immune cell subsets and their correlates. We attempt to understand the determinants of immune heterogeneity between individuals by quantifying the degree of variation that is attributable to variation within individuals. We also characterise immune cell subsets that are apparently driven by short term environmental exposures and differentiate them from subsets that show stability within-individuals, subsets that show lower overall variance in the population and those that show concordance in siblings.

## Results

### Quantification of inter-individual and within-individual variability of immune subsets

We first characterised the distribution of immune cell populations in a group of 43 young adult volunteers. Average age of the volunteers was 25.8 (SD = 1.8) and male female ratio was 25:18. The markers used to define subsets and parent gates for each subset is shown in Supplementary Dataset Table S1. Ranges of values of 35 immune subsets (expressed as frequency of the parent gate and as absolute counts) is shown in Supplementary Fig. S1 and Supplementary Dataset Table S2. The immune cell subsets did not differ between males and females (summary statistics, p-values and adjusted p-values for each subset presented in Supplementary Dataset Table S3). There was no seasonal variability observed for any of the immune subsets (Supplementary Fig. S2).

We next quantified the degree of inter-individual and within-individual variation along with variation in technical replicates in each immune cell subset (Supplementary Dataset Table S4). When technical variation was calculated, iNKT cells showed very high variance and formed an outlier by a wide margin. Hence, we removed iNKT cells from further analysis. Since total leukocyte counts (TLC) could not be assayed together, the absolute counts for the immune subsets that is derived from TLC will be confounded by assay-to-assay variability over a one-year time-period; hence, the variance of absolute count data is not interpreted in this study, although the raw data is provided as Supplementary Dataset Table S5 (anonymised raw data).

**Figure 1:**
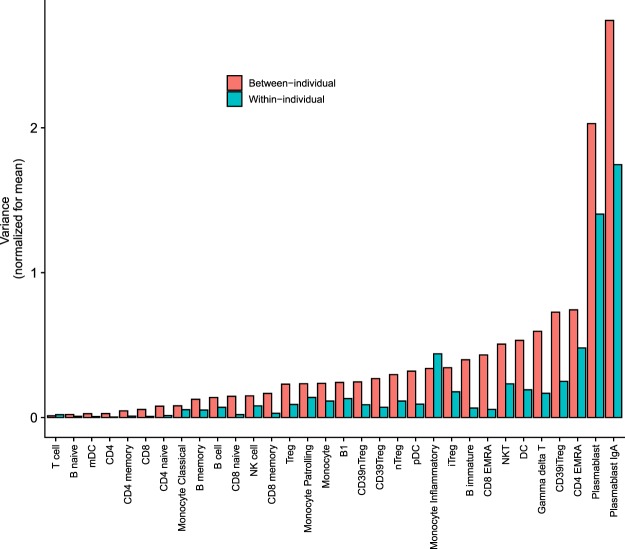
Descriptive characterization of variance of mean-normalized frequencies of immune subsets between-individuals and within-individuals. Each subset is described as % of parent gate as defined in Supplementary Dataset Table S1. Raw data for Fig. 1 is tabulated in Supplementary Dataset Table S4. Adjustment for technical variability is shown in Supplementary Fig. S3 and Supplementary Dataset Table S4. Statistical comparisons are presented in Fig. 2, Supplementary Fig. S5 and Supplementary Dataset Table S6.

Subsets differed with regard to their degree of variability between individuals (Fig. 1 and Supplementary Dataset Table S4). Subsets like plasmablasts, T effector memory CD45RA positive (TEMRA) cells, gamma delta T cells and inflammatory monocytes showed higher variance compared to subsets like classical monocytes, CD4 T cells and memory T and B cells (Fig. 1 and Supplementary Dataset Table S4). Within-individual variances were lower in magnitude compared to between-individual variances for most subsets (Fig. 1), but showed strong association with each other in a linear regression model (Adjusted R-squared = 0.94, t-value = 22.8, p < 2e-16), even after adjusting for the degree of technical variability as a confounding factor (Adjusted R-squared = 0.94, t-value = 23.1, p < 2e-16) (Supplementary Fig. S3 and Supplementary Dataset Table S4). Relatively higher technical variability was seen in monocytes, immature B cells, pDC and iTregs, but there was no association of technical variability with within-individual or between-individual variances (Supplementary Fig. S3). Variability across subsets was also not related to differences in population size of the different subsets in technical replicates (Supplementary Fig. S3).

Together, these results suggested that cell subsets that show high variability between individuals also exhibit high degree of fluctuation within-individuals, possibility due to sensitivity of these subsets to short term environmental fluctuations.

### Baseline levels of most immune subsets are set differently in each individual

By quantifying within-individual variation in individuals and comparing it to inter-individual variation, we attempted to test two competing models (cartoon, shown in Supplementary Fig. S4). In model 1, each individual shows significant within-individual variation that is equal to inter-individual variation, such that outliers in the population are constituted by outliers in within-individual fluctuation. In model 2, each individual’s cellular phenotype is ‘set’ at a baseline, around which there is a small degree of fluctuation, such that outliers in the population are constituted by individuals with extreme baseline levels (Supplementary Fig. S4).

Supplementary Fig. S4 shows illustrative plots of raw data of the total variation between individuals (left panel) and the within-individual variation in each individual (right panel) for each immune cell subset (frequency expressed as % of parent gate indicated in Supplementary Dataset Table S1). When visualised this way, we observed two layers of heterogeneity - (1) the heterogeneity in steady-state ‘baseline’ levels (some individuals showing higher baseline levels compared to others) and (2) the heterogeneity in the degree of within-individual fluctuation between individuals (some individuals showing higher within-individual temporal fluctuation compared to others).

**Figure 2:**
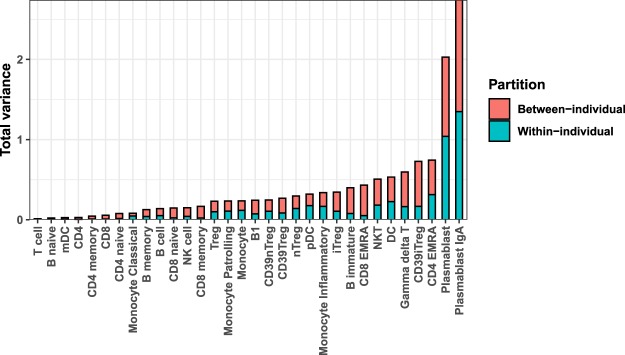
Variance partitioning of frequencies of each immune subset into within-individual component and between-individual component. Each subset is described as % of parent gate as defined in Supplementary Dataset Table S1.

This raised two questions to be quantified for each subset: (1) What component of total inter-individual variation is contributed by intra-individual variation? and (2) Is the average intra-individual variation different from (less than) inter-individual variation? We formally quantified the contribution of within-individual variation to the total variation between individuals using a variance components model (linear mixed effects model) (Fig. 2). For most subsets, there was a low contribution of within-individual variation to total variance implying that the baseline levels are set differently. This suggested that data for most of the subsets favoured the second hypothetical model described before (Supplementary Fig. S4) and the contribution of within-individual fluctuation to the phenotypic variability observed among individuals was low.

To test whether average intra-individual variation is different from (less than) inter-individual variation, we used a hypothesis testing approach using permutation test. We compared the within-individual-variance with between-individual-variance in randomly sampled groups of individuals (Supplementary Fig. S5 and Supplementary Dataset Table S6). Subsets that showed a higher between-individual-variance compared to within-individual-variance included memory B and T cell subsets (Supplementary Dataset Table S6). Subsets that are possibly environmentally determined (TEMRA subsets, plasmablasts) showed no differences in variances between-individuals versus within-individuals (Supplementary Dataset Table S6).

### Quantification of heterogeneity between individuals: correlation between baseline levels and degree of variation within-individuals identify environmentally responsive subsets

**Figure 3:**
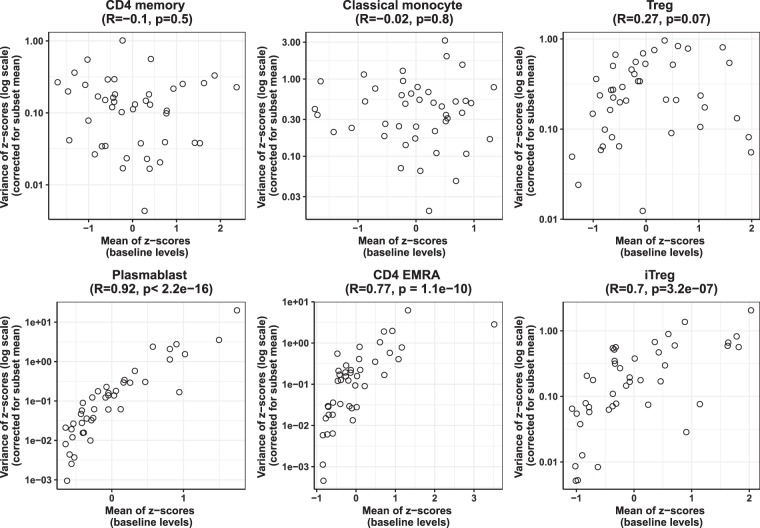
Correlation between baseline levels (mean intra-individual z-score transformed values) and degree of fluctuation (variance of intra-individual z-score transformed values) of immune parameter within an individual. Spearman’s correlation coefficient and p-values as indicated.

The analysis so far suggested that for most subsets, individuals showed heterogeneity in their baseline steady-state levels. One possibility remained that differences in baseline levels in these individuals could be because of environmental exposure related heterogeneity at least in some subsets. If that is the case, we predicted that individuals with high fluctuation may show higher steady-state levels (ie. the upward fluctuation in response to environmental exposures would push the average values up) and in such environmentally-driven subsets, individuals with extremes of steady-state levels of immune phenotype would show extreme fluctuations. We tested this by plotting, for each immune parameter, the relationship between ‘baseline’ values of the phenotype (ie. mean) and the degree of fluctuation (ie. variance normalised to the mean) within each individual (Fig. 3, Supplementary Dataset Table S7). In order to ensure comparability between the axes for different subsets, values for each subset were first expressed as z-scores (ie. expressed in terms of how far the values of each individual deviate from mean for each subset in the cohort) (details in methods section). Z-score transformation also enabled correction of variances of different subsets for the differences in their population sizes, ensuring comparability across subsets.

This analysis (mean-variance plots) showed that for TEMRA, plasmablast, gamma delta T and inflammatory monocytes, there was a highly significant positive correlation between intra-individual mean and intra-individual variance (corrected for mean) (Fig. 3, Supplementary Dataset Table S7), suggesting that these immune subsets could be the environmental-responsive subsets. For subsets such as memory CD4, memory CD8 and memory B cells, there was no correlation between within-individual mean and variance (Fig. 3, Supplementary Dataset Table S7), suggesting that the baseline levels of these subsets could be ‘set’ by factors not related to environmental fluctuations.

### Immune subsets that show higher inter-individual variance also show higher degree of correlation between baseline levels and degree of fluctuation within individuals

**Figure 4:**
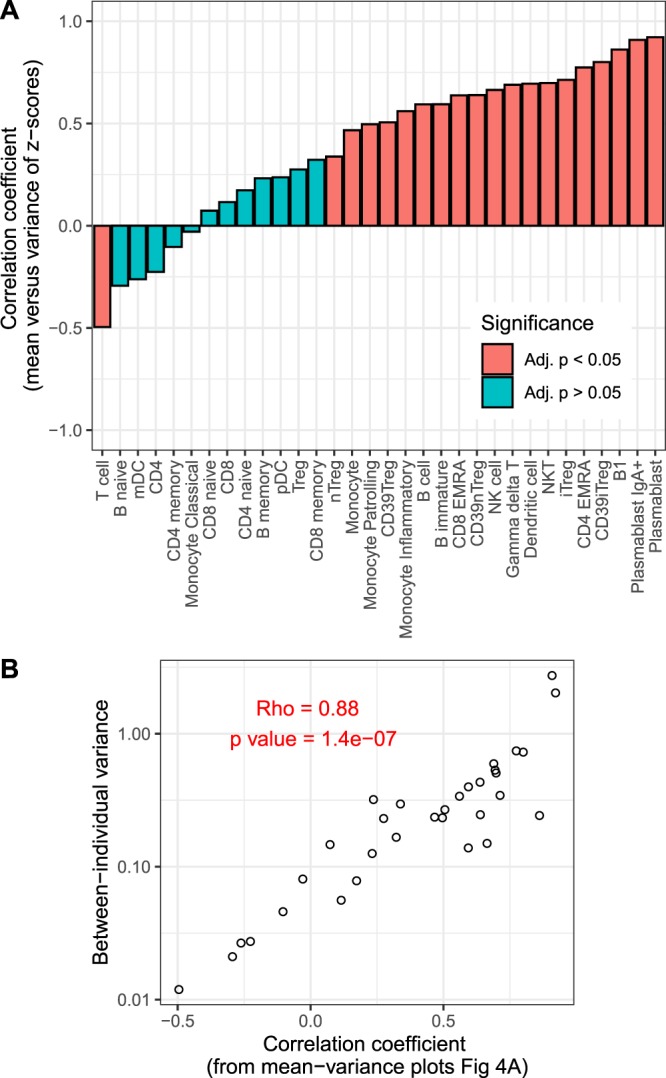
(**A**) Correlation coefficients (Spearman) of correlation between intra-individual mean and variance of all the immune subsets and **(B)** it’s relationship with rank order of inter-individual variance.

We rank ordered immune subsets according to the degree of inter-individual variance shown in Fig. 1A (which reflects total population variance), and also according to the correlation coefficient observed in mean-variance plots (shown in Fig. 4A) (a measure of strength of susceptibility to short-term environmental fluctuations). We tested for the relationship between these two measures and found that immune subsets that show high inter-individual variance also showed high correlation coefficients for their within-individual mean-variance plots (Fig. 4B), suggesting that immune subsets that show high variance in the population may be driven by short-term fluctuations in environmental factors.

### Lack of clustering of individuals based on baseline parameters and degree of fluctuation

**Figure 5:**
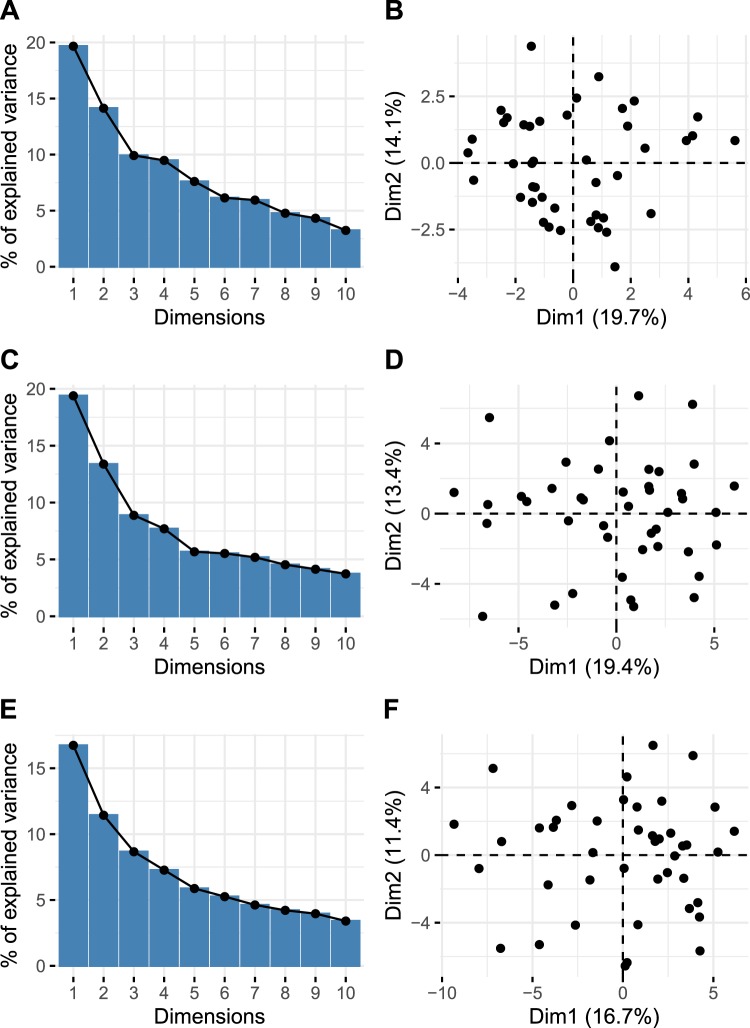
Principal component analysis (PCA) plots showing clustering of individuals considering their baseline cellular immune parameter levels (**A**,**B**), degree of fluctuation (**C**,**D**), and both the baseline levels and degree of fluctuation (**E**,**F**). A, C and E show scree plots that indicate the % of explained variance with the first two principal components.

We asked whether individuals vary continuously in the population or show distinct clusters based on steady-state baseline immune profiles and temporal fluctuations in all immune parameters considered together. To test this, we performed principal component analysis (PCA) on individuals based on their baseline levels and degree of fluctuation of all immune subsets quantified (Fig. 5). The first 2 PCs accounted for nearly 20–30% of variance (Fig. 5A,C,E). This analysis revealed absence of clearly defined clusters of individuals defined by their baseline levels (Fig. 5B), degree of fluctuation (Fig. 5D) or both considered together (Fig. 5F). The few outliers that were seen when the degree of fluctuation was considered (Fig. 5D) were not different in terms of their gender or age distribution (Supplementary Fig. S6).

### Correlation of immune parameters among individuals reveal biological relationships between immune subsets

**Figure 6:**
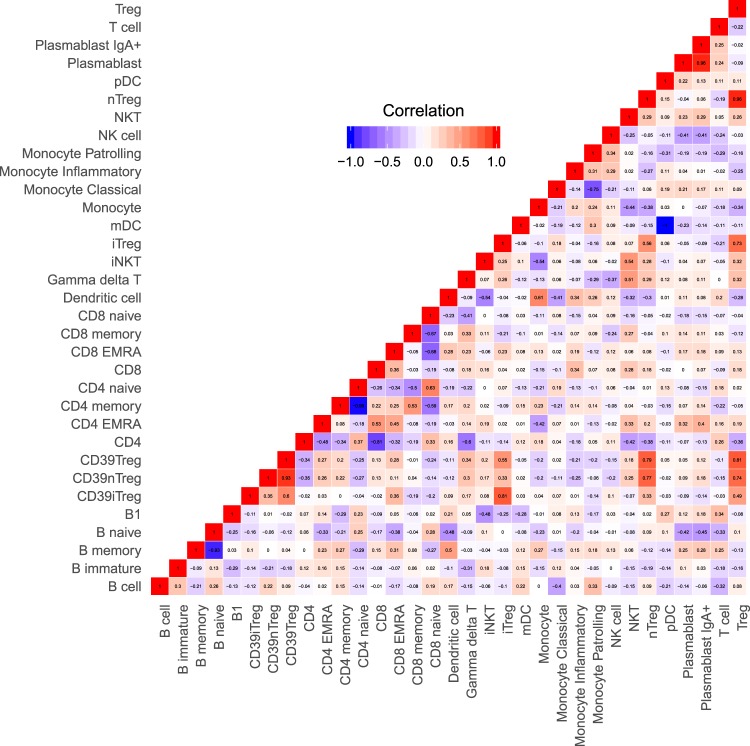
Correlation heatmap showing the correlation of frequency of each subset with others. Each subset is described as % of parent gate as defined in Supplementary Dataset Table S1. Raw data of this figure is tabulated in Supplementary Dataset Table S8.

**Figure 7:**
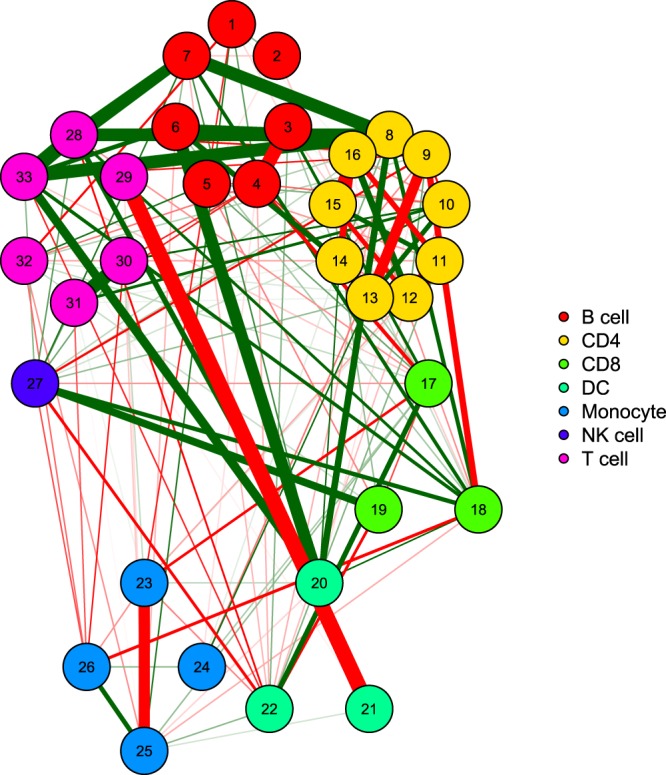
Correlation between subsets (shown in Fig. 6) shown as a network showing inter-relationships between subsets. Positive correlations are indicated by green connections and negative correlations are indicated by red connections. Width of the connecting line indicates the strength of correlation. Nodes represent the cell subsets grouped with the colours indicated. The annotation for each cell subset is as follows: 1. B cell, 2. B immature, 3. B memory, 4. B naive, 5. B1 B cell, 6. Plasmablast, 7. Plasmablast IgA + , 8. CD39 + iTreg, 9. CD39 + nTreg, 10. CD39 + Treg, 11. CD4 EMRA, 12. CD4 memory, 13. CD4 naive, 14. iTreg, 15. nTreg, 16. Treg, 17. CD8 EMRA, 18. CD8 memory, 19. CD8 naive, 20. Dendritic cell, 21. mDC, 22. pDC, 23. Monocyte, 24. Monocyte Classical, 25. Monocyte Inflammatory, 26. Monocyte Patrolling, 27. NK cell, 28. CD4, 29. CD8, 30. Gamma delta T cell, 31. iNKT, 32. NKT, 33. T cell.

We attempted to understand the heterogeneity at the individual-level by asking whether individuals who show extremes of steady-state levels in one immune parameter also show extreme baseline levels in other immune parameters. To address this, we calculated the correlation matrix of the baseline levels of multiple immune parameters tested (Fig. 6 and Supplementary Dataset Table S8), also shown as a network of correlation matrix (Fig. 7). There were significant positive correlations between DC and monocytes, CD4 memory and CD8 memory, Gamma-delta T and NKT, CD4 EMRA and CD8 EMRA (Supplementary Fig. S7 and Supplementary Dataset Table S8). Other than the negative correlations expected between subsets sharing a same parent gate, there were significant negative correlations between CD4 and gamma-delta T cells, iNKT cells and monocytes, dendritic cells and iNKT, naive B cells and DCs, B1 and iNKT, monocytes and NKT cells (Supplementary Fig. S8 and Supplementary Dataset Table S8).

We also similarly tested whether individuals who show extreme fluctuation in one immune parameter also show extreme fluctuation (either high or low) in other immune parameters (Supplementary Fig. S9, Supplementary Dataset Table S9), and find that apart from subsets that share the same parent gate there are no significant, strong correlations, implying that the degree to which parameters fluctuate is unique to individual, and not influenced by biological relationship between immune subsets.

### Subsets that show lower population-variance and lower short-term fluctuations tend to show concordance in siblings

**Figure 8:**
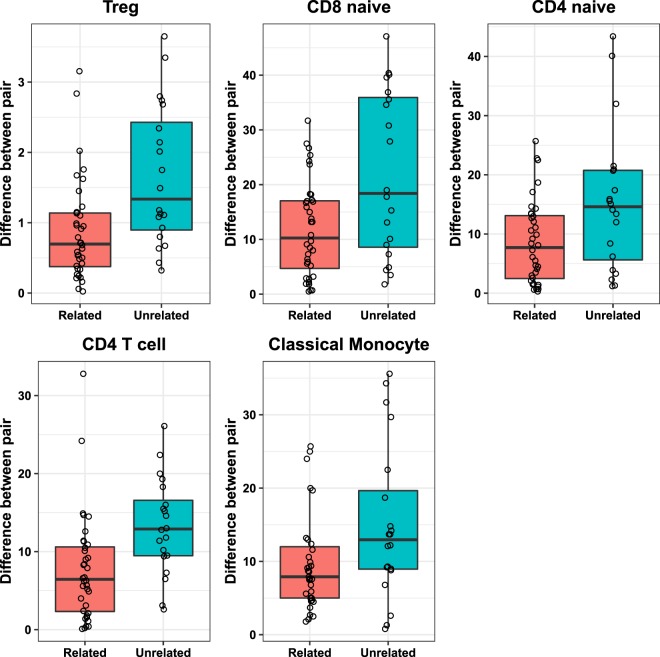
Distance between immune subsets in unrelated pairs and sibling pairs. Each subset is described as % of parent gate as defined in Supplementary Dataset Table S1. P-values are tabulated in Supplementary Dataset Table S11.

We tested whether factors other than short-term environmental fluctuations (cumulative childhood environmental factors and/or genetic factors) could contribute to determining the ‘baseline’ immune phenotypes by phenotyping sibling-pairs from 37 families (anonymized raw data in Supplementary Dataset Table S10). Average age of siblings was 31.8 (SD = 12.7) and male:female ratio was 38:42. We quantified the distance between each immunological parameter in each sibling pair and compared it with unrelated pairs (derived from one time point of the serially-bled cohort) and calculated the statistical significance using permutation test and re-sampling (Supplementary Fig. S10, Supplementary Dataset Table S11). This analysis revealed that immune subsets that showed lower variance in the population and lower short-term fluctuations (CD4 T cell, CD4 memory, CD8 memory, Classical monocyte, Treg) also showed high concordance in siblings (Fig. 8, Supplementary Dataset Table S11), suggesting they are not driven by environmental exposures and highly likely to be determined by heredity or early life exposures. On the other hand, subsets like plasmablasts, CD4 EMRA, inflammatory monocytes and B1 B cells did not show concordance in siblings (Supplementary Dataset Table S11, Supplementary Fig. S11).

## Discussion

Our study aimed to quantify heterogeneity between individuals in their peripheral blood cellular immune phenotype. We identify cell subsets that show relatively higher variability compared to others, and suggest that genetic differences between individuals and their environmental exposure related heterogeneity could be contributors for such heterogeneity between subsets. We also attempt to understand the determinants of baseline cellular immune levels in serial-bleeds of healthy individuals and identify subsets that show considerable variation within-individuals from those that show minimal variability within individuals in the short term.

One of the limitations of our study in the quantification of within-individual variability is that we could not reliably quantify absolute cell counts, since estimates of total leukocyte counts and differential counts needed to be performed using fresh blood. Thus, estimates of absolute counts will be expected to be confounded by error due to technical factors, unlike flow cytometric estimates of cell frequencies from cryopreserved PBMC samples which were quantified together. Our estimates of within-individual variability using the two methods we adopted (namely, using variance components model and using hypothesis testing approach using permutation test) were broadly in agreement. A recent publication that characterised longer duration of temporal stability of immune subsets also show broad agreement with what we report^15^. Our analysis was confined to analysis of short term fluctuation (a period of one year) and hence avoids confounding due to ageing-associated alterations that could contribute to within-individual fluctuations^15^. The narrow age group that we choose in the cohort also permits quantifying inter-individual variability without being confounded by immunological ageing.

Longitudinal immune profile estimates are difficult to interpret due to additional confounding factors introduced by variability in PBMC processing, storage and batch-variations in staining^16^. We tried to address these issues by running technical replicates with each batch of flow cytometry samples. We find relatively higher technical variances for iNKT cells, monocytes, immature B cells and iTregs. We are hence cautious in interpretation of within- and between-individual variability of these subsets. Longitudinal profiling allowed us to test seasonality in trends of immune parameters, but this analysis is limited by the fact that measurements of all individuals were not started at the same time point. Absence of seasonality could be because of near constant exposure of environmental factors in urban settings in our cohort. Also, any effect of seasonality in exposure to infections is nullified because by design, the study excluded recently infected or sick individuals to obtain true baseline phenotypes.

One of the major limitations of our study is that it does not identify specific genetic variants or environmental factors (eg. CMV status, latent infections) that could contribute to immune variation between individuals. However, the present study was designed to quantify inter-individual and intra-individual variability and to test the degree of familial associations and is underpowered for extensive genome wide association analysis or testing for associations with multiple environmental determinants. Nevertheless, the data from our study helps establish baselines and will help design larger observational and longitudinal cohorts as well as sibling/twin cohorts to test for genetic and specific environmental associations.

Our analysis of testing for correlation between baseline levels and degree of variation of cellular subset levels in serial-sampled individuals identified immune subsets that differ with respect to each other in their strength of correlation, which we hypothesise could be determined by environmental susceptibility. Nonetheless, we acknowledge that interpreting such complex data from natural conditions across multiple subsets is prone to unexpected confounders and errors. Hence conservative interpretations of the results of our analysis are advisable. In this regard, the subsets that we found to be most stable within individuals (eg. memory T cells) also show concordance in siblings and we have shown previously that they are cell-autonomously regulated using bone marrow chimera approach in independent inbred strains of mice^17^. Recent studies have shown that within-individual stability in T cells is not confined to lymphocyte-pool sizes, but to gene expression patterns^18^ and functional activation profiles as well^19^.

Inter-relationships between leukocyte subsets have been traditionally studied using reductionist models and fail to capture the complexity that is seen *in-vivo* in individuals in their natural environments. Our analysis of correlation-based networks identified interrelationships between immune subsets that could have biological basis, and hence is of interest for future mechanistic studies. It will be interesting to explore whether such inter-relationships between immune subsets will hold true and whether strengths of inter-relationships will differ in different categories of individuals (eg. nutritional states, geographical differences etc) that could alter environmental factors that influence biological connectedness between cell subsets.

Consistent with a previous study^10^, our cohort also did not find discreet differences between individuals considering their baseline immunophenotype as well as degree of variability, although prominent outliers of individuals could be seen when the degree of fluctuation was considered, more than what is seen when baseline immune phenotypes were considered. This suggests that groups of prominent outlier individuals could be identified based on their ‘variability’ of baseline immune phenotype. Whether higher variability (fluctuation) in some individuals are contributed by their environmental exposure fluctuations or their innate sensitivity to fluctuations is an interesting question to explore. This could potentially have been addressed if siblings could be followed up to test whether their degree of fluctuation show concordance. We could not identify any gender or age associations for outliers who show different patterns of fluctuation. We have tried to address whether the individuals who show high variability for one subset also show variability for other subsets and we find that variability in subsets are independent of each other. Thus, a conservative interpretation of our data could be that, apart from noise due to technical factors, the outliers that we see for individual temporal fluctuations could be biological outliers or a consequence of sampling error in our relatively small cohort. Of course, considering the complexity of several steps involved in the assay, we cannot entirely rule out technical artefacts presenting as outliers in such analysis.

The issue of heterogeneity in immune parameters brings up questions whether heterogeneity in immune parameters predict responses to infectious challenge. Recent studies^8,20,21^ have suggested that this could be the case at least for some vaccines. This implies that understanding immune heterogeneity and it’s determinants is valuable for appreciating differences between populations for vaccine responses as well as it’s potential implications in being a platform for natural selection in human populations.

To summarise, we characterise immune heterogeneity in a cohort of young adults from India and suggest potential determinants of such heterogeneity and inter-relationship between immune parameters.

## Methods

### Ethics clearances

The study was approved by Institutional Ethics Committees of National Institute of Immunology approval number IHEC/AKS/45/2013) and All India Institute of Medical Sciences (approval number IEC/NP-471/2013). All methodologies used were in accordance with approved guidelines. All experimental protocols used in this study were approved by Institutional Ethics Committee (Human Research) of National Institute of Immunology and All India Institute of Medical Sciences. Informed consent was obtained in written form from all participants before their enrolment into the study.

### Informed consent

Informed written consent was obtained from all participants before their enrolment into the study.

### Human subjects

Healthy adult volunteers were recruited into the study with informed written consent and were bled 3-monthly for 12 months (total of 4 bleeds). All volunteers were residents of National Capital Region (NCR), India, during the period of the study. The recruitment period was from 2013 to 2015. Volunteers with acute or chronic illness, drug intake, recent vaccination (within 6 months) or pregnancy (in women) were excluded. Any volunteers with clinical evidence of infections such as fever and respiratory discharge/diarrhoea in the previous two weeks, or with a history of any vaccination in the previous six months, were excluded. Ten ml peripheral blood was collected in heparinised tubes. All samples were collected between 9–11 AM post-breakfast. The study was approved by institutional ethics committee of National Institute of Immunology (NII). All protocols were in accordance with approved guidelines. Informed consent was obtained before participation. For sibling study, 37 families were recruited, in which at least two siblings were willing for participation. The family-based study was approved by institutional ethics committees of NII and All India Institute of Medical Sciences (AIIMS). Inclusion and exclusion criteria were similar to that mentioned above. Informed written consent was obtained for all family members who participated in the study. Details of the two cohorts (longitudinal study cohort and sibling cohort) are also described in a previous publication^17^.

### Flow cytometry

Blood samples were processed within three hours after collection. Total leukocyte counts were obtained from hemocytometer and differential counts were obtained by examination of peripheral smear. Density gradient centrifugation was used to separate PBMCs, which were then washed, counted and divided into aliquots of about 1 million cells per ml per vial in 10% DMSO in bovine serum and preserved frozen in liquid nitrogen. For flow cytometry, PBMCs were thawed, washed and incubated with the antibodies. A single control sample was run along with each experiment to ensure that gating is comparable across experiments. Absolute cell counts were performed by calculating total leukocyte count in fresh blood samples using a haemocytometer. Briefly, blood was diluted in Turk’s fluid for RBC lysis and fixation and staining of WBCs, and loaded into haemocytometer. Cells were counted in the areas designated for counting of WBCs. Differential counts were calculated by preparing a peripheral smear, staining using Geimsa and morphologically classifying 100 WBCs on a counter. Total leukocyte count calculation and peripheral smear was performed in house by a researcher (PK) trained in hematological laboratory methods under the supervision of a qualified Pathologist (SBP). The cell counts of individual subsets were then back-calculated from the frequencies of individual subsets obtained from flow cytometry. All samples were acquired in BD FACSVerse and analysed using flowjo (Treestar).

### Statistical analysis

Raw data was exported from flowjo (Treestar) as comma separated value (csv) files and analysed using R(3.5.1) and Rstudio (V1.1.463). All the raw spreadsheets and R codes used for the analysis can be accessed from author’s github public repository (https://github.com/savitprabhu/immune_variation_ms2).

We calculated between-individual variance, within-individual variance, and variance in technical replicates as follows: each observation value was divided by the mean value of the subset. This ‘centers’ the mean of all subsets, while still preserving differences in variance structure between the subsets. Variances calculated from mean-centered values are thus comparable across subsets with different population sizes. The same procedure was done for technical controls. Non-parametric bootstrapping (resampling) methods were used for comparison of intra-individual variances with between-individual variances. Variance components (linear mixed effects) model was constructed using lme4 package in R. For comparison of within-individual mean (baseline levels) versus variance (degree of fluctuation) across the different subsets, each observation was first transformed (scaled) to generate z-scores against the mean value of each immune subset. This normalizes for the differences in subset-population sizes, and adjusts for differences in variances seen between-individuals for the various subsets. For each individual, mean and variances of z-scores (representing within-individual baselines and within-individual temporal fluctuations respectively) were then calculated and Spearman’s correlation coefficients were estimated.

Non-parameteric tests were used for comparison of immune phenotypes for gender differences, and correction for multiple comparison was performed to obtain adjusted p-values (Bonferroni). Correlations between subsets (baseline levels as well as degree of fluctuation) were estimated as Spearman’s coefficient. Adjustments were made for p-values to account for multiple comparisons (using false discovery rate (FDR) method). Non-parametric bootstrapping (resampling) methods were used for comparison of sibling pairs vs unrelated pairs.

## Supplementary information

Supplementary information

Supplementary Dataset 1

Supplementary Dataset 2

Supplementary Dataset 3

Supplementary Dataset 4

Supplementary Dataset 5

Supplementary Dataset 6

Supplementary Dataset 7

Supplementary Dataset 8

Supplementary Dataset 9

Supplementary Dataset 10

Supplementary Dataset 11

## References

[1] Brodin P, Davis MM (2016). Human immune system variation. Nature Reviews Immunology.

[2] Liston A, Carr EJ, Linterman MA (2016). Shaping Variation in the Human Immune System. Trends in Immunology.

[3] Roederer M (2015). The Genetic Architecture of the Human Immune System: A Bioresource for Autoimmunity and Disease Pathogenesis. Cell.

[4] Orrù V (2013). Genetic variants regulating immune cell levels in health and disease. Cell.

[5] Labuda LA (2014). Differences in Innate Cytokine Responses between European and African Children. PLoS ONE.

[6] Brodin P (2015). Variation in the Human Immune System Is Largely Driven by Non-Heritable Influences. Cell.

[7] Carr EJ (2016). The cellular composition of the human immune system is shaped by age and cohabitation. Nature Immunology.

[8] Tsang JS (2014). Global Analyses of Human Immune Variation Reveal Baseline Predictors of Postvaccination Responses. Cell.

[9] Satija R, Shalek AK (2014). Heterogeneity in immune responses: from populations to single cells. Trends in Immunology.

[10] Kaczorowski KJ (2017). Continuous immunotypes describe human immune variation and predict diverse responses. Proceedings of the National Academy of Sciences.

[11] Aguirre-Gamboa R (2016). Differential effects of environmental and genetic factors on T and B cell immune traits. Cell reports.

[12] Bakker, O. B. *et al*. Integration of multi-omics data and deep phenotyping enables prediction of cytokine responses. *Nature immunology* **1** (2018). 10.1038/s41590-018-0121-3 PMC6022810 29784908

[13] Patin E (2018). Natural variation in the parameters of innate immune cells is preferentially driven by genetic factors. Nature Immunology.

[14] Mangino M, Roederer M, Beddall MH, Nestle FO, Spector TD (2017). Innate and adaptive immune traits are differentially affected by genetic and environmental factors. Nature Communications.

[15] Alpert, A. *et al*. A clinically meaningful metric of immune age derived from high-dimensional longitudinal monitoring. *Nature medicine* **1** (2019). 10.1038/s41591-019-0381-y PMC6686855 30842675

[16] Brodin P, Duffy D, Quintana-Murci L (2019). A call for blood—in human immunology. Immunity.

[17] Chawla AS (2018). Cell-intrinsic regulation of peripheral memory-phenotype T cell frequencies. PloS one.

[18] Lu Y (2016). Systematic analysis of cell-to-cell expression variation of T lymphocytes in a human cohort identifies aging and genetic associations. Immunity.

[19] Ye CJ (2014). Intersection of population variation and autoimmunity genetics in human T cell activation. Science.

[20] Lingblom CM, Kowli S, Swaminathan N, Maecker HT, Lambert SL (2018). Baseline immune profile by CYTOF can predict response to an investigational adjuvanted vaccine in elderly adults. Journal of translational medicine.

[21] Goronzy JJ (2001). Value of immunological markers in predicting responsiveness to influenza vaccination in elderly individuals. Journal of virology.

