# Supplementary material for: Characterization of biological variation of peripheral blood immune cytome in an Indian cohort
Source: Sci Rep. 2019 Oct 14;9:14735. doi: 10.1038/s41598-019-51294-7 (PMC6791881; doi:10.1038/s41598-019-51294-7)
Supplement: Supplementary file 1 — Supplementary information [file 41598_2019_51294_MOESM1_ESM.pdf]

SUPPLEMENTARY INFORMATION

Characterization of biological variation of peripheral blood immune cytome in an Indian cohort

Parna Kanodia, Gurvinder Kaur, Poonam Coshic, Kabita Chatterjee, Teresa Neeman, Anna George, Satyajit Rath, Vineeta Bal, Savit B Prabhu.

Supplementary Figures 1 to 11

SUPPLEMENTARY FIGURES

Characterization of biological variation of peripheral blood immune cytome in an Indian cohort

Parna Kanodia, Gurvinder Kaur, Poonam Coshic, Kabita Chatterjee, Teresa Neeman, Anna George, Satyajit Rath, Vineeta Bal, Savit B Prabhu.

Supplementary Figure 1

Normal ranges of immune cell frequencies (A) and counts (B) of the longitudinal study cohort (n=43). Boxplots indicate median and interquartile range. Upper and lower whiskers extend till 1.5 times the interquartile range from 3rd and 1st quartile respectively. Outliers are shown as dots. Immune cell frequencies are expressed as frequency (%) of parent gate as indicated in Supplementary Table S1. Parent gates are indicated with different colours. Absolute counts were back-calculated from Total Leukocyte Count (TLC) values and expressed per cubic milliliter of blood.

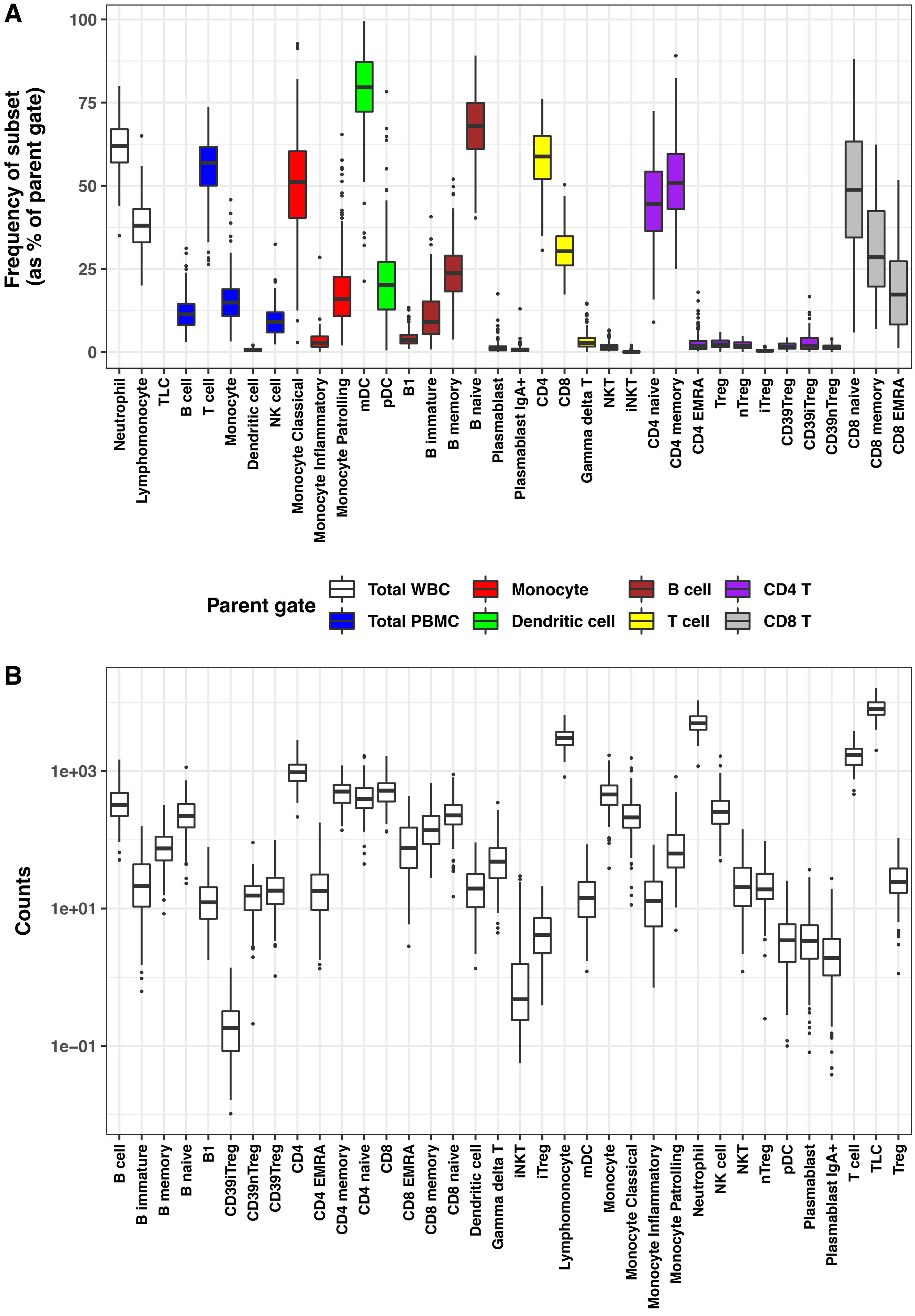

A
Frequency of subset
(as % of parent gate)
100
75
50
25
0
Neutrophil
Lymphomonocyte
TLC
B cell
T cell
Monocyte
Dendritic cell
NK cell
Monocyte Classical
Monocyte Inflammatory
Monocyte Patrolling
mDC
pDC
B1
B immature
B memory
B naive
Plasmablast
Plasmablast IgA+
CD4
CD8
Gamma delta T
NKT
iNKT
CD4 naive
CD4 memory
CD4 EMRA
Treg
nTreg
iTreg
CD39Treg
CD39iTreg
CD39nTreg
CD8 naive
CD8 memory
CD8 EMRA
Parent gate
Total WBC
Monocyte
B cell
CD4 T
Total PBMC
Dendritic cell
T cell
CD8 T
B
Counts
1e+03
1e+01
1e−01
B cell
B immature
B memory
B naive
B1
CD39iTreg
CD39nTreg
CD39Treg
CD4
CD4 EMRA
CD4 memory
CD4 naive
CD8
CD8 EMRA
CD8 memory
CD8 naive
Dendritic cell
Gamma delta T
iNKT
iTreg
Lymphomonocyte
mDC
Monocyte
Monocyte Classical
Monocyte Inflammatory
Monocyte Patrolling
Neutrophil
NK cell
NKT
nTreg
pDC
Plasmablast
Plasmablast IgA+
T cell
TLC
Treg

SUPPLEMENTARY FIGURES

Characterization of biological variation of peripheral blood immune cytome in an Indian cohort

Parna Kanodia, Gurvinder Kaur, Poonam Coshic, Kabita Chatterjee, Teresa Neeman, Anna George, Satyajit Rath, Vineeta Bal, Savit B Prabhu.

Supplementary Figure 2

Seasonal variation of immune cell subsets. In each panel, x-axis indicates the month of bleed and y-axis indicates the z-score transformed raw data of frequency of immune subsets (expressed as % of parent gate as indicated in Supplementary Table S1). Each dot represents the z-score of frequency of immune subset of an individual. Trend line (blue) with confidence intervals (grey shaded region) indicate the seasonal trend calculated using linear regression. The months on x-axis are spread across the years 2014 and 2015, the time of sample collection.

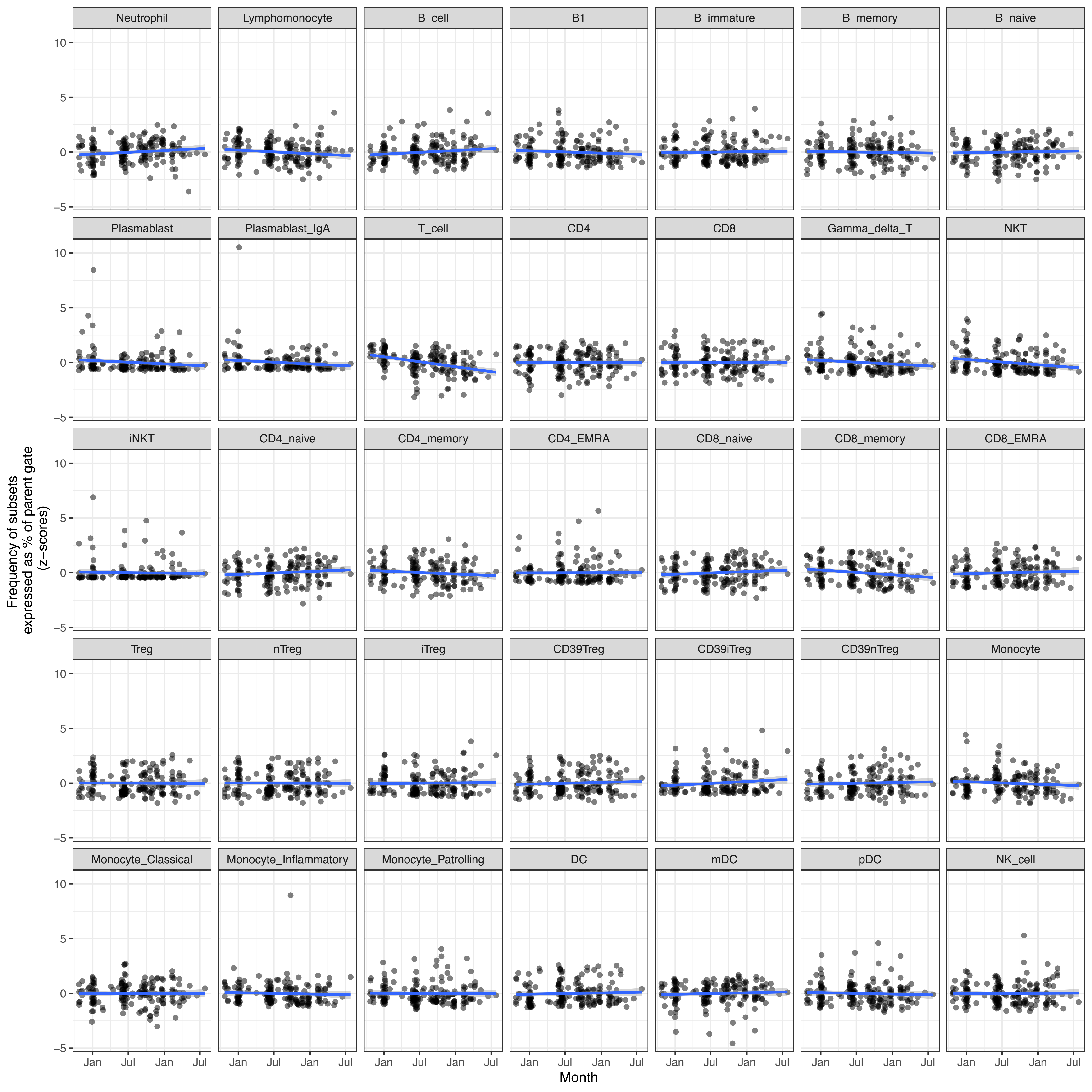

Neutrophil
Lymphomonocyte
B_cell
B1
B_immature
B_memory
B_naive
Plasmablast
Plasmablast_IgA
T_cell
CD4
CD8
Gamma_delta_T
NKT
iNKT
CD4_naive
CD4_memory
CD4_EMRA
CD8_naive
CD8_memory
CD8_EMRA
Treg
nTreg
iTreg
CD39Treg
CD39iTreg
CD39nTreg
Monocyte
Monocyte_Classical
Monocyte_Inflammatory
Monocyte_Patrolling
DC
mDC
pDC
NK_cell
Frequency of subsets expressed as % of parent gate (z−scores)
10
5
0
−5
Jan
Jul
Jan
Jul
Month

SUPPLEMENTARY FIGURES

Characterization of biological variation of peripheral blood immune cytome in an Indian cohort

Parna Kanodia, Gurvinder Kaur, Poonam Coshic, Kabita Chatterjee, Teresa Neeman, Anna George, Satyajit Rath, Vineeta Bal, Savit B Prabhu.

Supplementary Figure 3

The first page shows plots of variance in technical replicates along with within-individual and between-individual variance.
The second page shows plots of relationship between between-individual variance and within-individual variance after adjusting for variance in technical replicates. The third and fourth pages show absence of correlation between technical variability and within-individual variance (page 3) or between-individual variance (page 4).

The fifth page shows population size of each subset (ie. Mean frequencies) on x-axis and technical variability in technical replicates on y-axis. There is no association, suggesting that rarer populations do not necessarily show higher variances in our technical replicates.

In all figures, each dot represents an immune subset. Solid line indicates best fit line of the linear regression model with standard errors shaded.

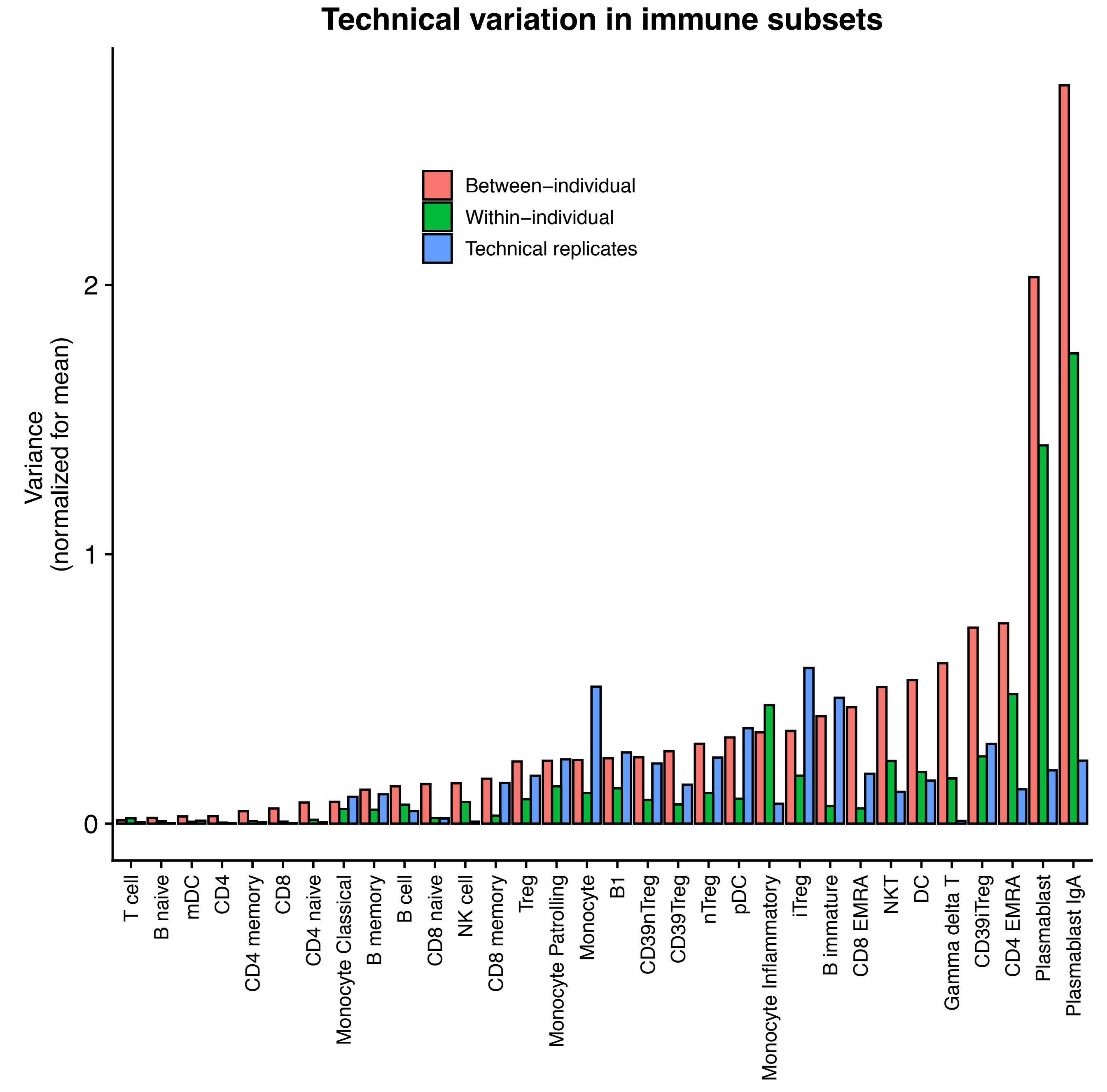

Technical variation in immune subsets
Variance
(normalized for mean)
0
1
2
Between−individual
Within−individual
Technical replicates
T cell
B naive
mDC
CD4
CD4 memory
CD8
CD4 naive
Monocyte Classical
B memory
B cell
CD8 naive
NK cell
CD8 memory
Treg
Monocyte Patrolling
Monocyte
B1
CD39nTreg
CD39Treg
nTreg
pDC
Monocyte Inflammatory
iTreg
B immature
CD8 EMRA
NKT
DC
Gamma delta T
CD39iTreg
CD4 EMRA
Plasmablast
Plasmablast IgA

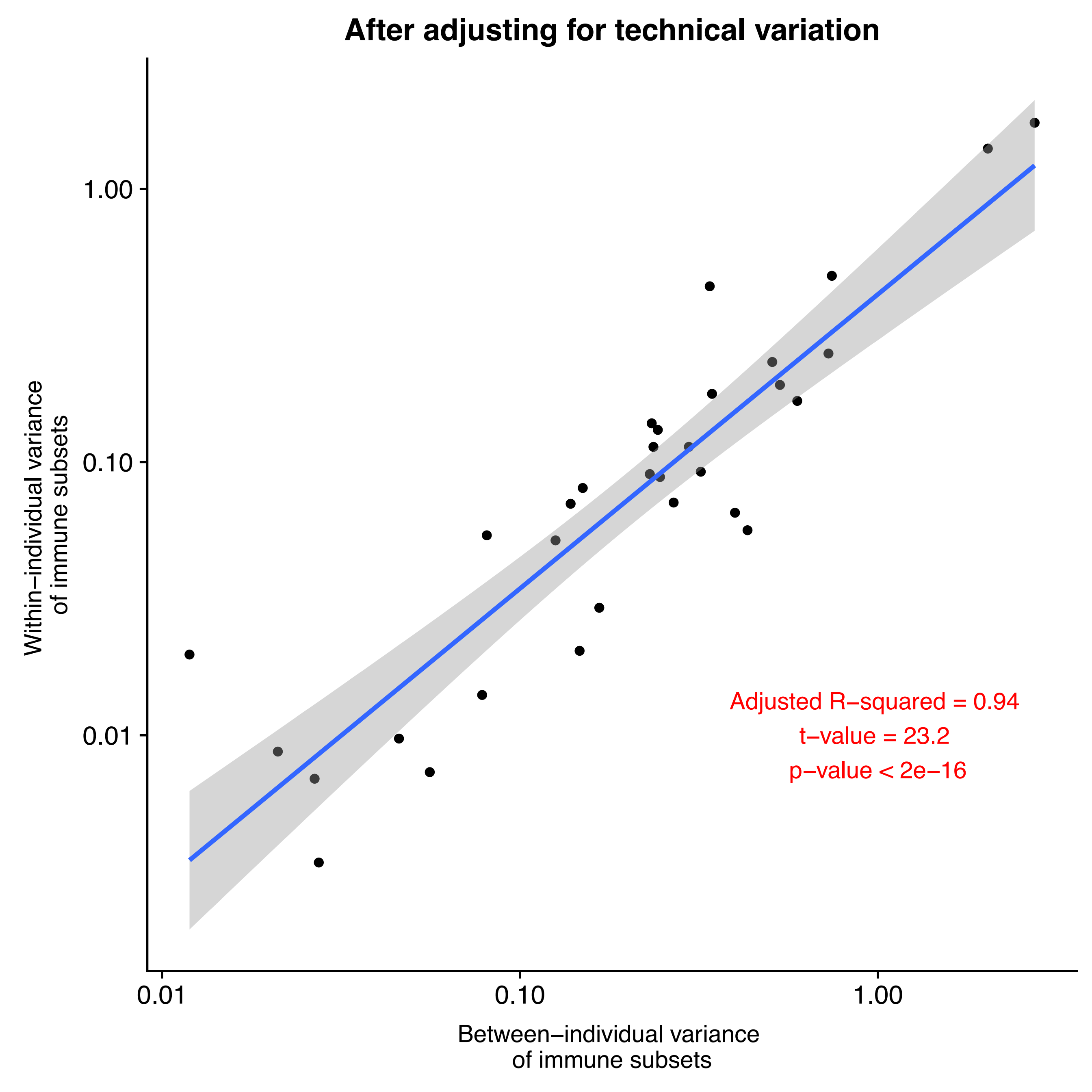

After adjusting for technical variation
Within−individual variance
of immune subsets
1.00
0.10
0.01
0.01
0.10
1.00
Between−individual variance
of immune subsets
Adjusted R−squared = 0.94
t−value = 23.2
p−value < 2e−16

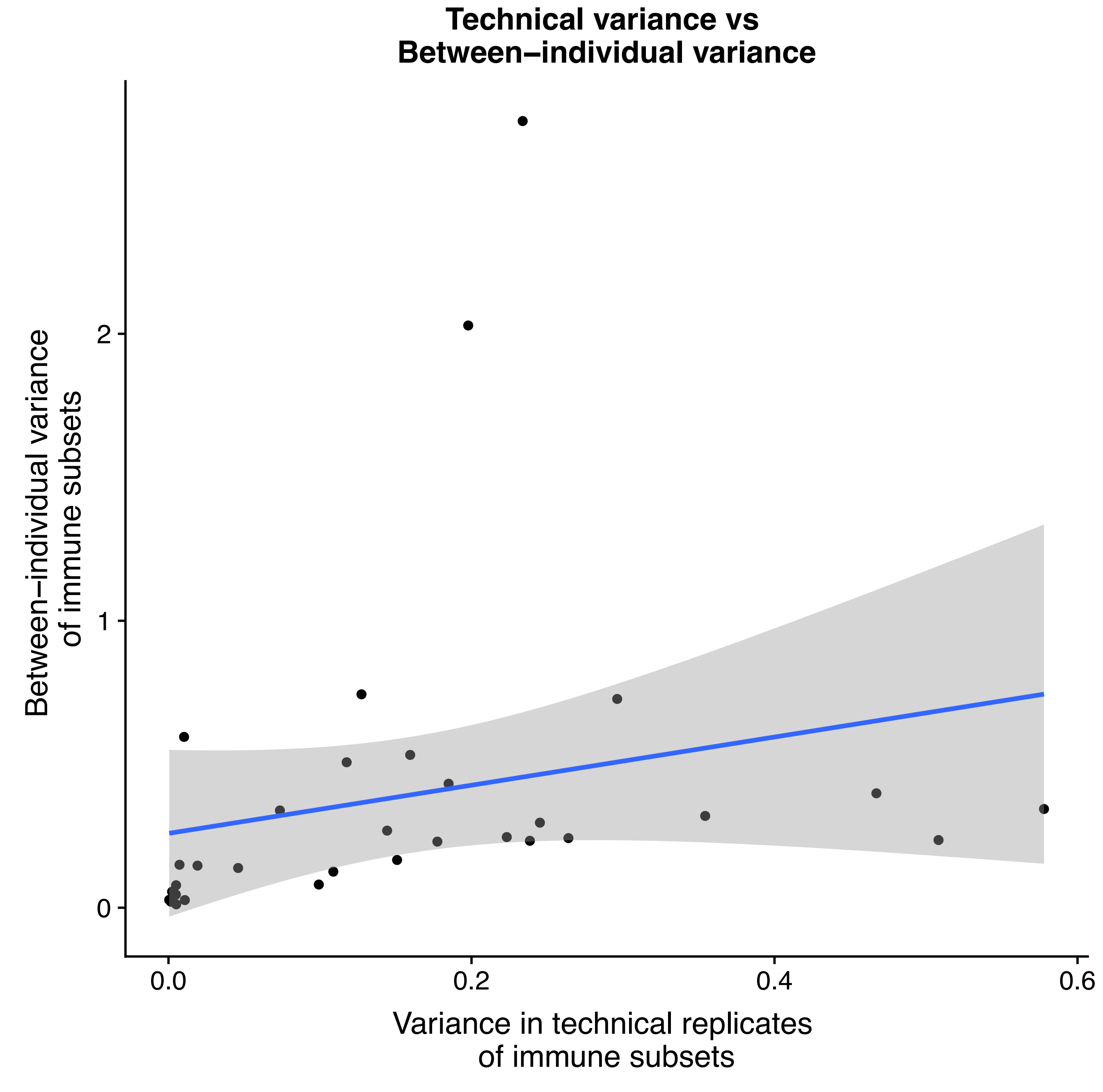

Technical variance vs
Between−individual variance
Between−individual variance
of immune subsets
2
1
0
0.0
0.2
0.4
0.6
Variance in technical replicates
of immune subsets

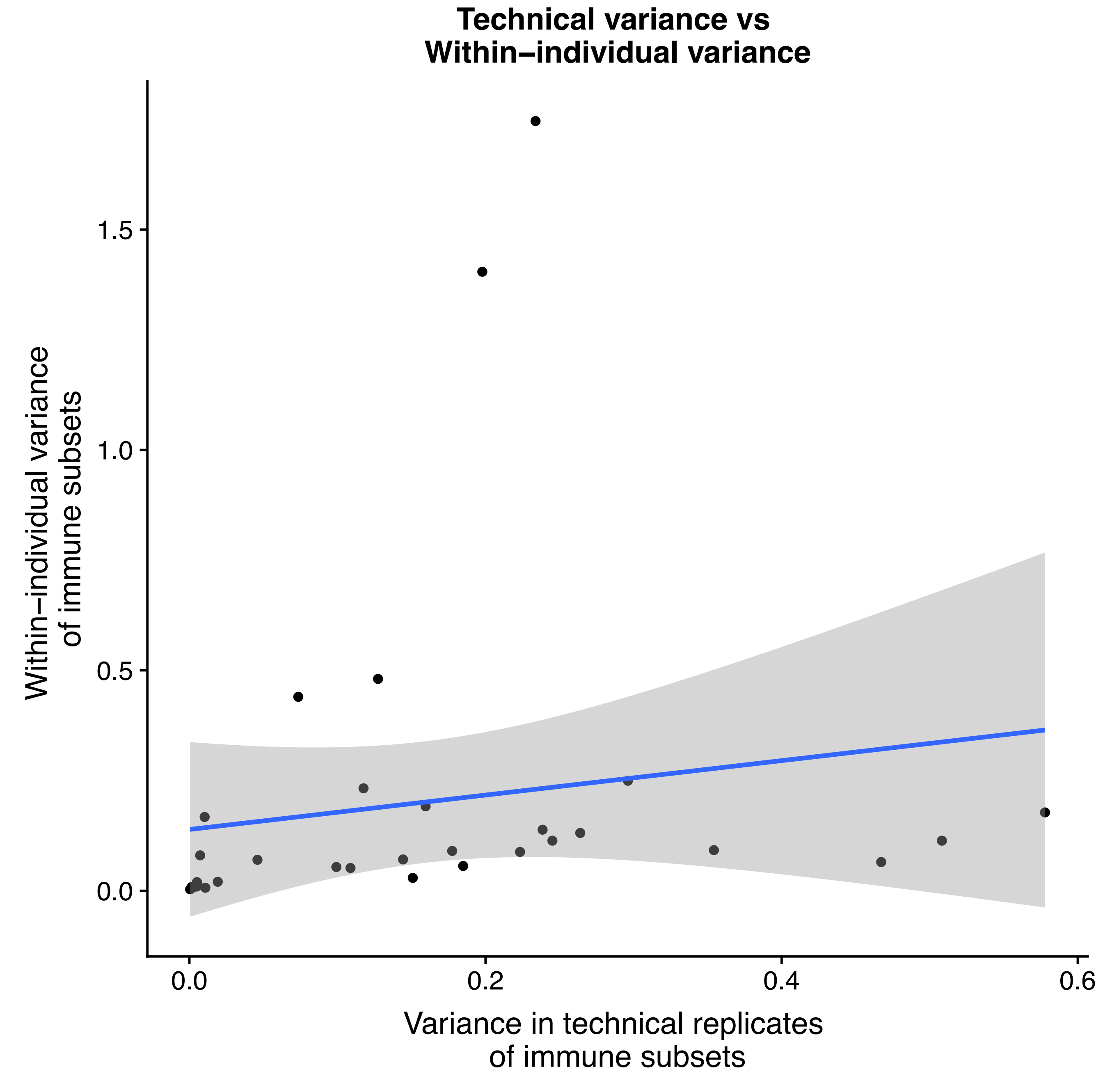

Technical variance vs
Within−individual variance
Within−individual variance
of immune subsets
0.0
0.5
1.0
1.5
0.0
0.2
0.4
0.6
Variance in technical replicates
of immune subsets

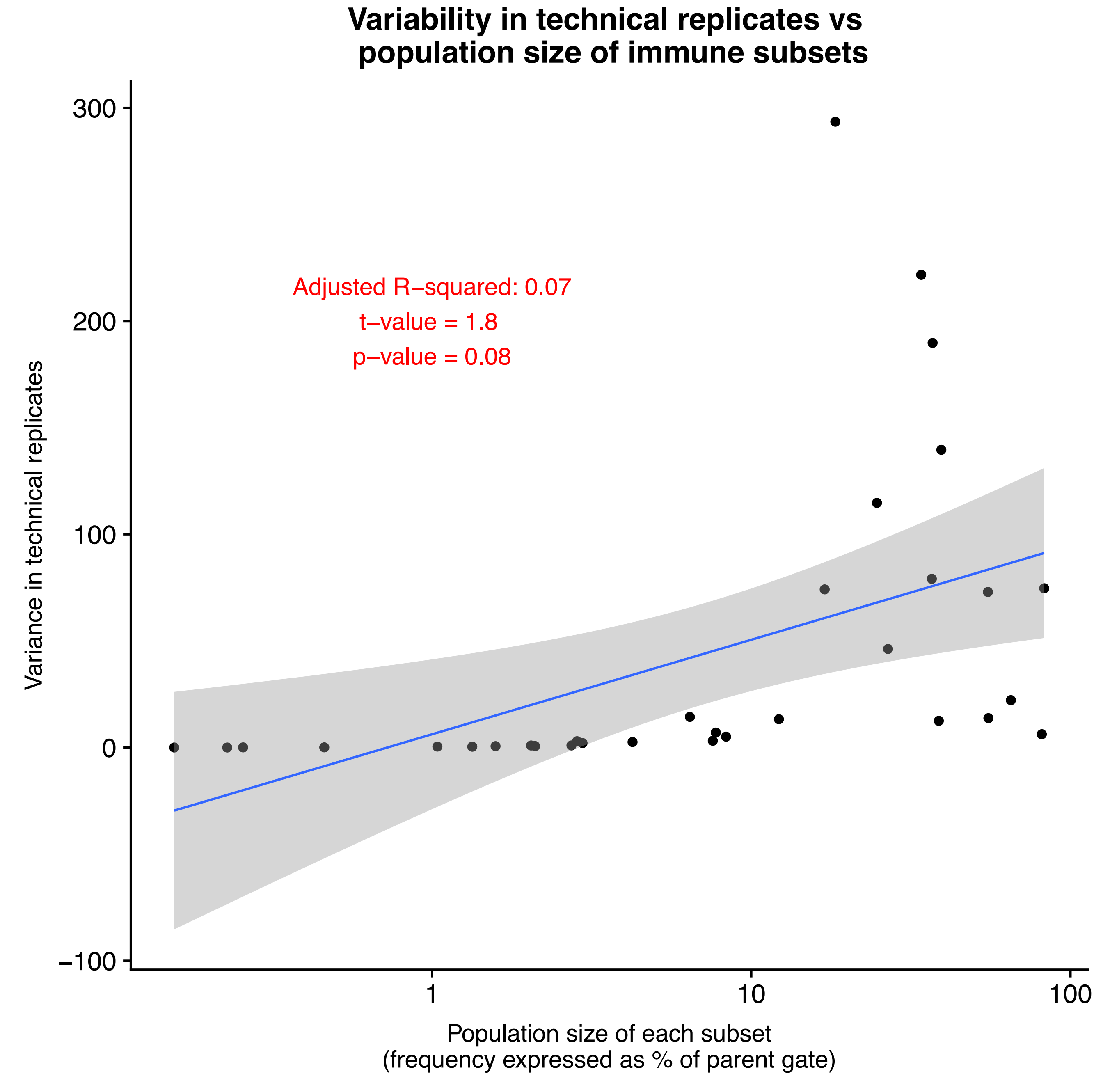

Variability in technical replicates vs
population size of immune subsets
Adjusted R−squared: 0.07
t−value = 1.8
p−value = 0.08
Variance in technical replicates
300
200
100
0
−100
1
10
100
Population size of each subset
(frequency expressed as % of parent gate)

SUPPLEMENTARY FIGURES

Characterization of biological variation of peripheral blood immune cytome in an Indian cohort

Parna Kanodia, Gurvinder Kaur, Poonam Coshic, Kabita Chatterjee, Teresa Neeman, Anna George, Satyajit Rath, Vineeta Bal, Savit B Prabhu.

Supplementary Figure 4

The first page shows hypothetical models showing contribution of within-individual variation contributing to between-individual variation. Subsequent pages show boxplots showing comparison of within-individual variability and between-individual variability for all subsets. In each figure, the panel on the left indicates the distribution of values between individuals and shows the extend of inter- individual variation. The panel on the right shows intra-individual variation in each individual over 4 time points over one year. The individuals are rank ordered for better visualization of trends. Boxplots indicate median and interquartile range. Upper and lower whiskers extend till 1.5 times the interquartile range from 3rd and 1st quartile respectively. Outliers are shown as dots. Immune subset frequencies are calculated as % of parent gate described in Supplementary Table 1.

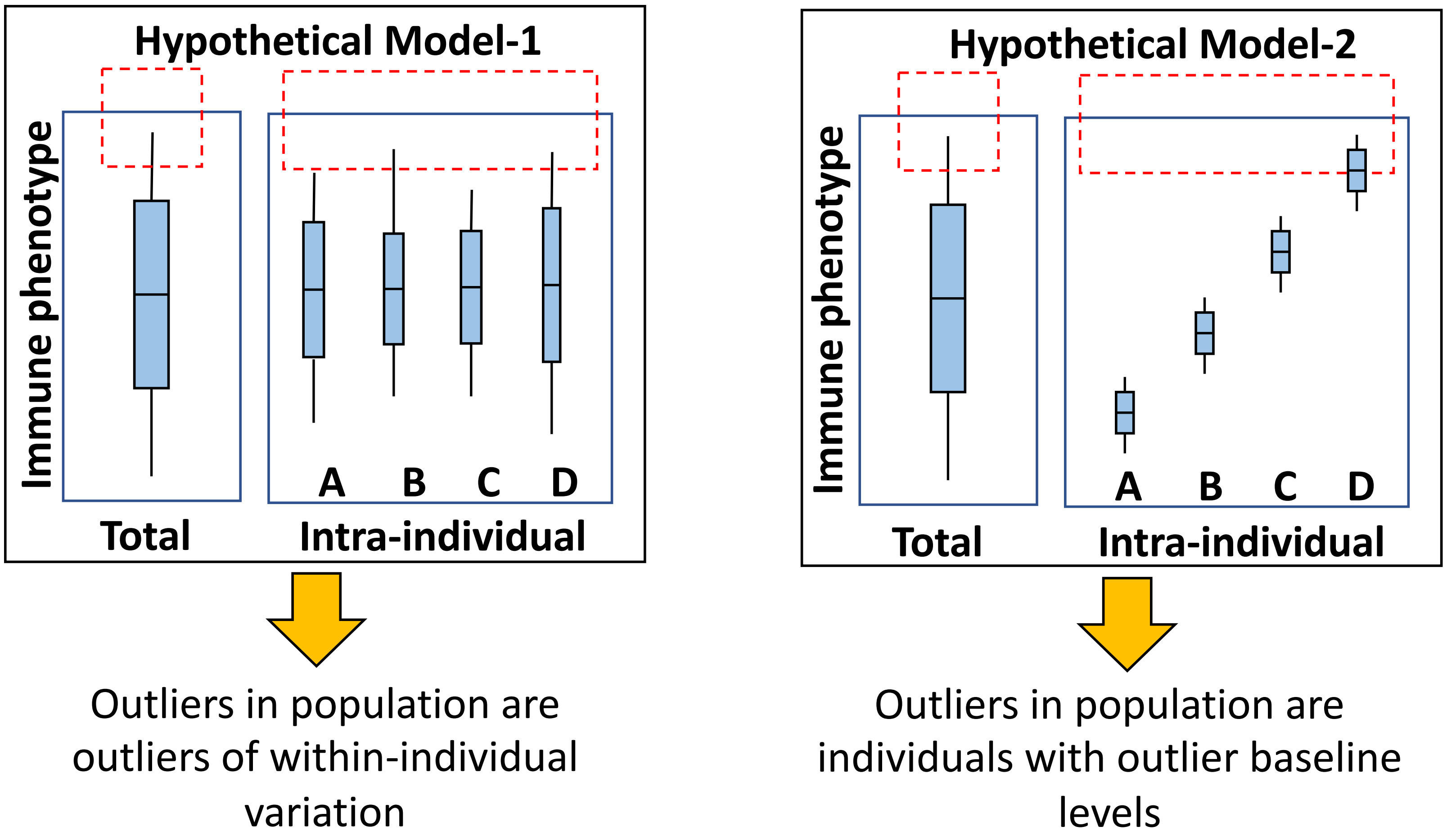
Hypothetical Model-1
Immune phenotype
Total
A
B
C
D
Intra-individual
Outliers in population are outliers of within-individual variation
Hypothetical Model-2
Immune phenotype
Total
A
B
C
D
Intra-individual
Outliers in population are individuals with outlier baseline levels

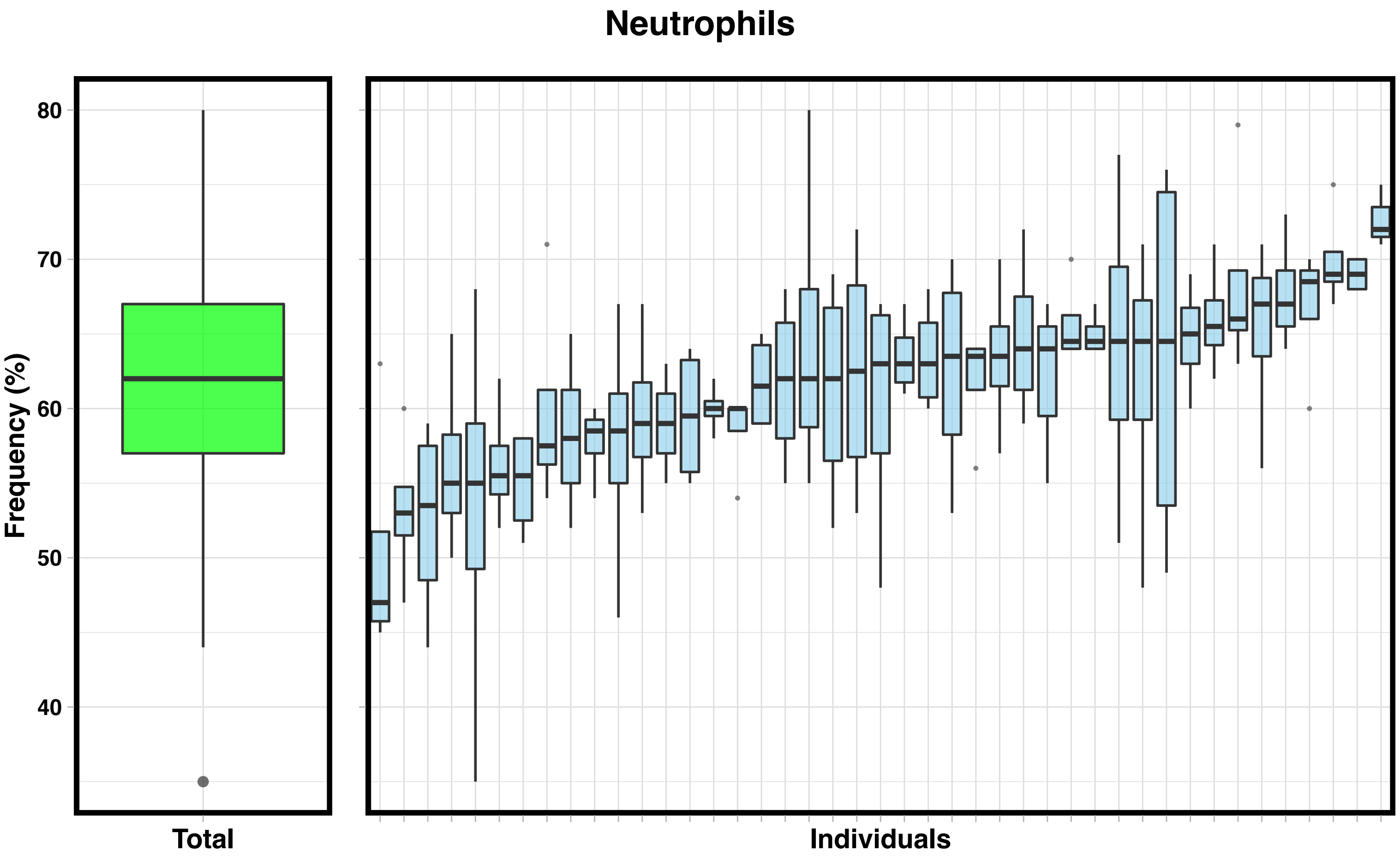
Neutrophils
Frequency (%)
80
70
60
50
40
Total
Individuals

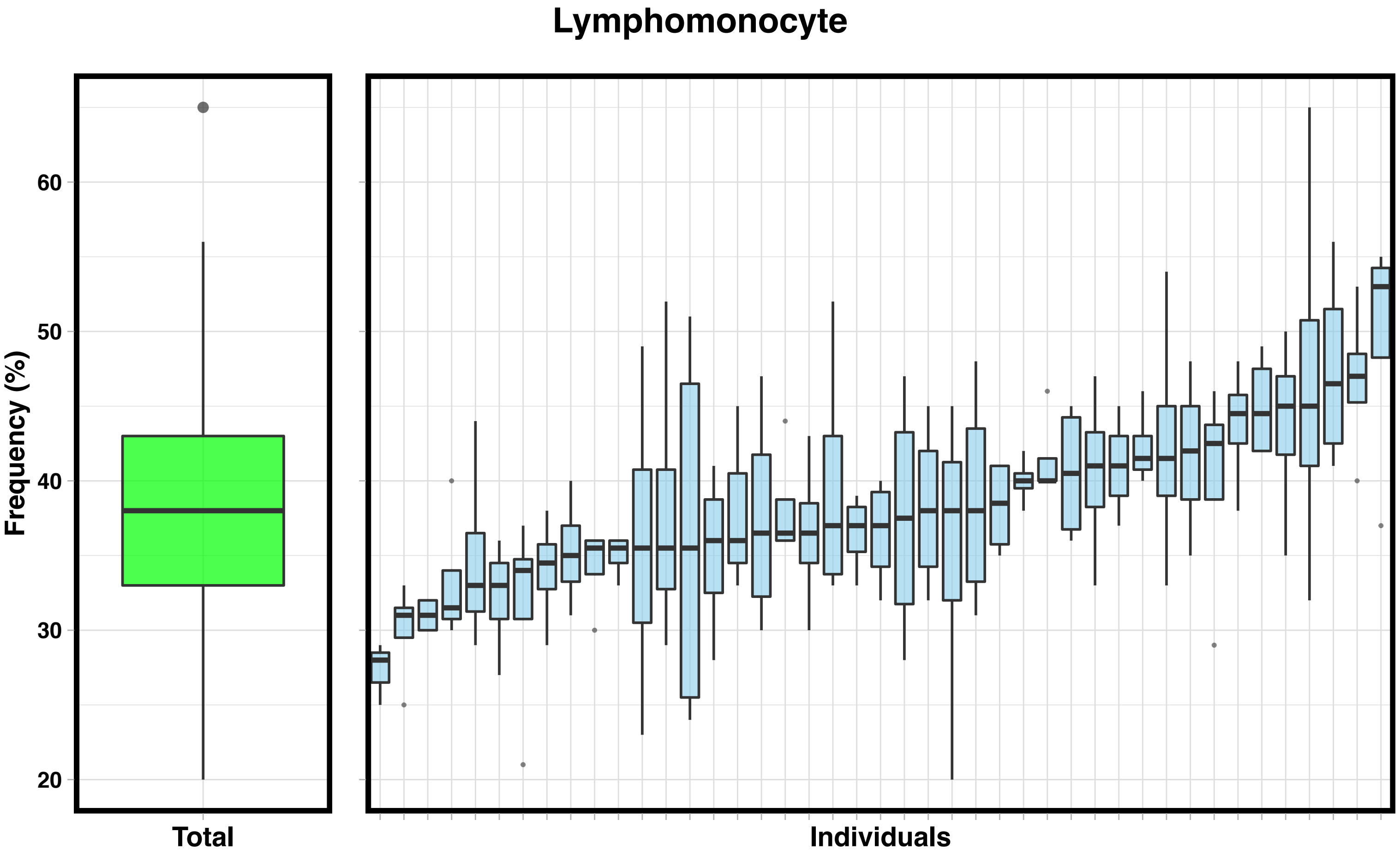

Lymphomonocyte
Frequency (%)
60
50
40
30
20
Total
Individuals

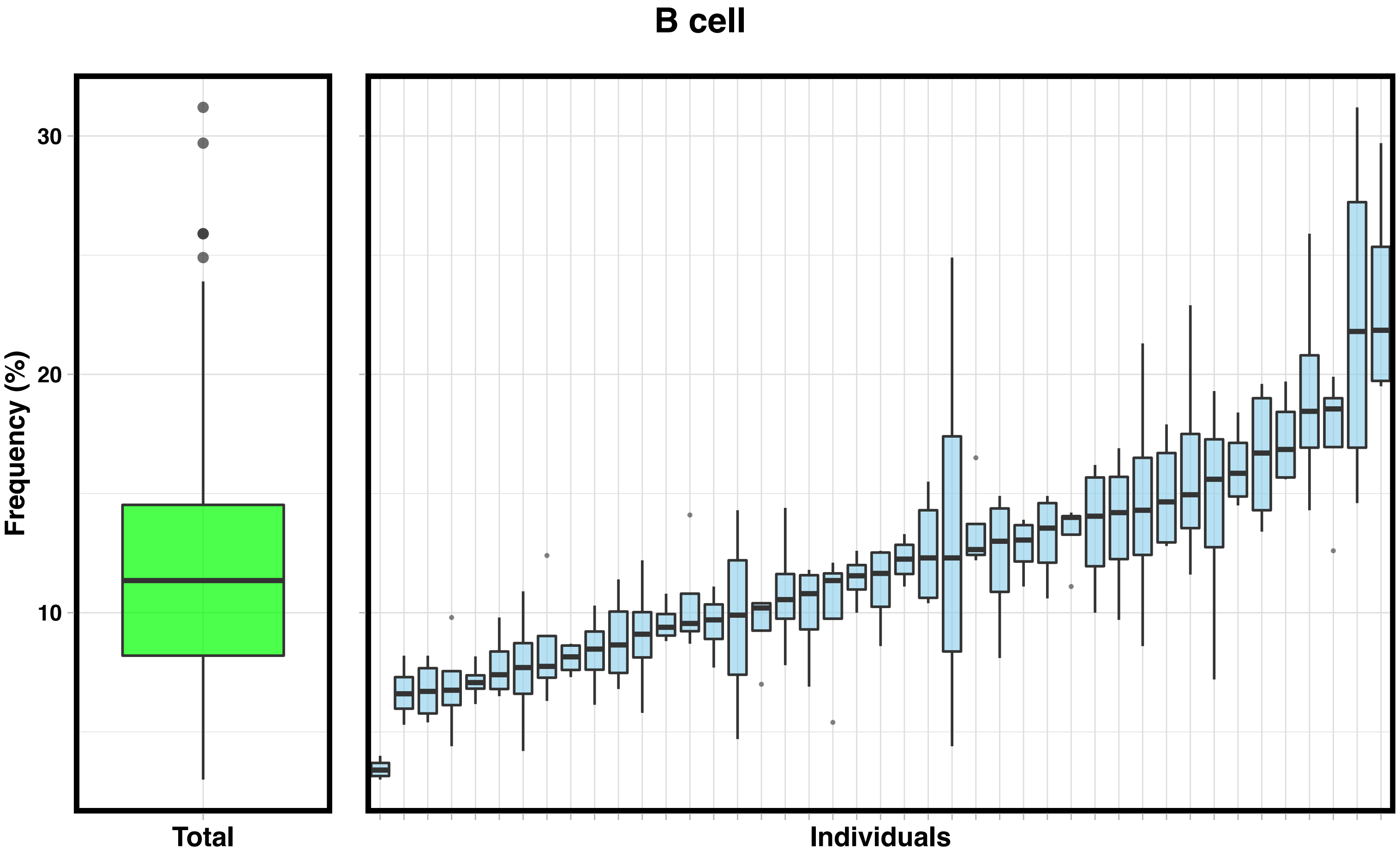
B cell
Frequency (%)
30
20
10
Total
Individuals

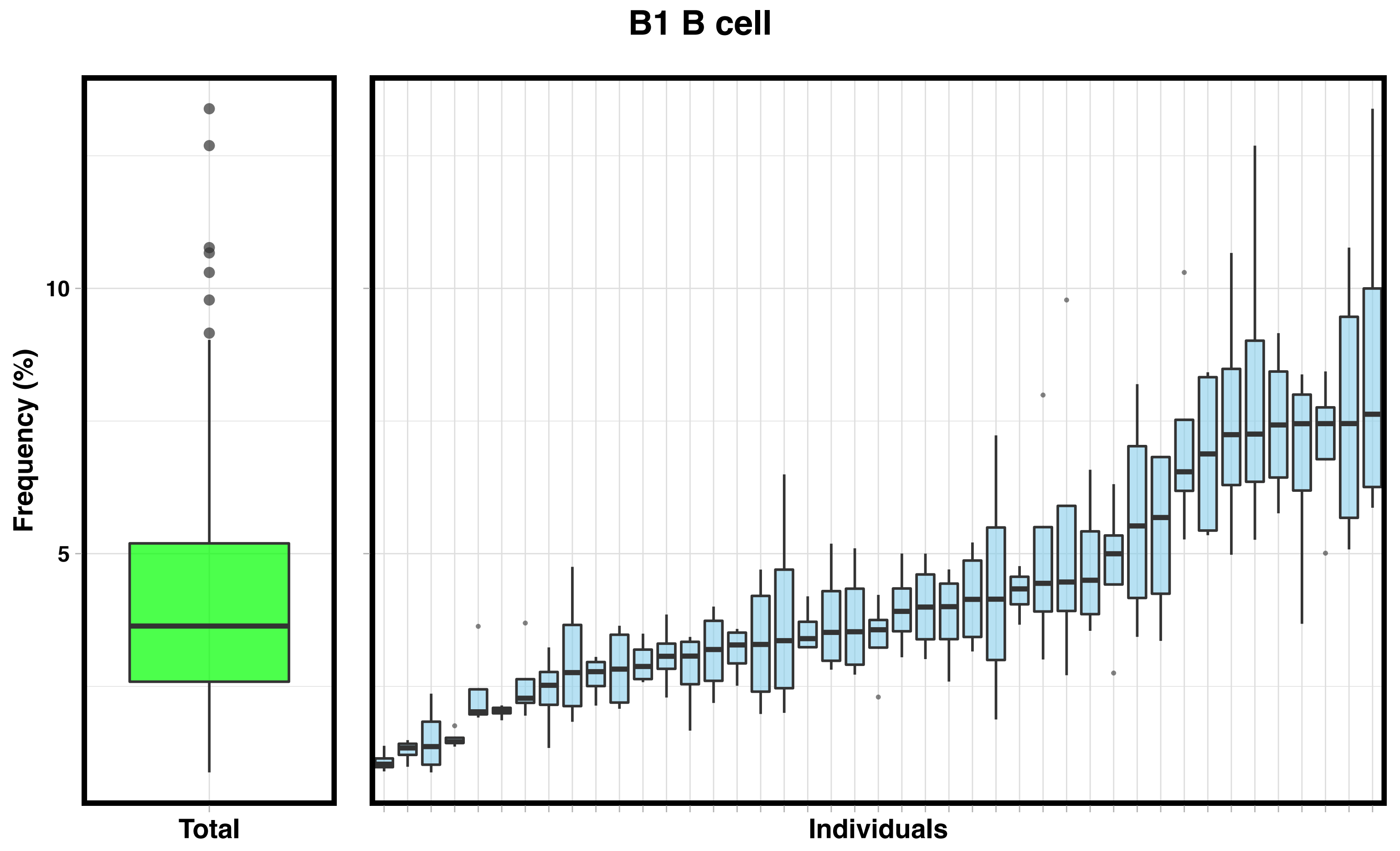

B1 B cell
Frequency (%)
10
5
Total
Individuals

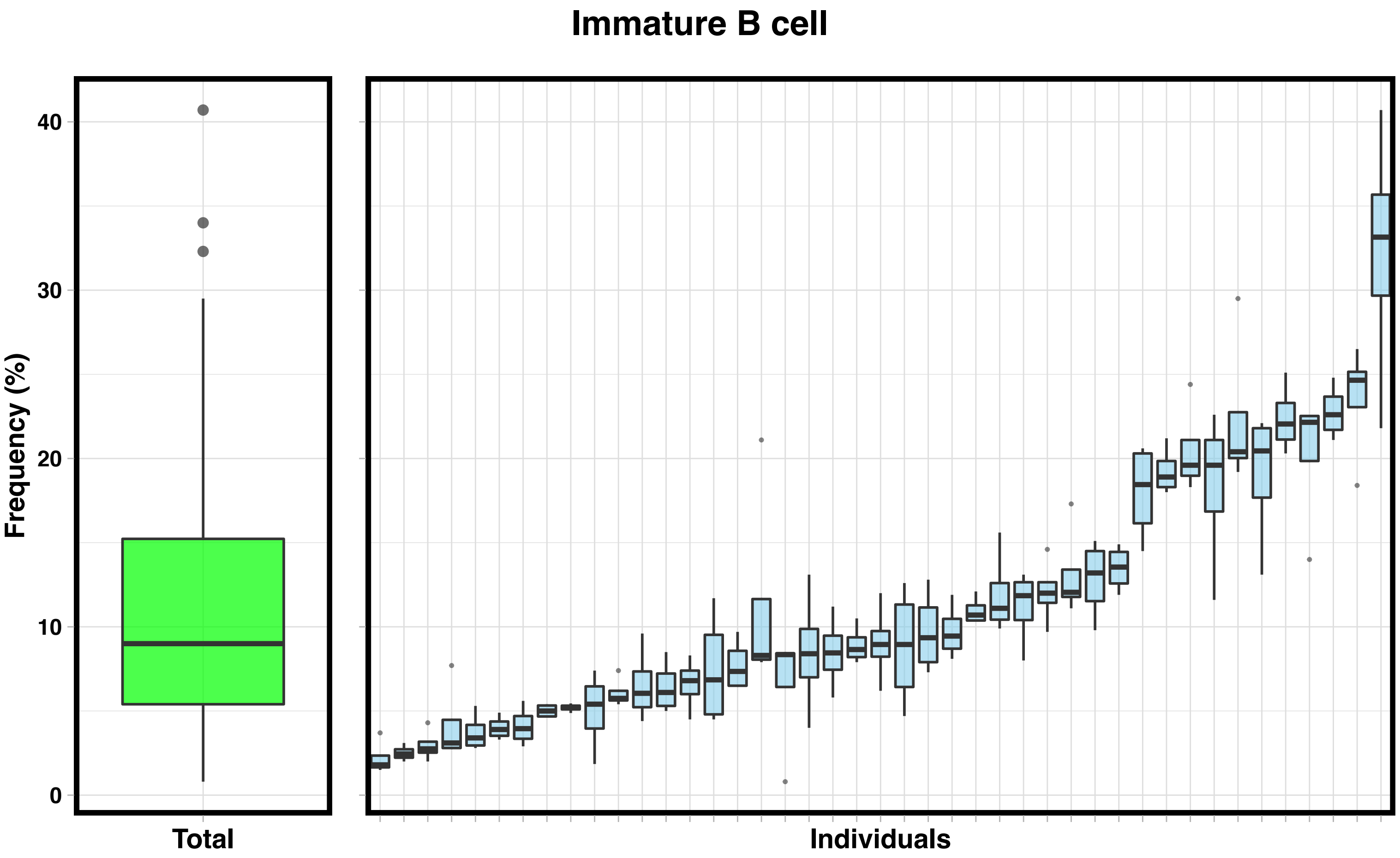
Immature B cell
Frequency (%)
40
30
20
10
0
Total
Individuals

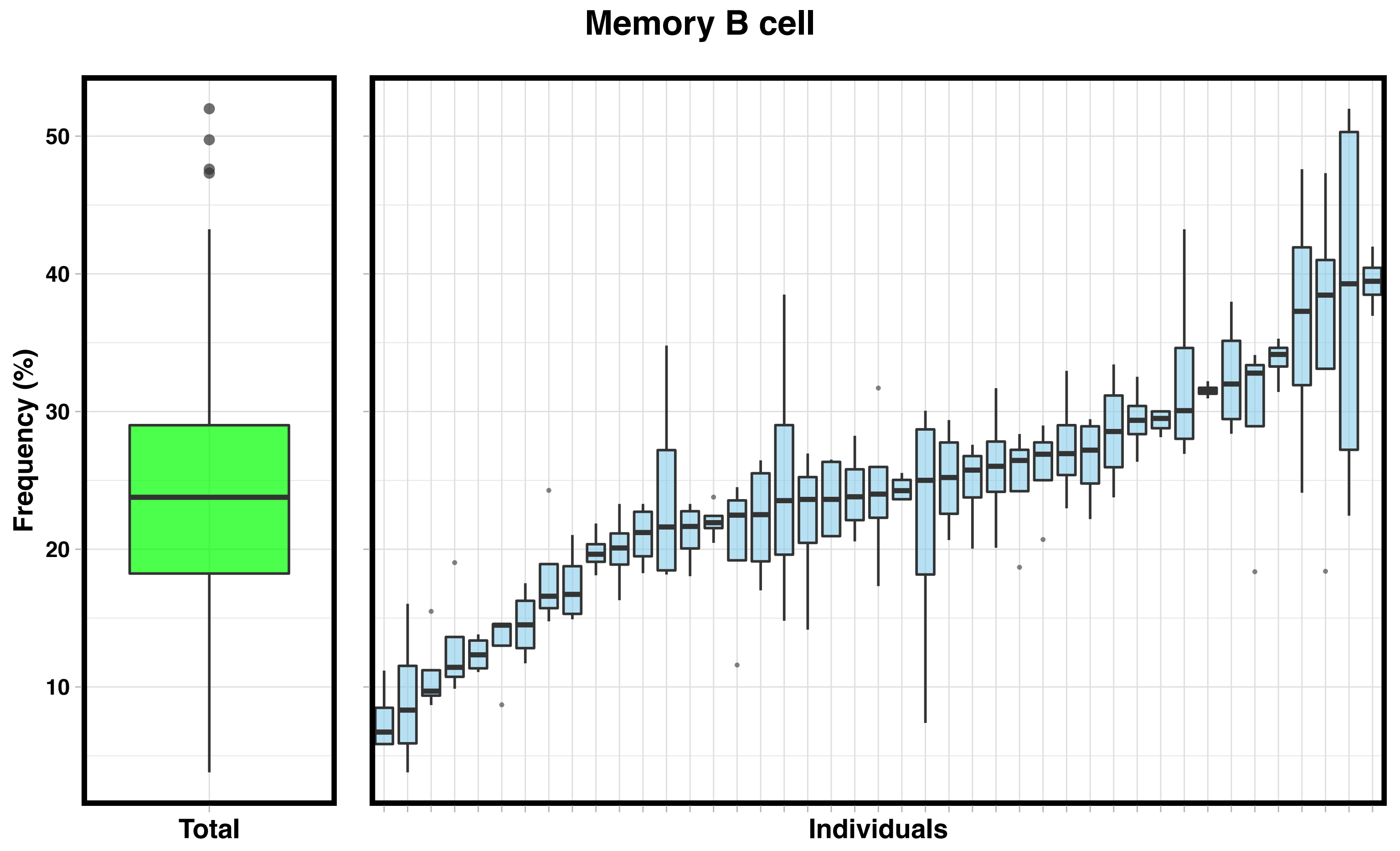

Memory B cell
Frequency (%)
50
40
30
20
10
Total
Individuals

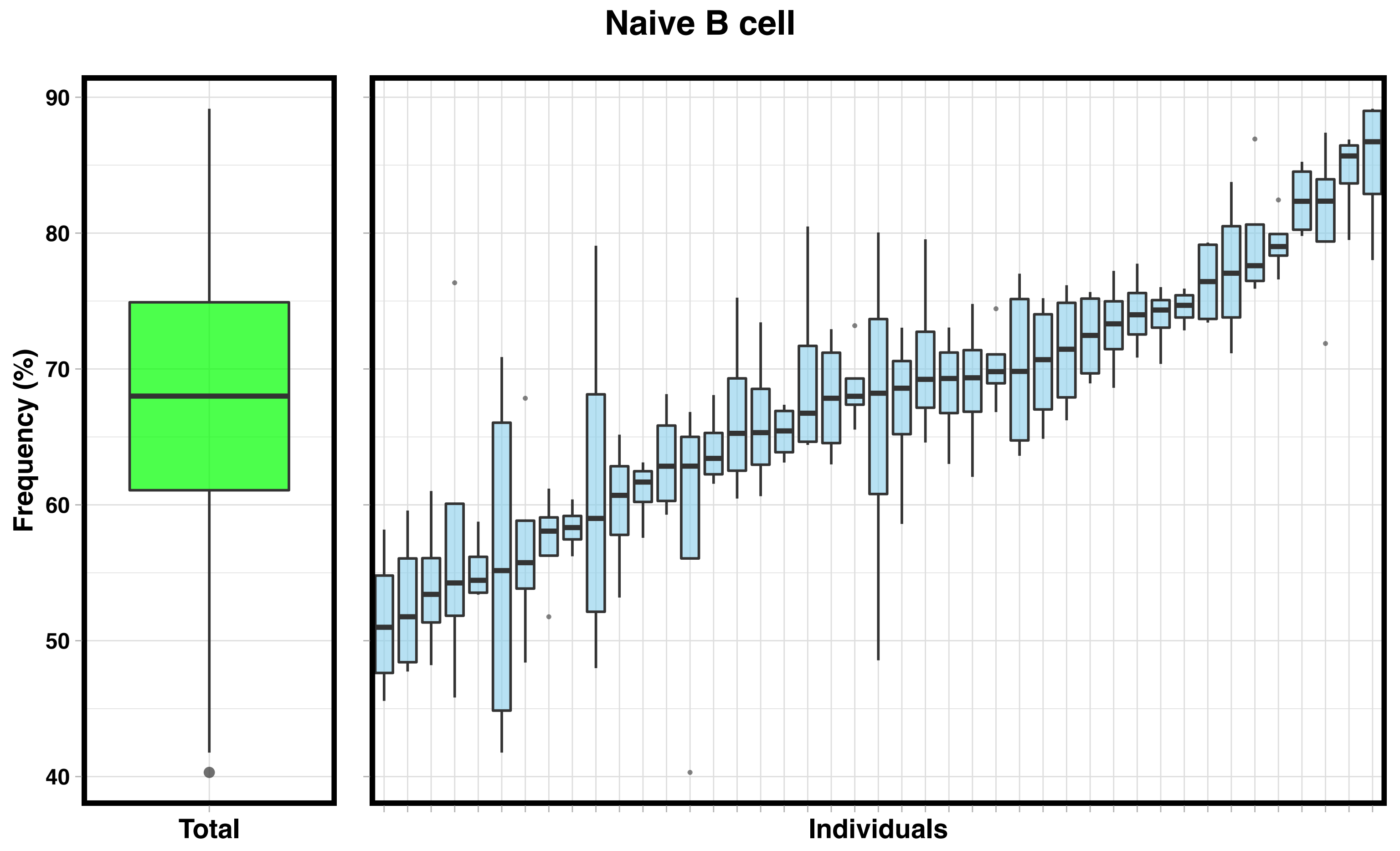

Naive B cell
Frequency (%)
90
80
70
60
50
40
Total
Individuals

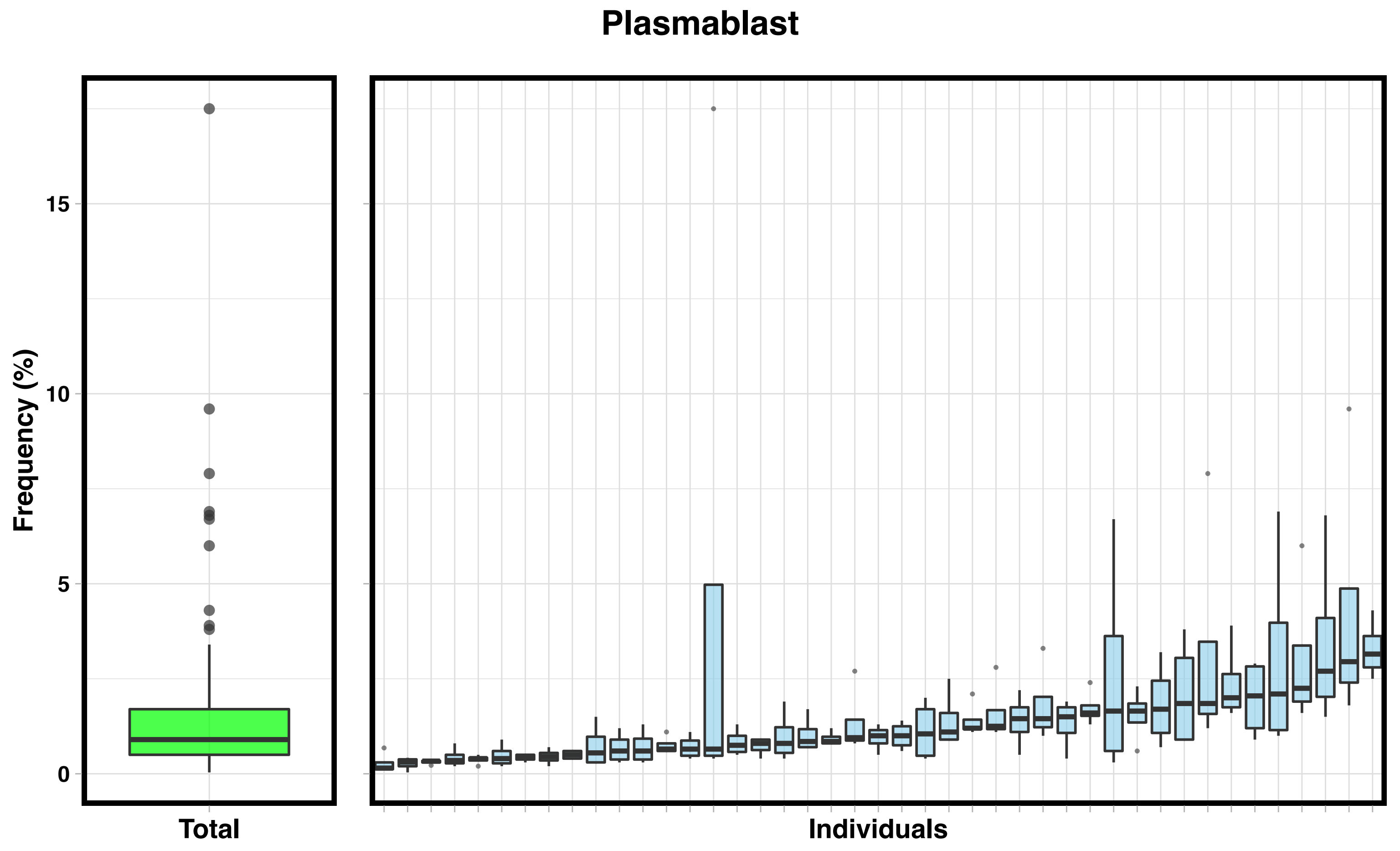
Plasmablast
Frequency (%)
15
10
5
0
Total
Individuals

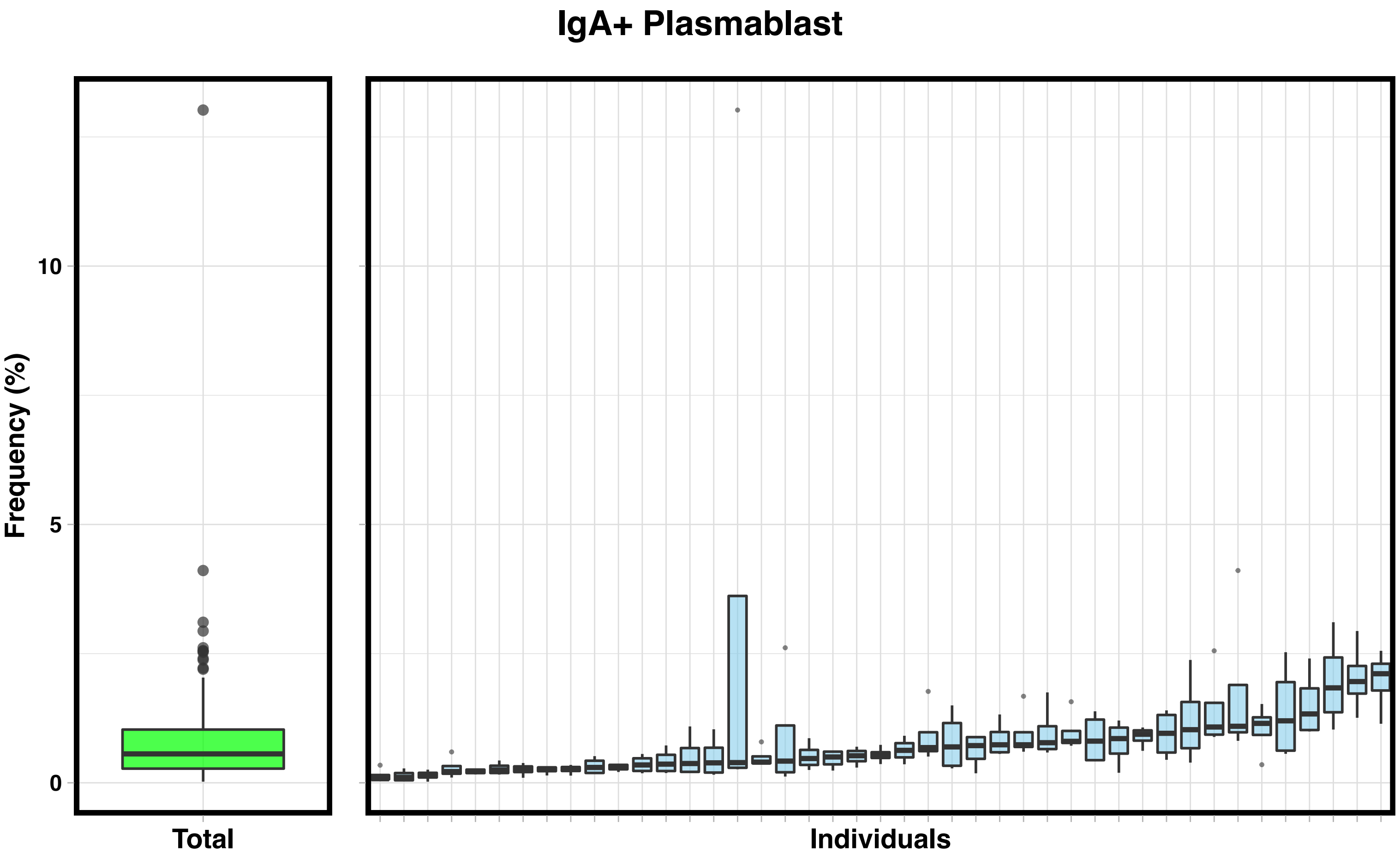
IgA+ Plasmablast
Frequency (%)
10
5
0
Total
Individuals

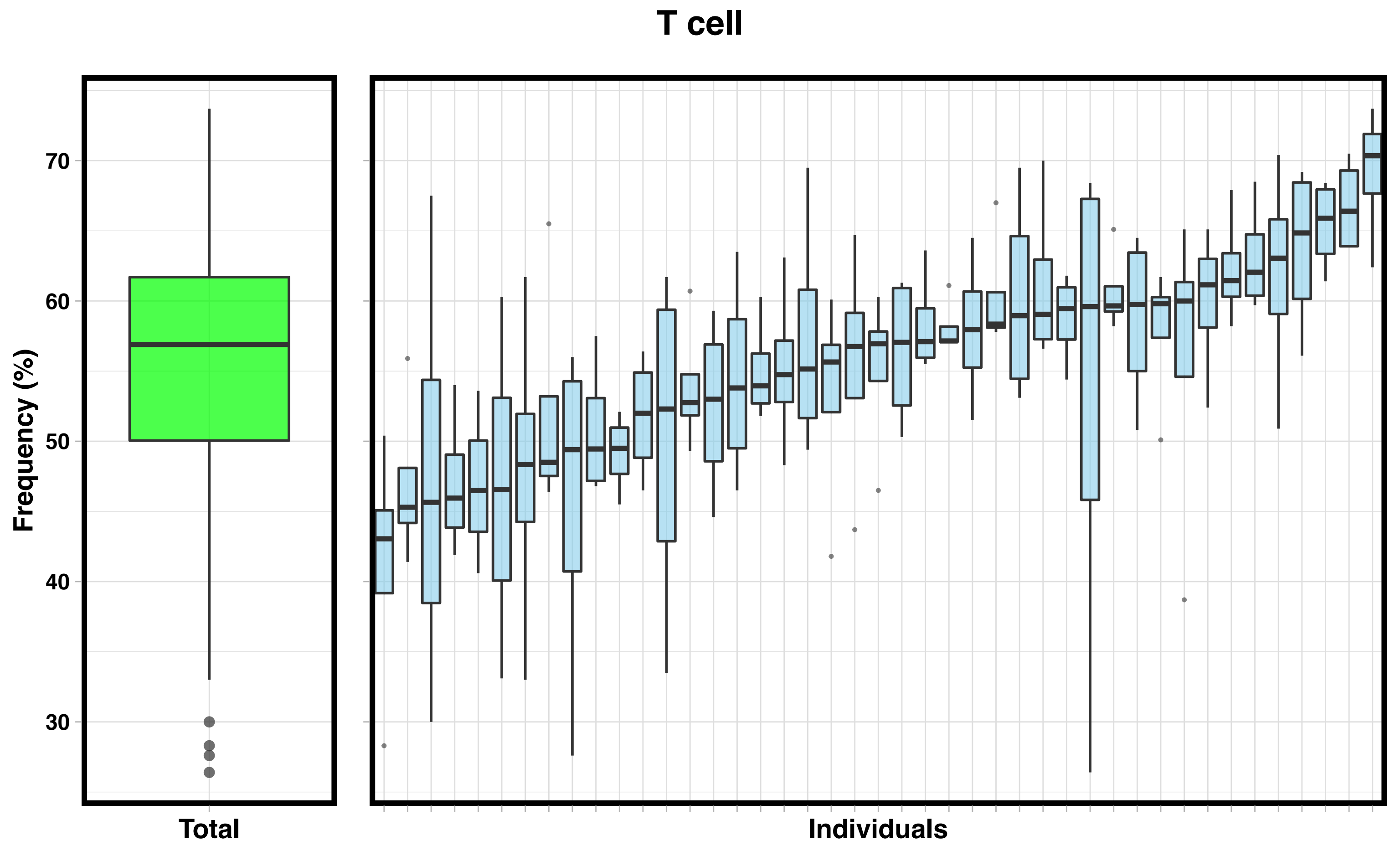

T cell
Frequency (%)
70
60
50
40
30
Total
Individuals

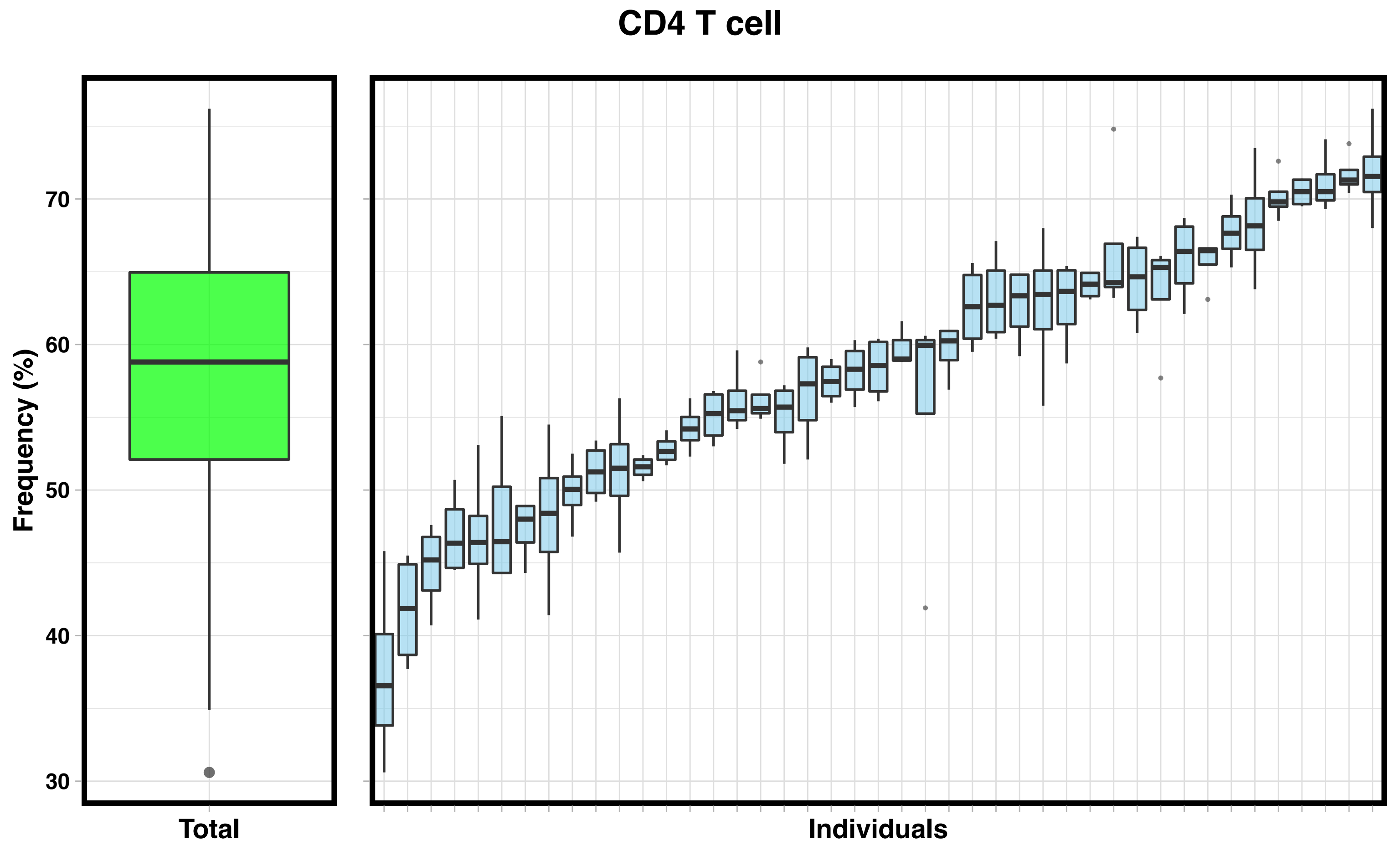

CD4 T cell
Frequency (%)
70
60
50
40
30
Total
Individuals

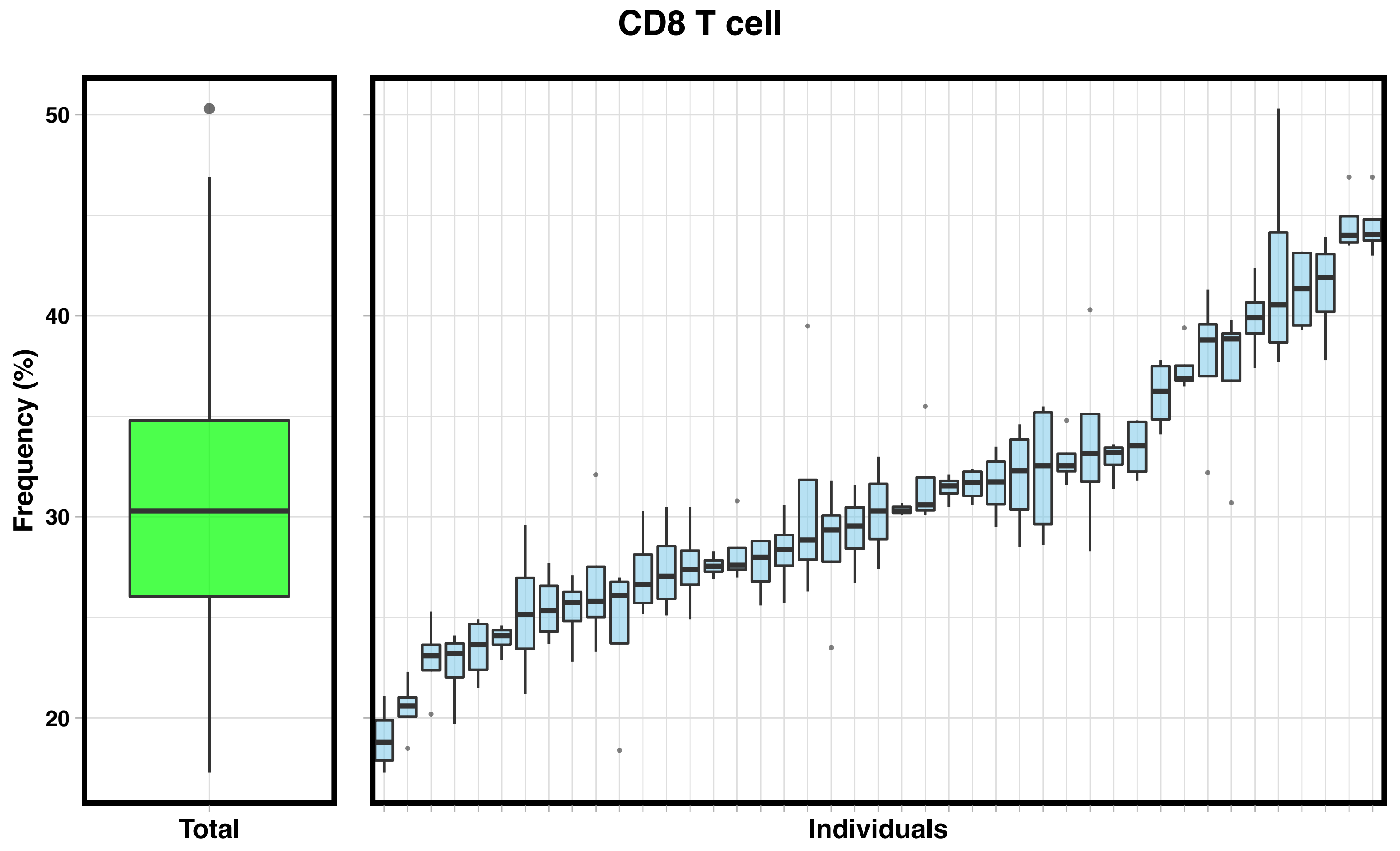
CD8 T cell
Frequency (%)
50
40
30
20
Total
Individuals

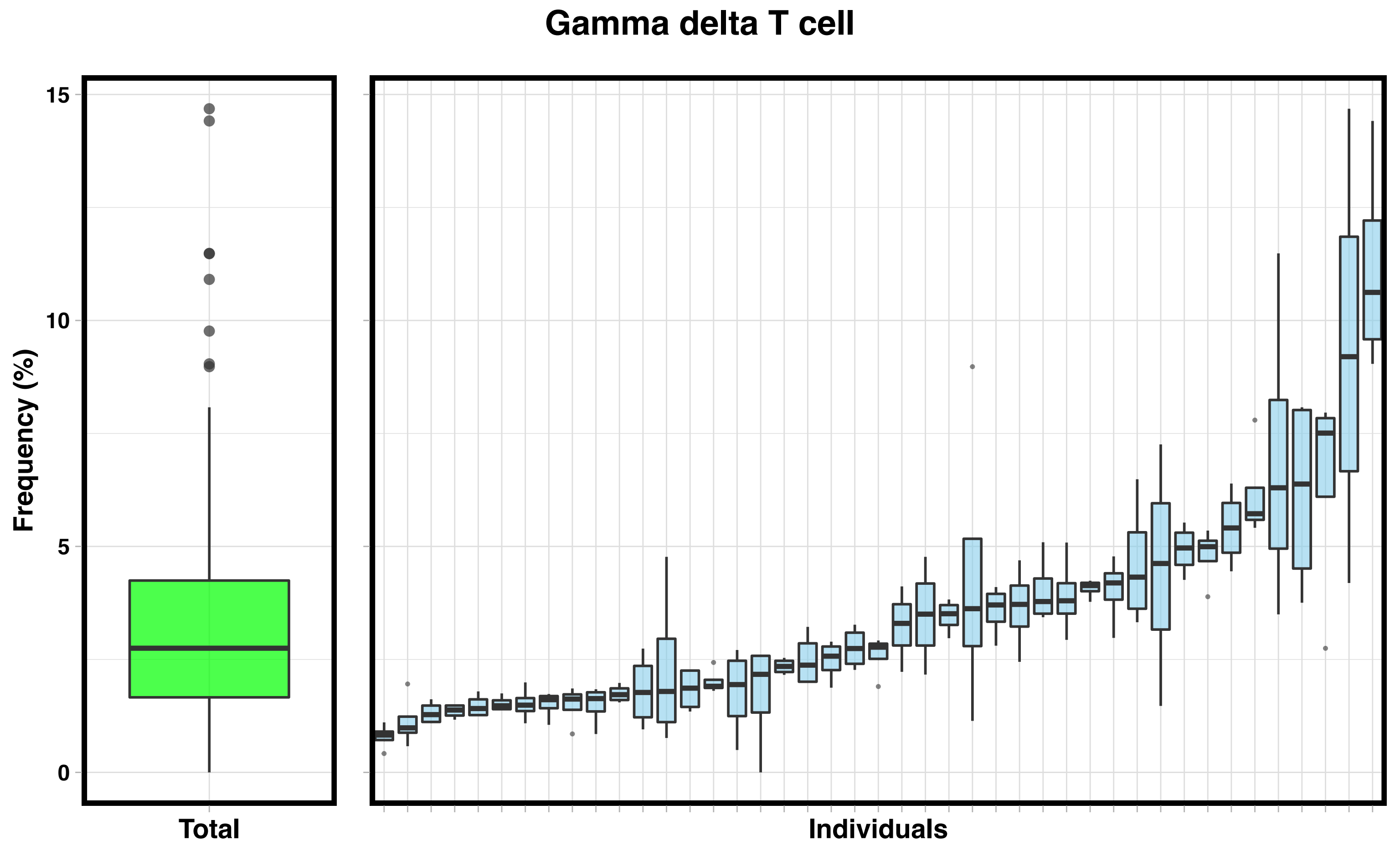
Gamma delta T cell
Frequency (%)
15
10
5
0
Total
Individuals

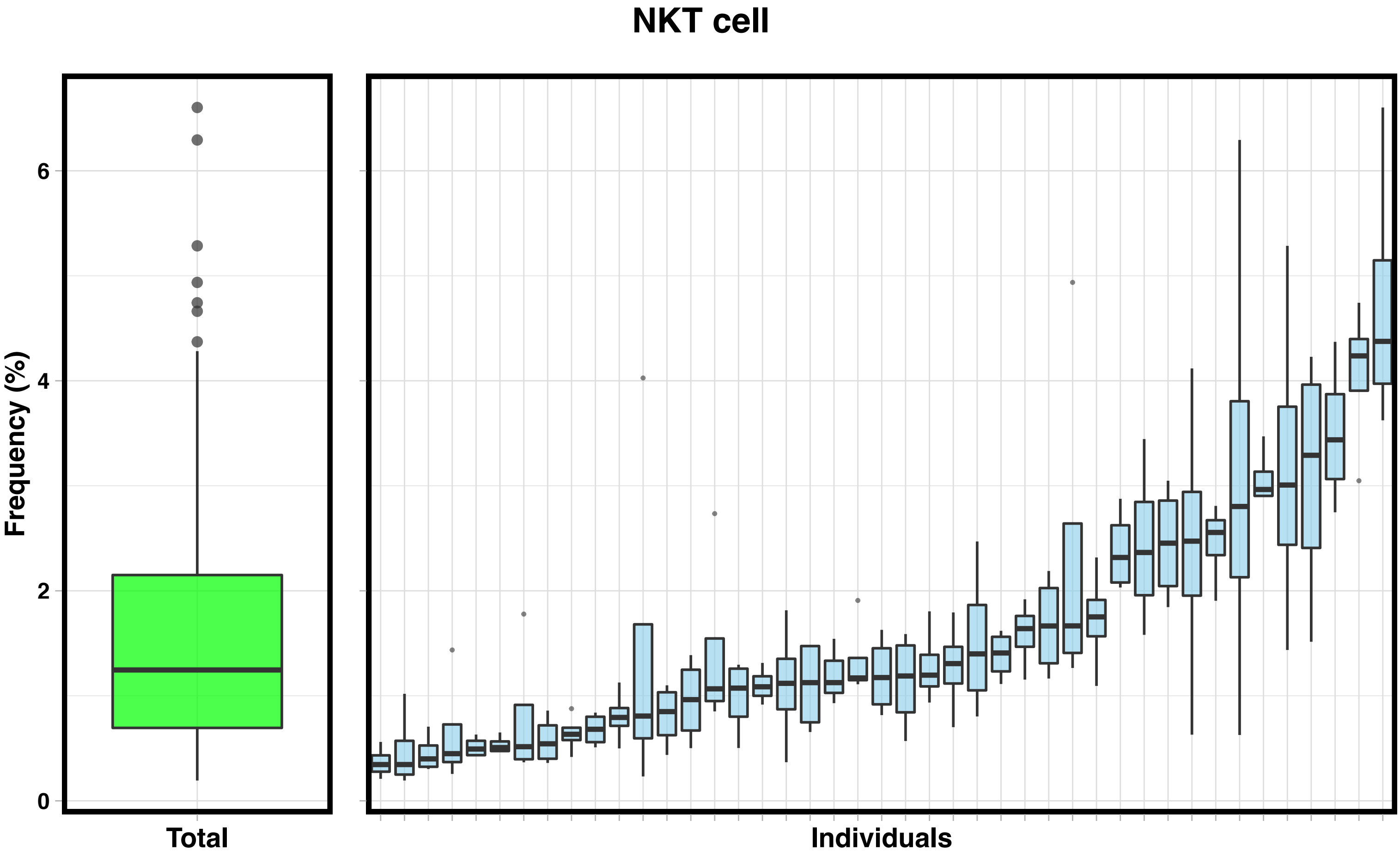
NKT cell
Frequency (%)
0
2
4
6
Total
Individuals

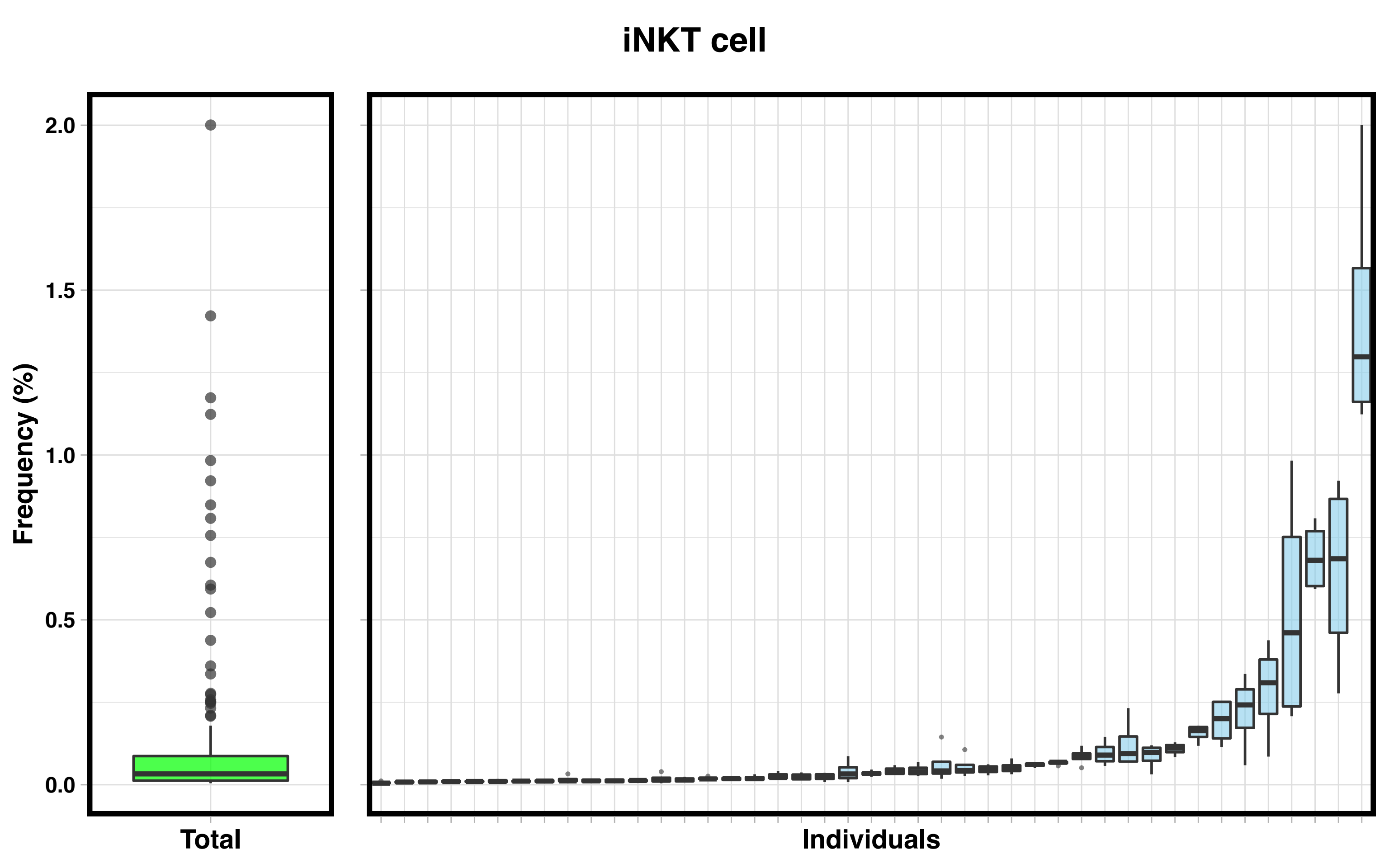

iNKT cell
Frequency (%)
2.0
1.5
1.0
0.5
0.0
Total
Individuals

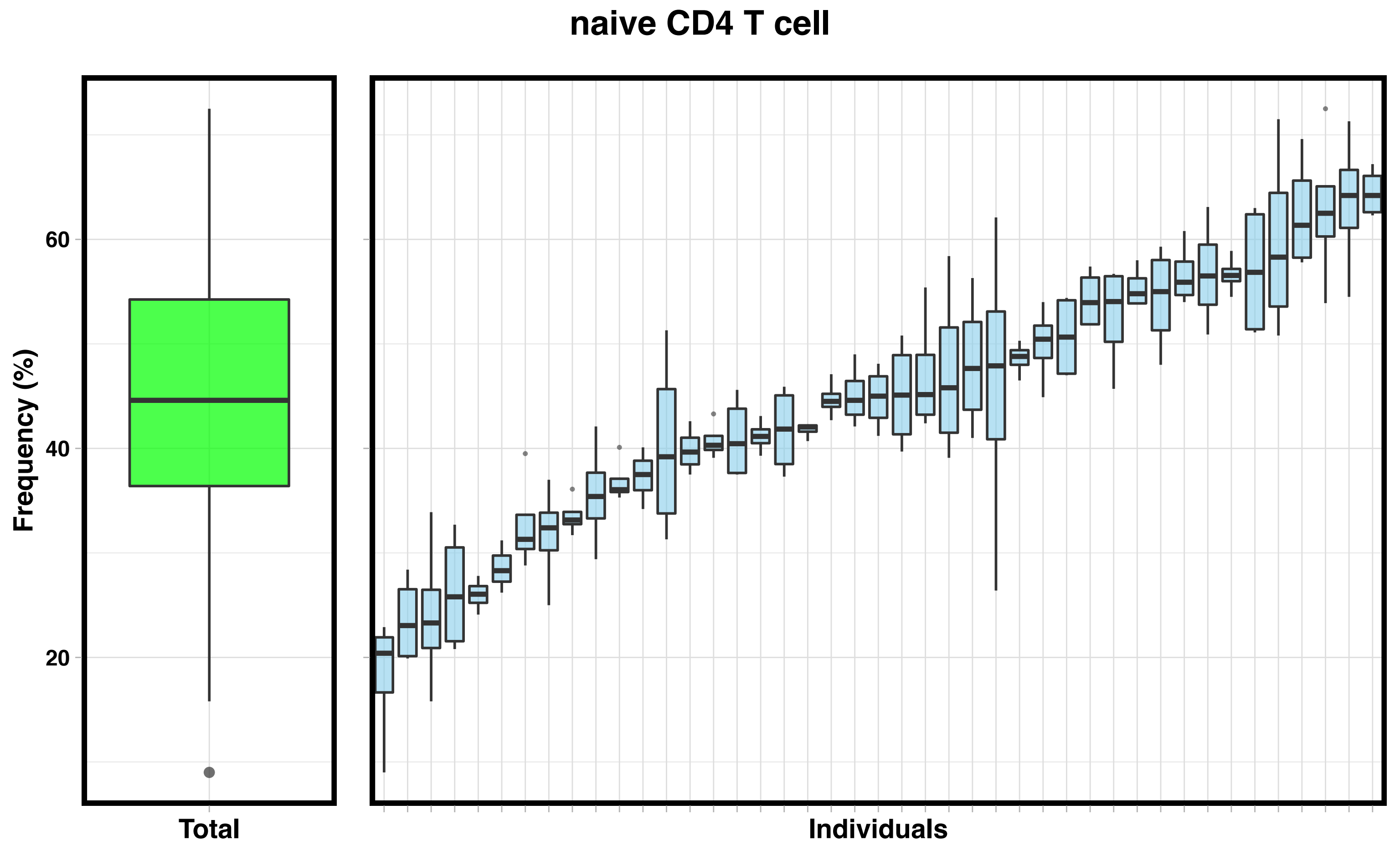
naive CD4 T cell
Frequency (%)
60
40
20
Total
Individuals

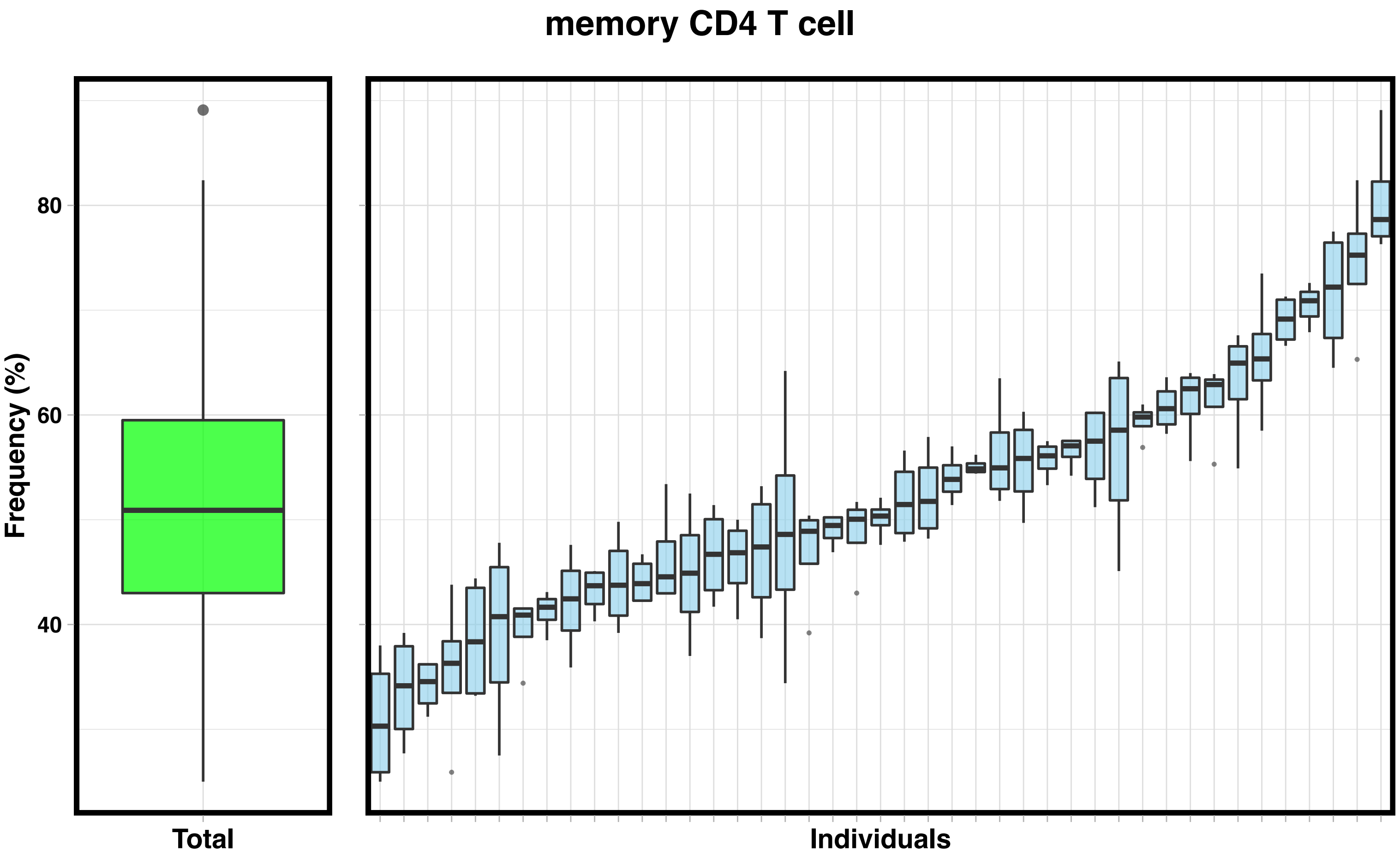

memory CD4 T cell
Frequency (%)
80
60
40
Total
Individuals

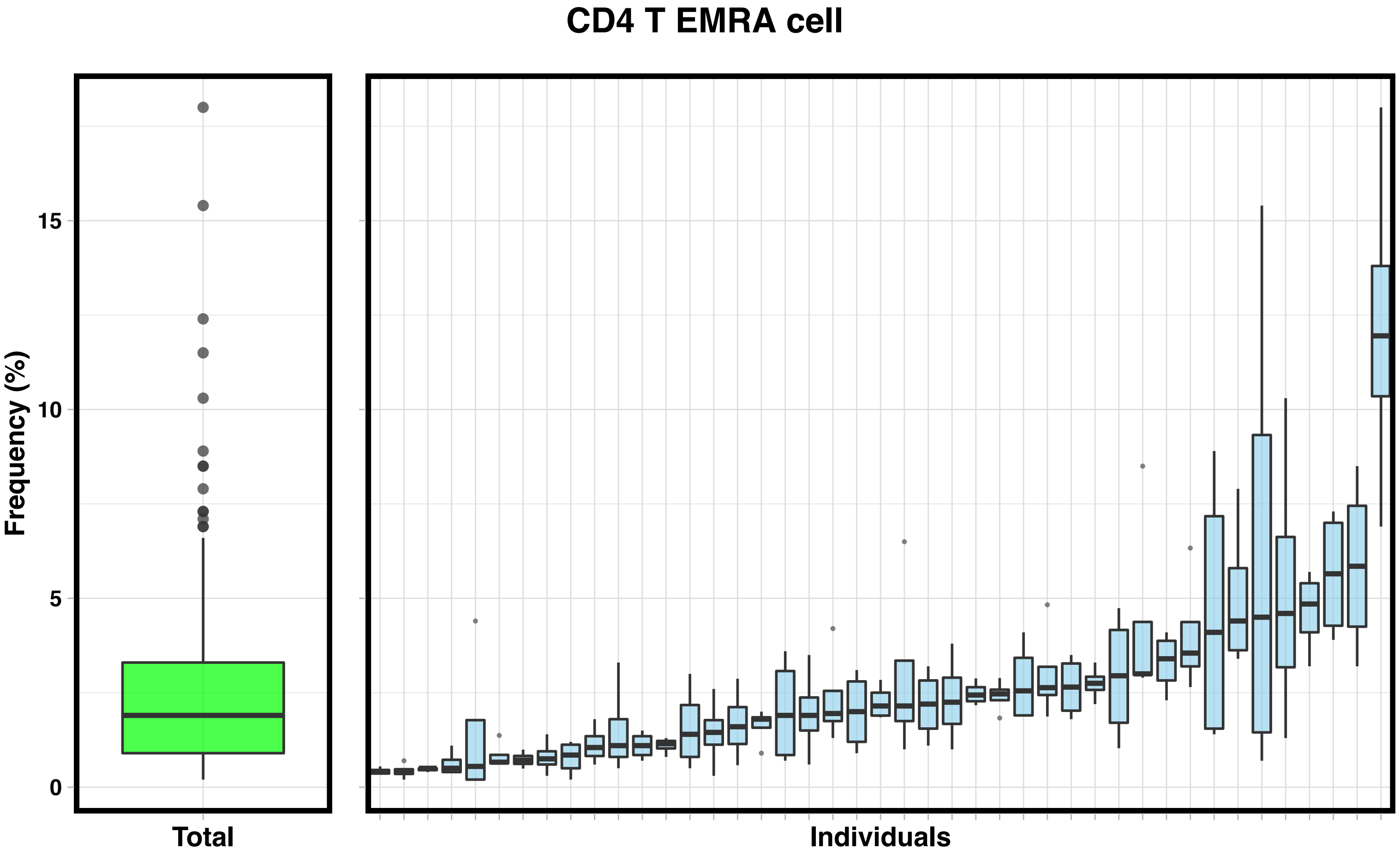

CD4 T EMRA cell
Frequency (%)
0
5
10
15
Total
Individuals

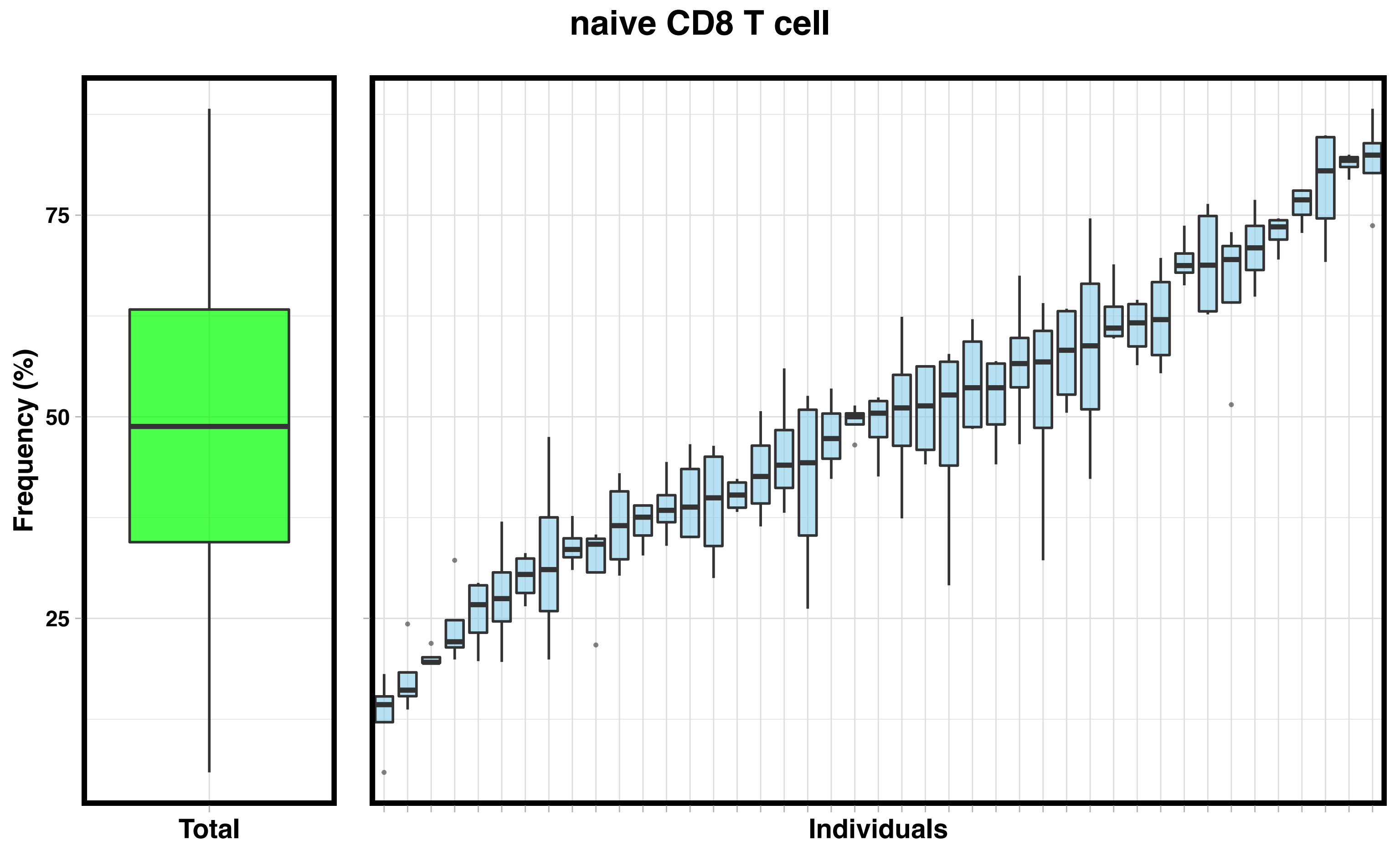
naive CD8 T cell
Frequency (%)
75
50
25
Total
Individuals

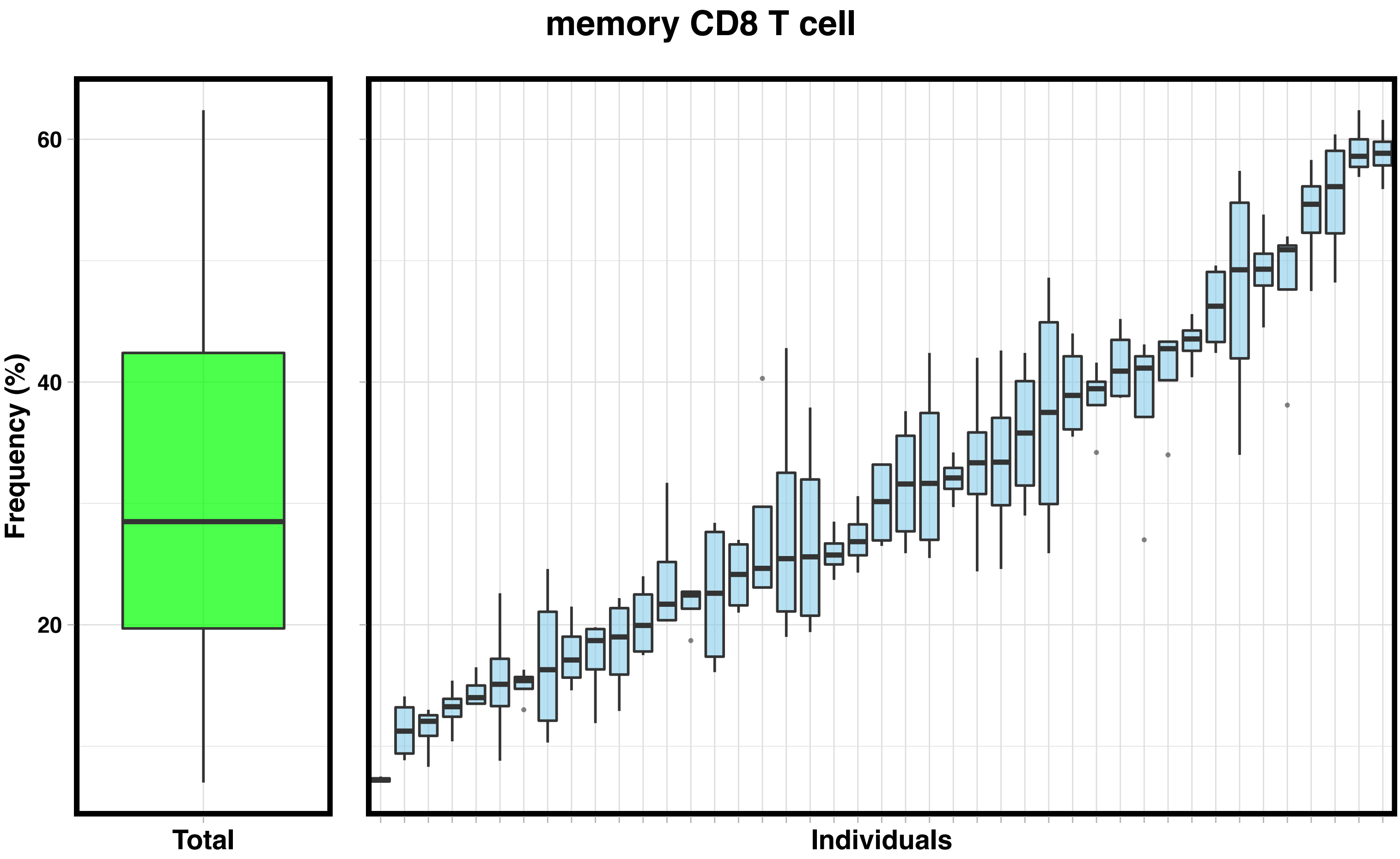
memory CD8 T cell
Frequency (%)
60
40
20
Total
Individuals

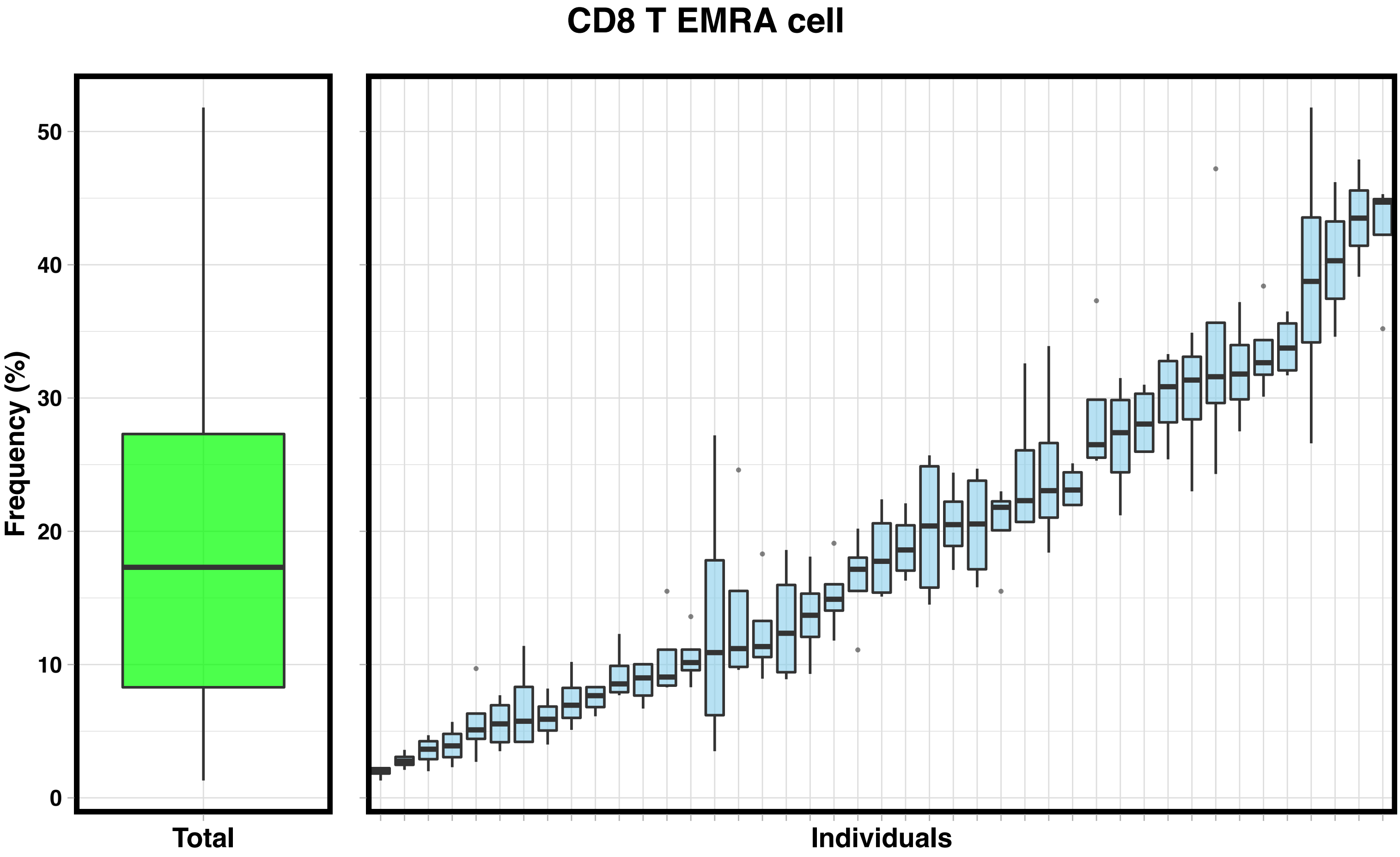
CD8 T EMRA cell
Frequency (%)
0
10
20
30
40
50
Total
Individuals

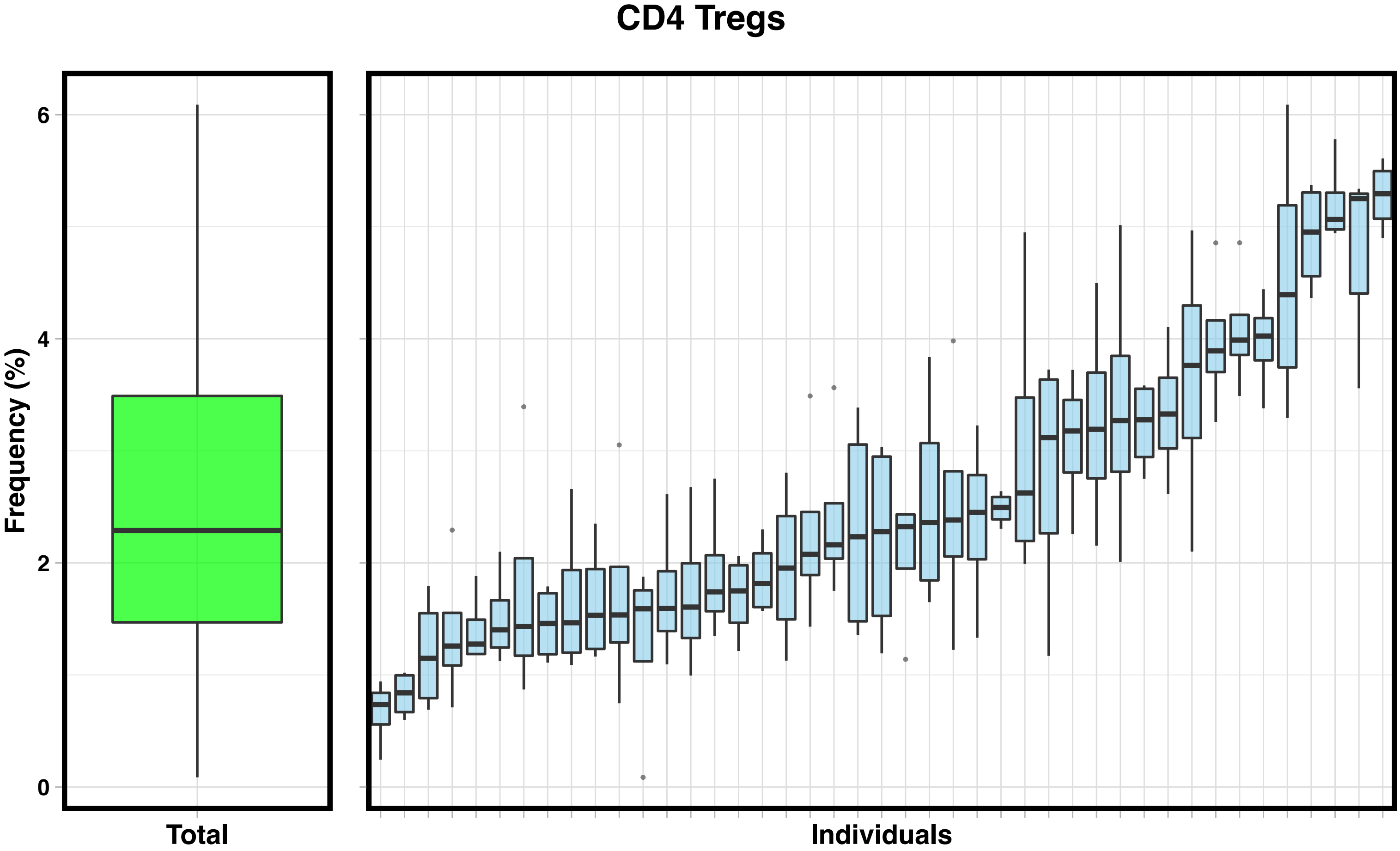
CD4 Tregs
Frequency (%)
6
4
2
0
Total
Individuals

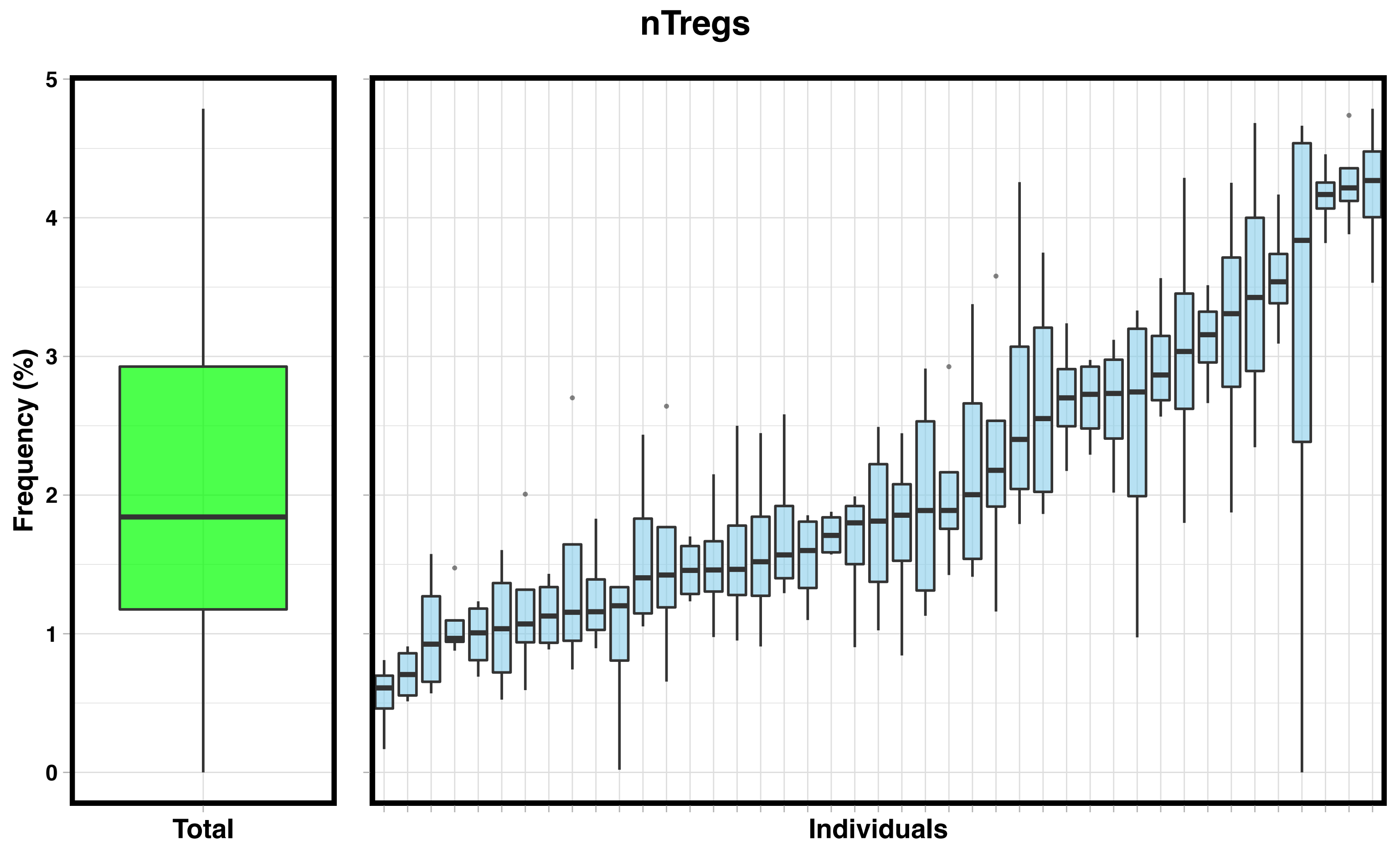
nTregs
Frequency (%)
5
4
3
2
1
0
Total
Individuals

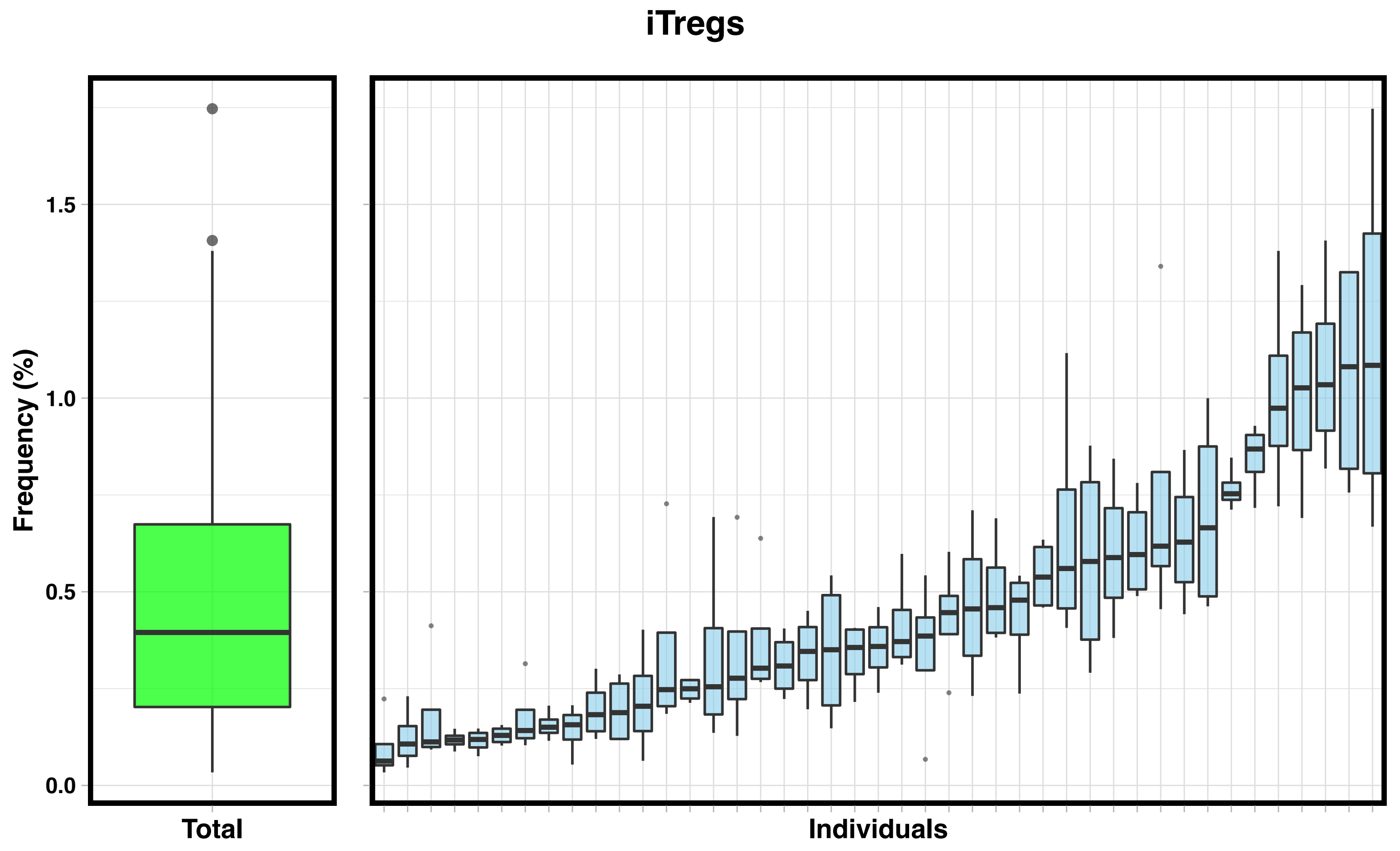

iTregs
Frequency (%)
0.0
0.5
1.0
1.5
Total
Individuals

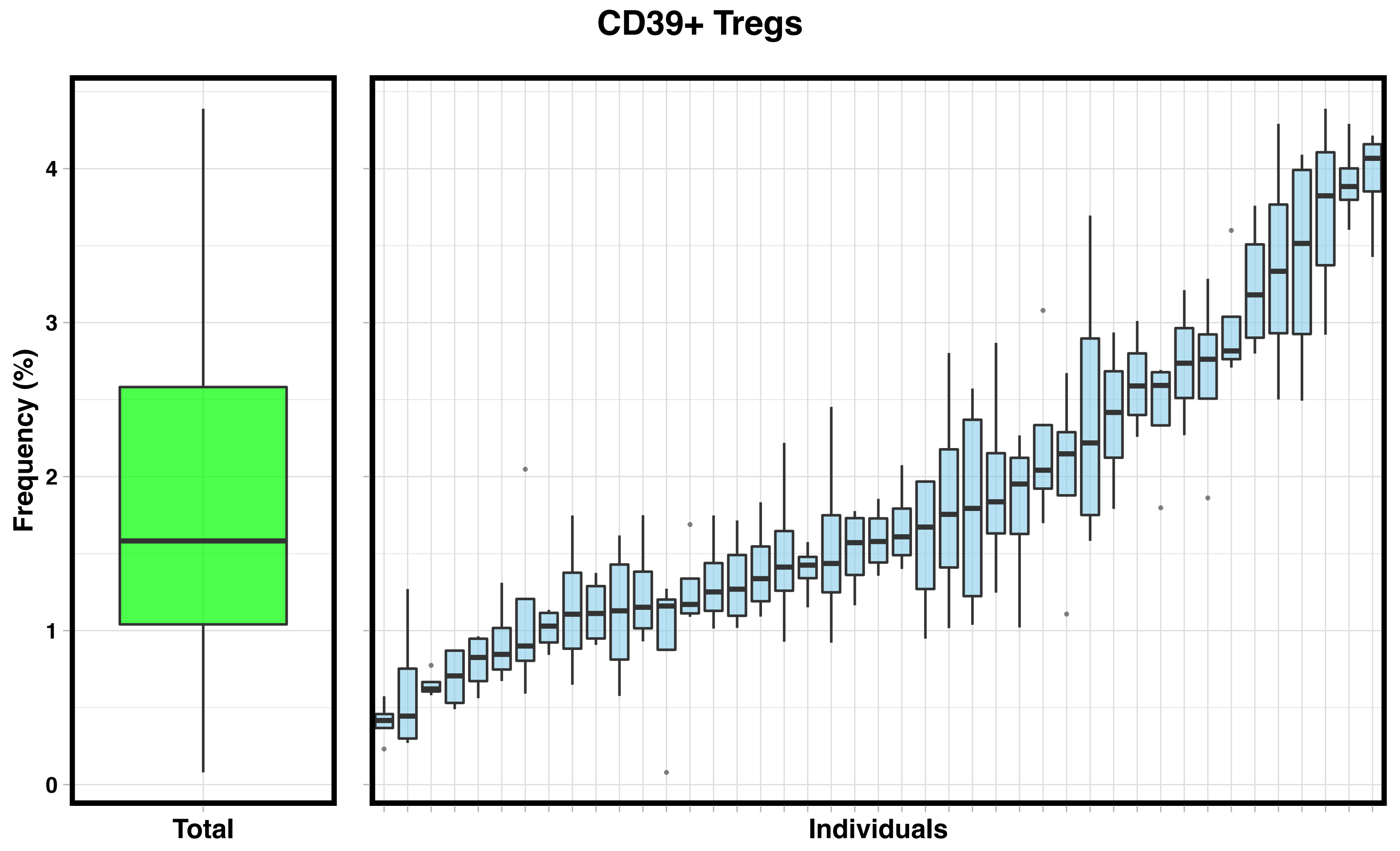

CD39+ Tregs
Frequency (%)
0
1
2
3
4
Total
Individuals

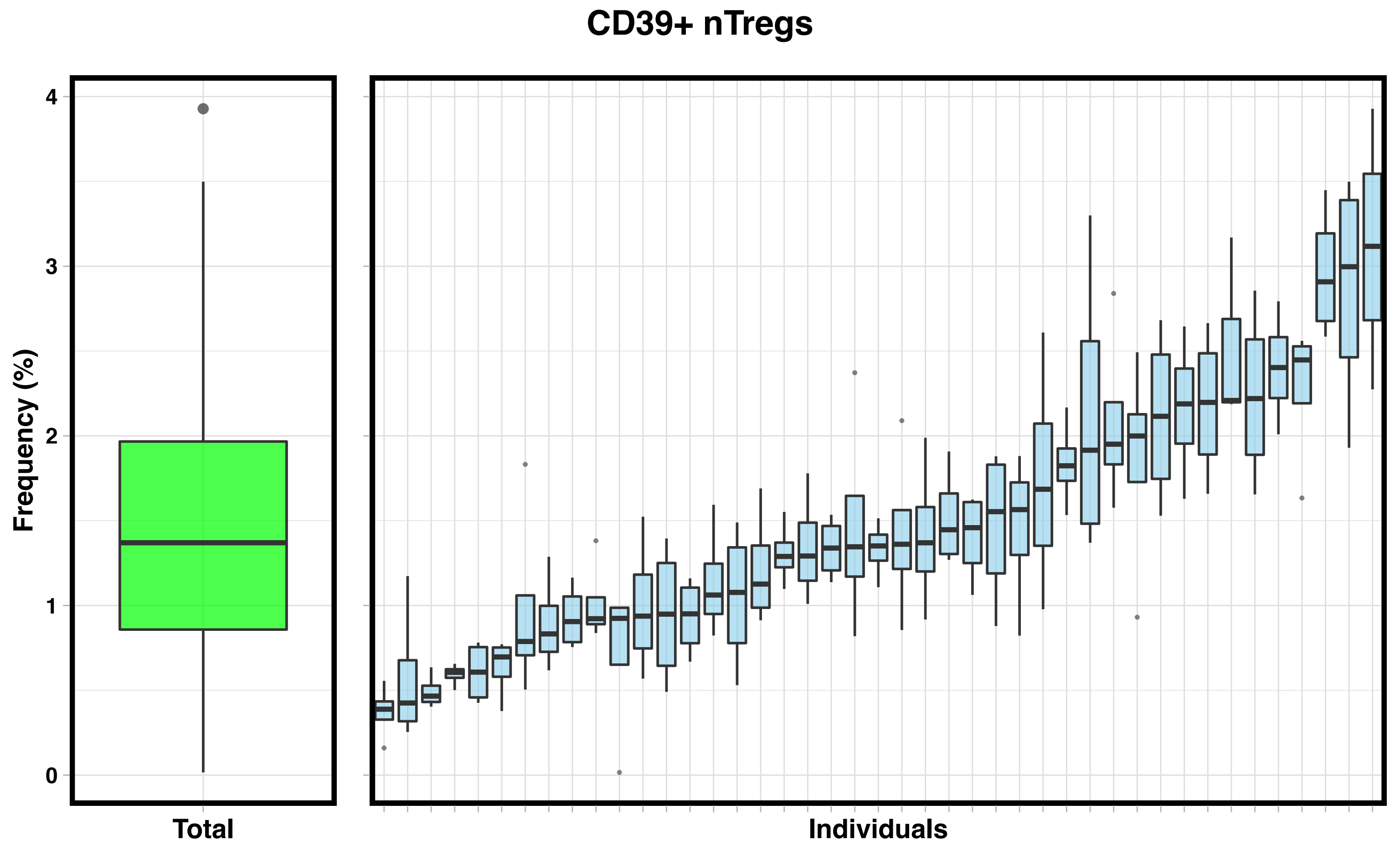
CD39+ nTregs
Frequency (%)
4
3
2
1
0
Total
Individuals

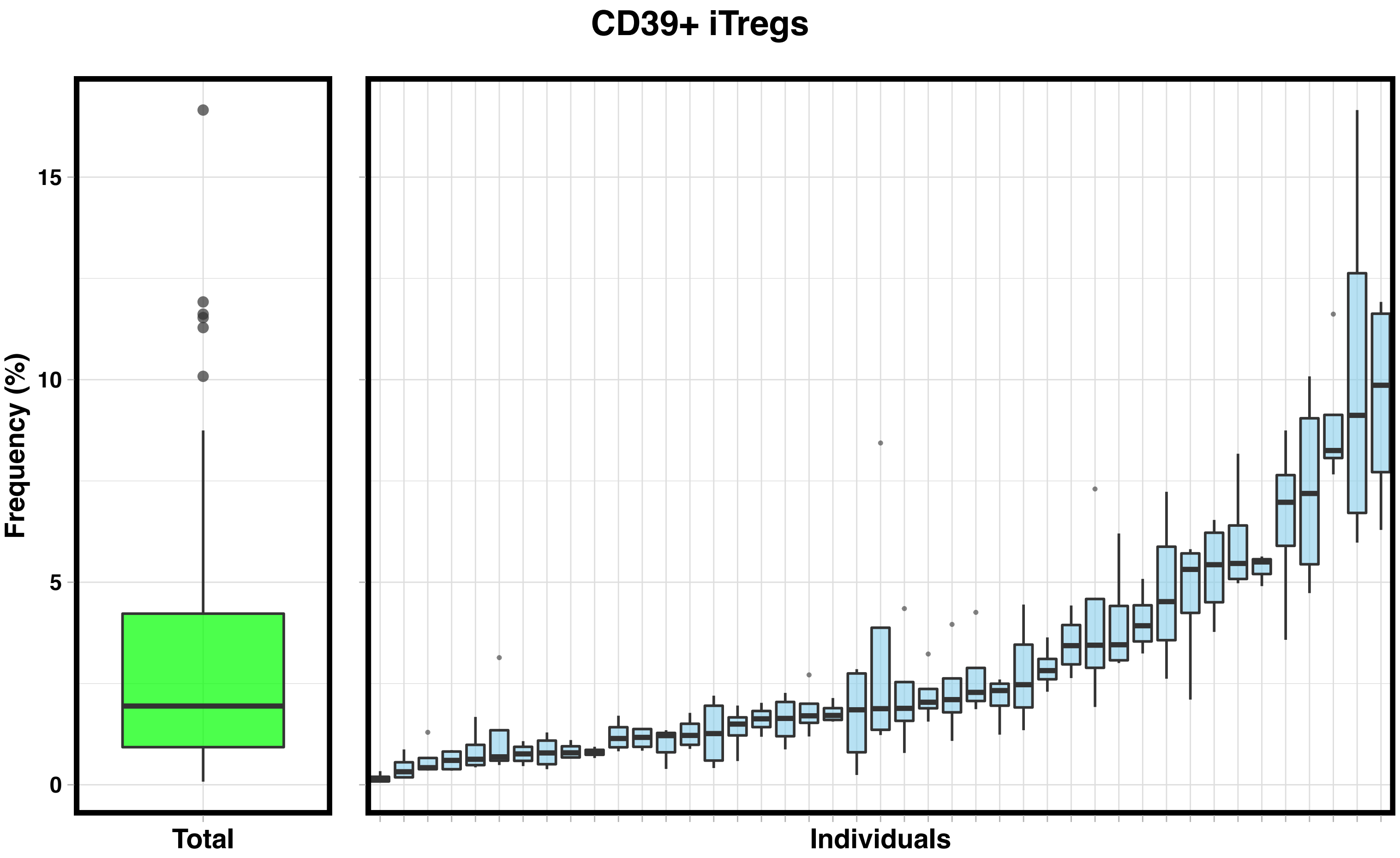

CD39+ iTregs
Frequency (%)
0
5
10
15
Total
Individuals

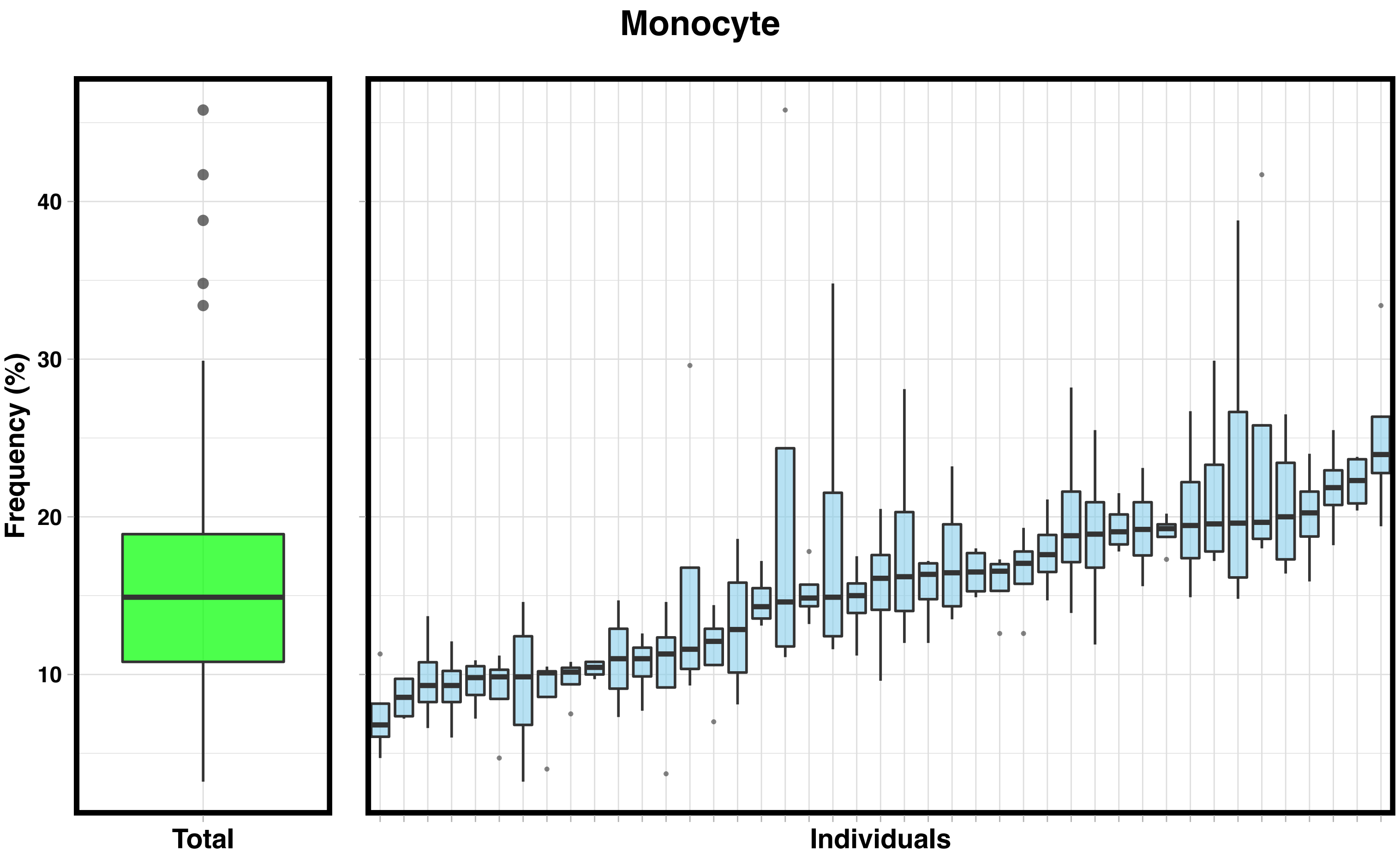
Monocyte
Frequency (%)
40
30
20
10
Total
Individuals

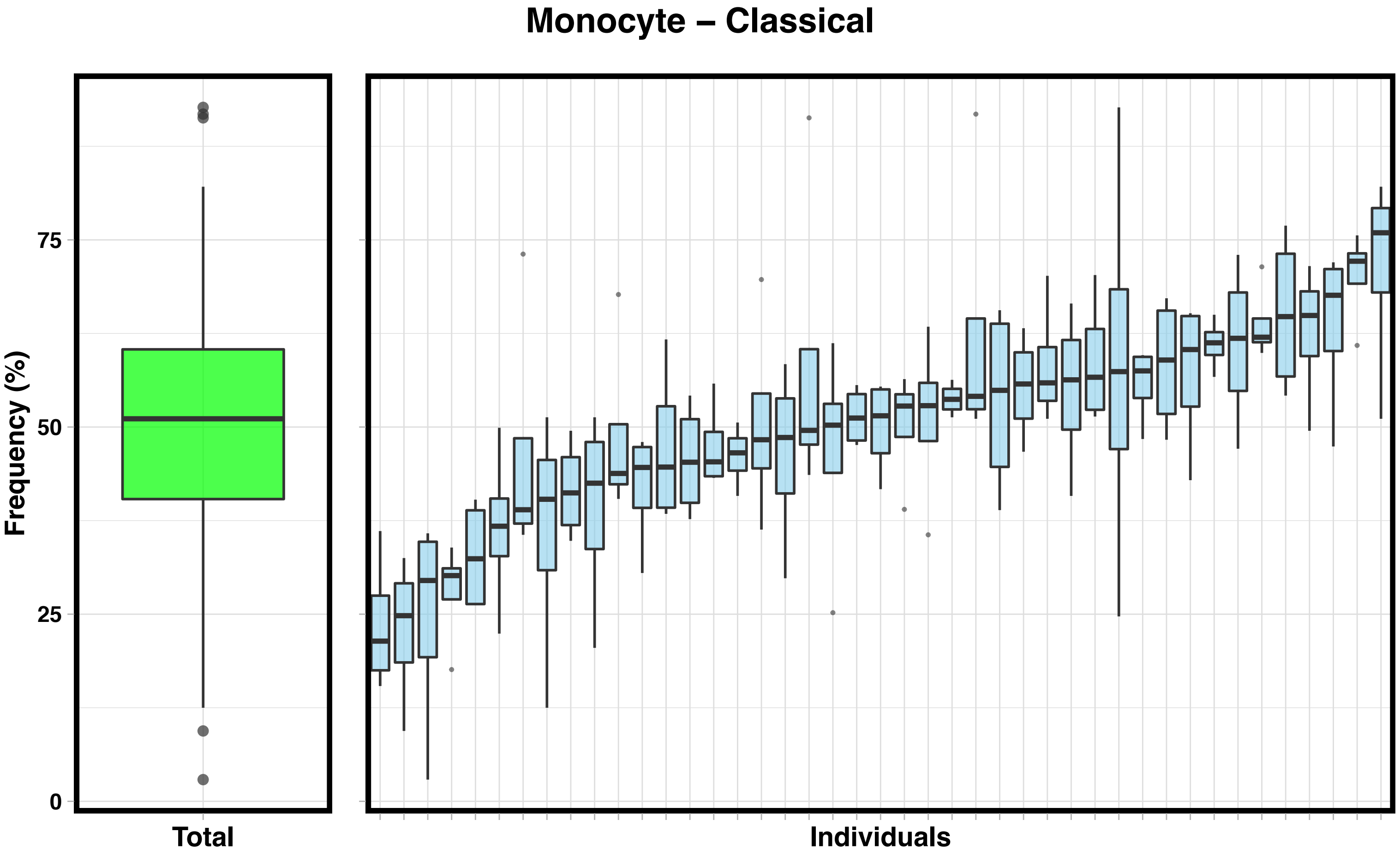

Monocyte – Classical
Frequency (%)
75
50
25
0
Total
Individuals

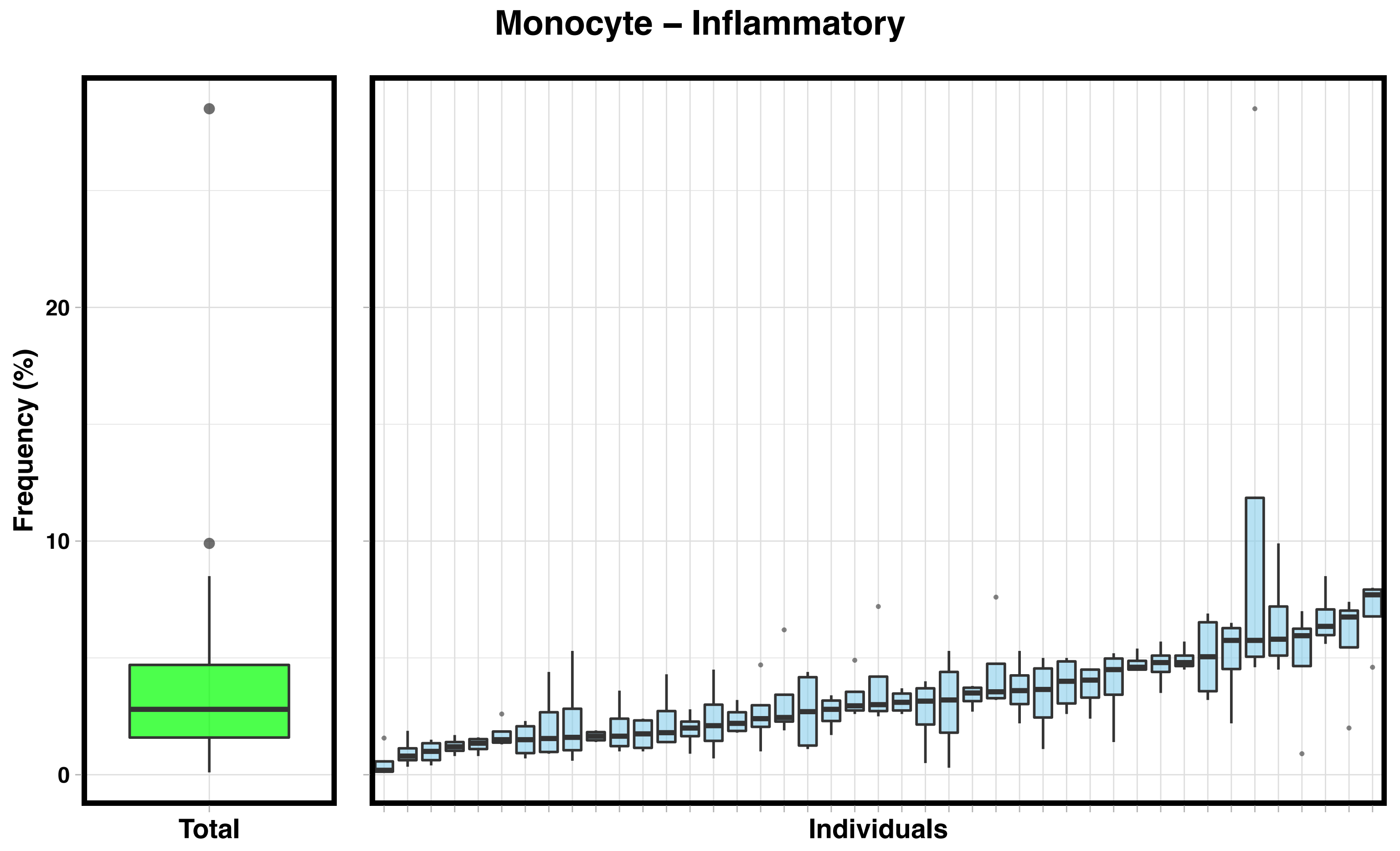

Monocyte – Inflammatory
Frequency (%)
20
10
0
Total
Individuals

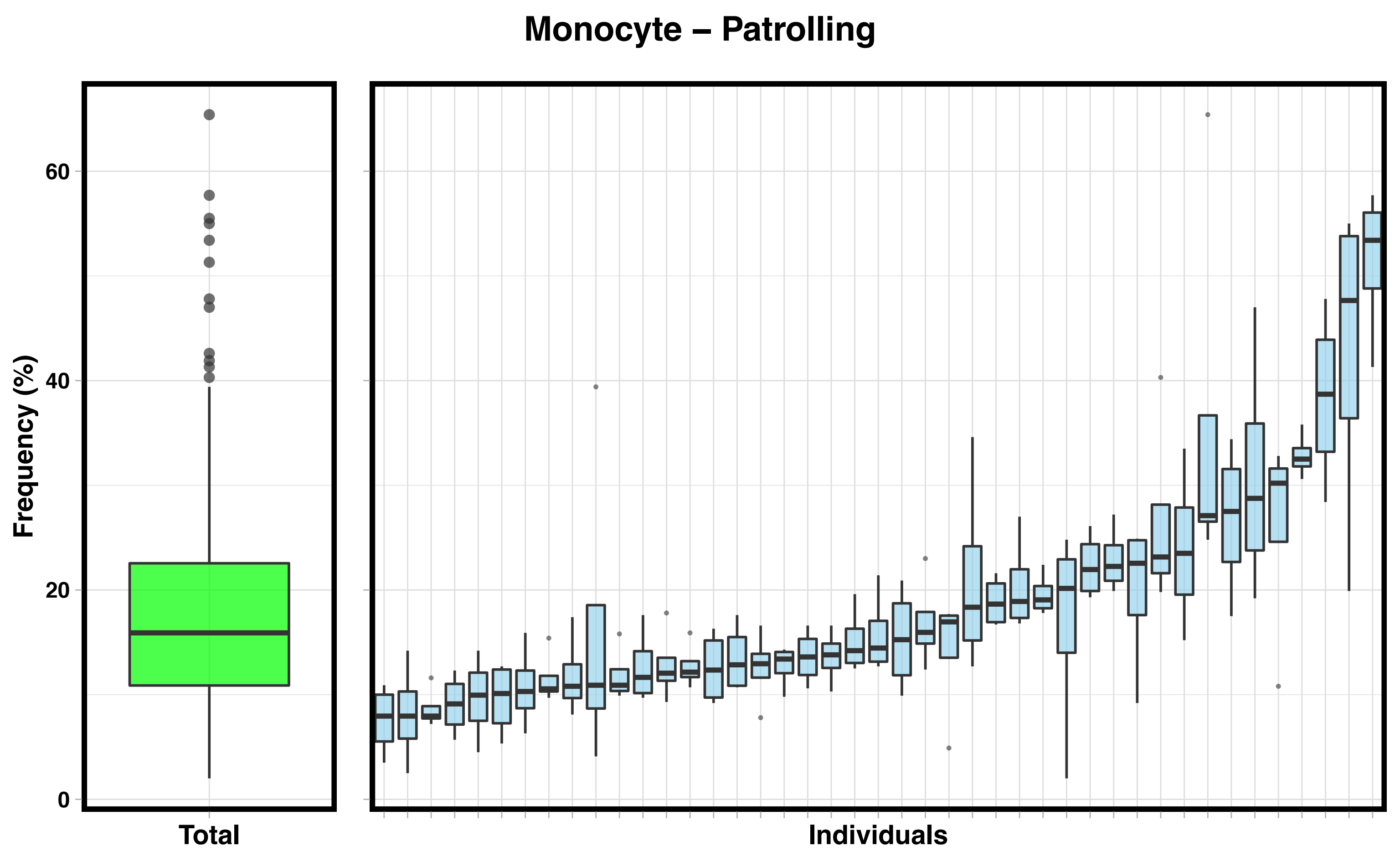
Monocyte – Patrolling
Frequency (%)
0
20
40
60
Total
Individuals

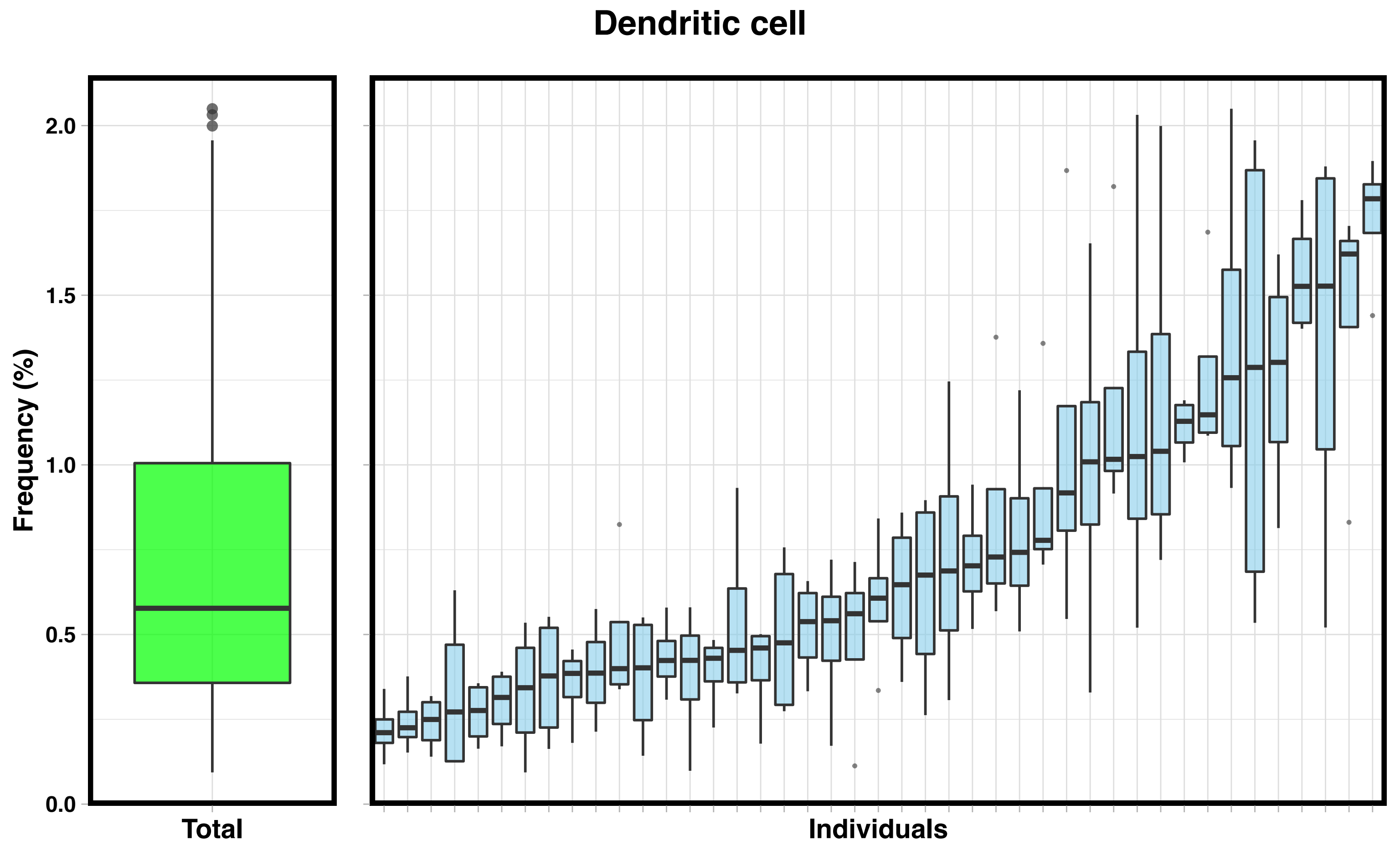

Dendritic cell
Frequency (%)
2.0
1.5
1.0
0.5
0.0
Total
Individuals

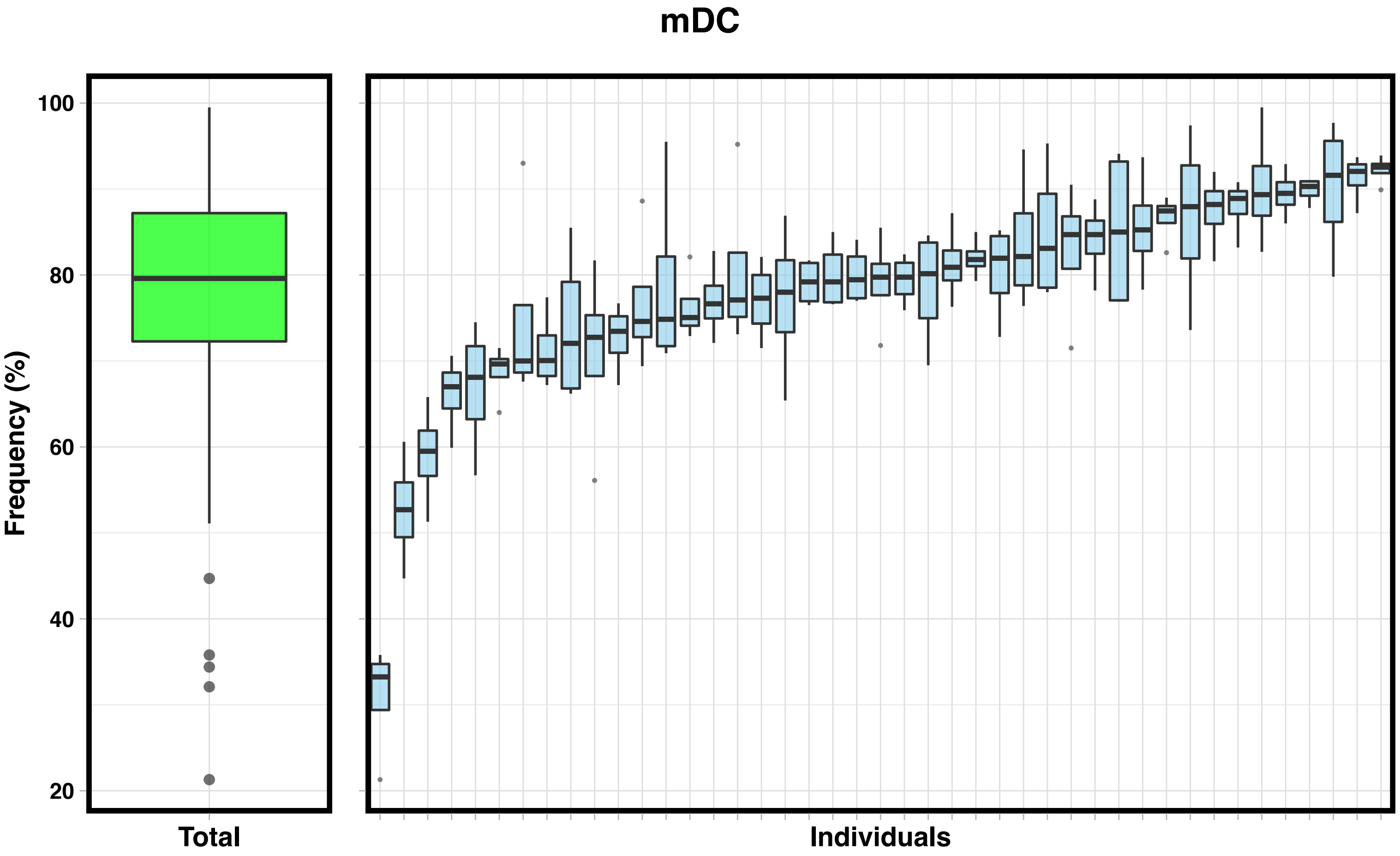

mDC
Frequency (%)
100
80
60
40
20
Total
Individuals

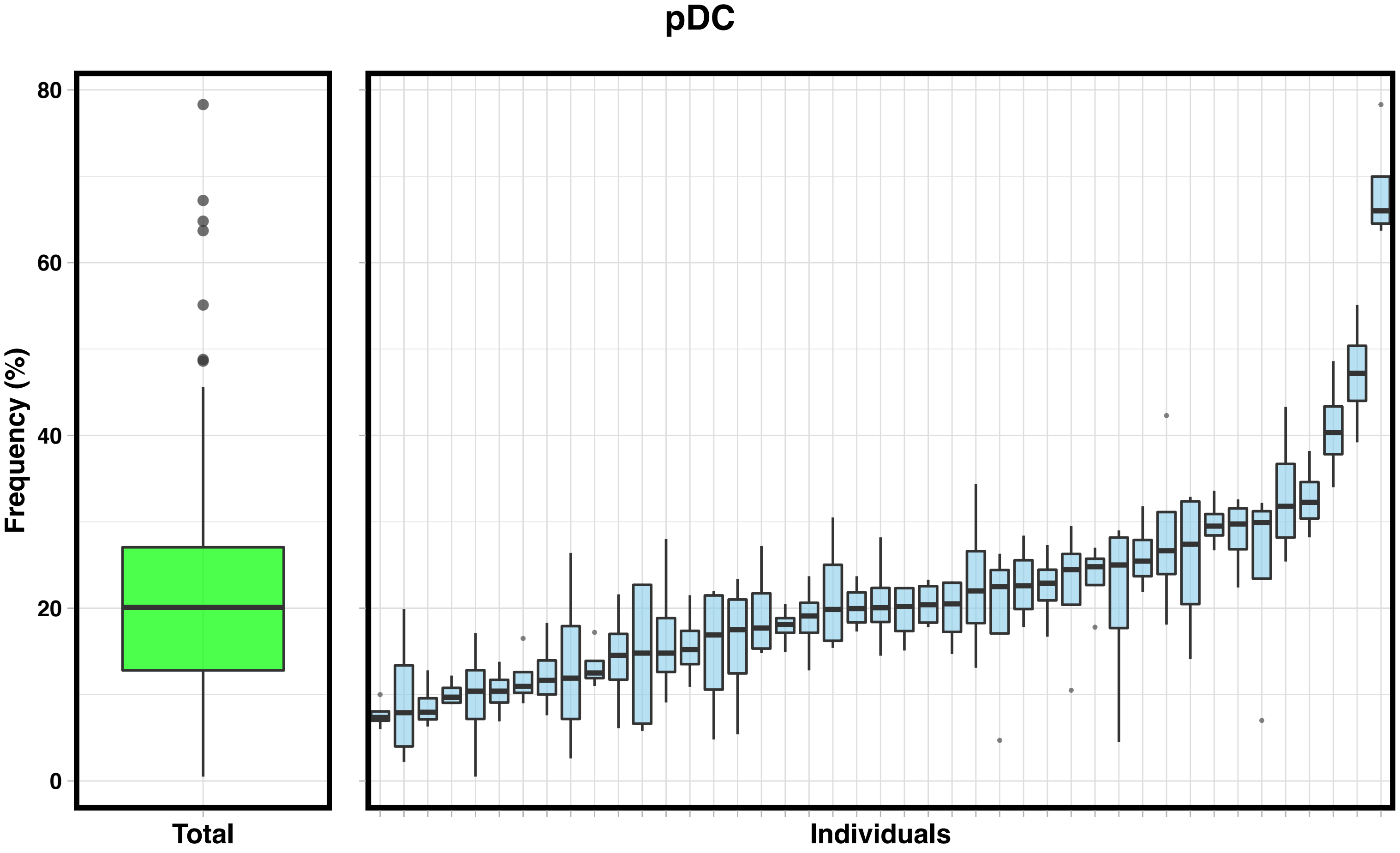
pDC
Frequency (%)
80
60
40
20
0
Total
Individuals

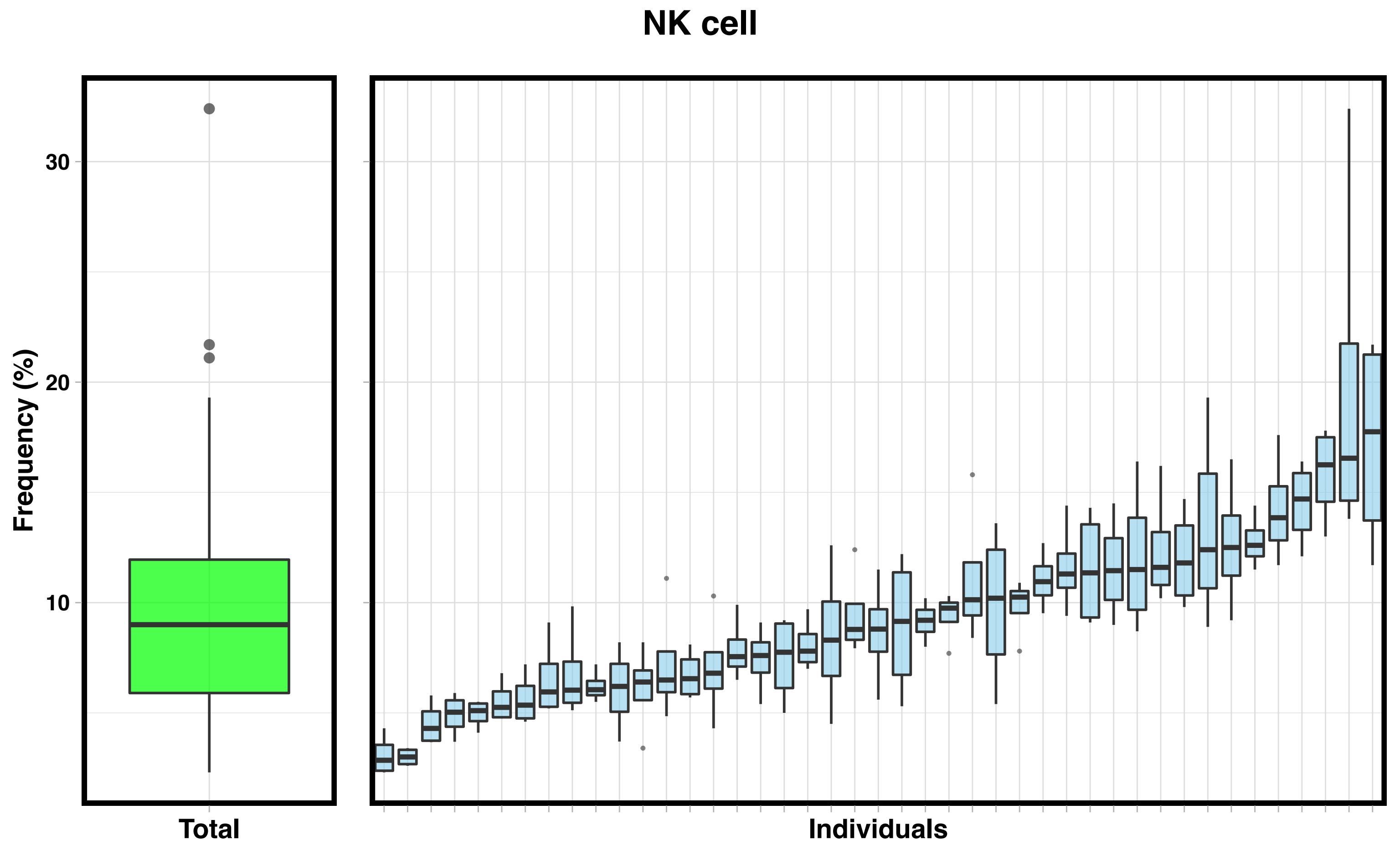
NK cell
Frequency (%)
30
20
10
Total
Individuals

SUPPLEMENTARY FIGURES

Characterization of biological variation of peripheral blood immune cytome in an Indian cohort

Parna Kanodia, Gurvinder Kaur, Poonam Coshic, Kabita Chatterjee, Teresa Neeman, Anna George, Satyajit Rath, Vineeta Bal, Savit B Prabhu.

Supplementary Figure 5

Comparison of within-individual variance with between-individual variance. Histograms show the null distribution of differences between random groups of individuals. Observed value of between-individual difference minus within-individual difference is shown as vertical line. P-values are shown in Supplementary Table 6.

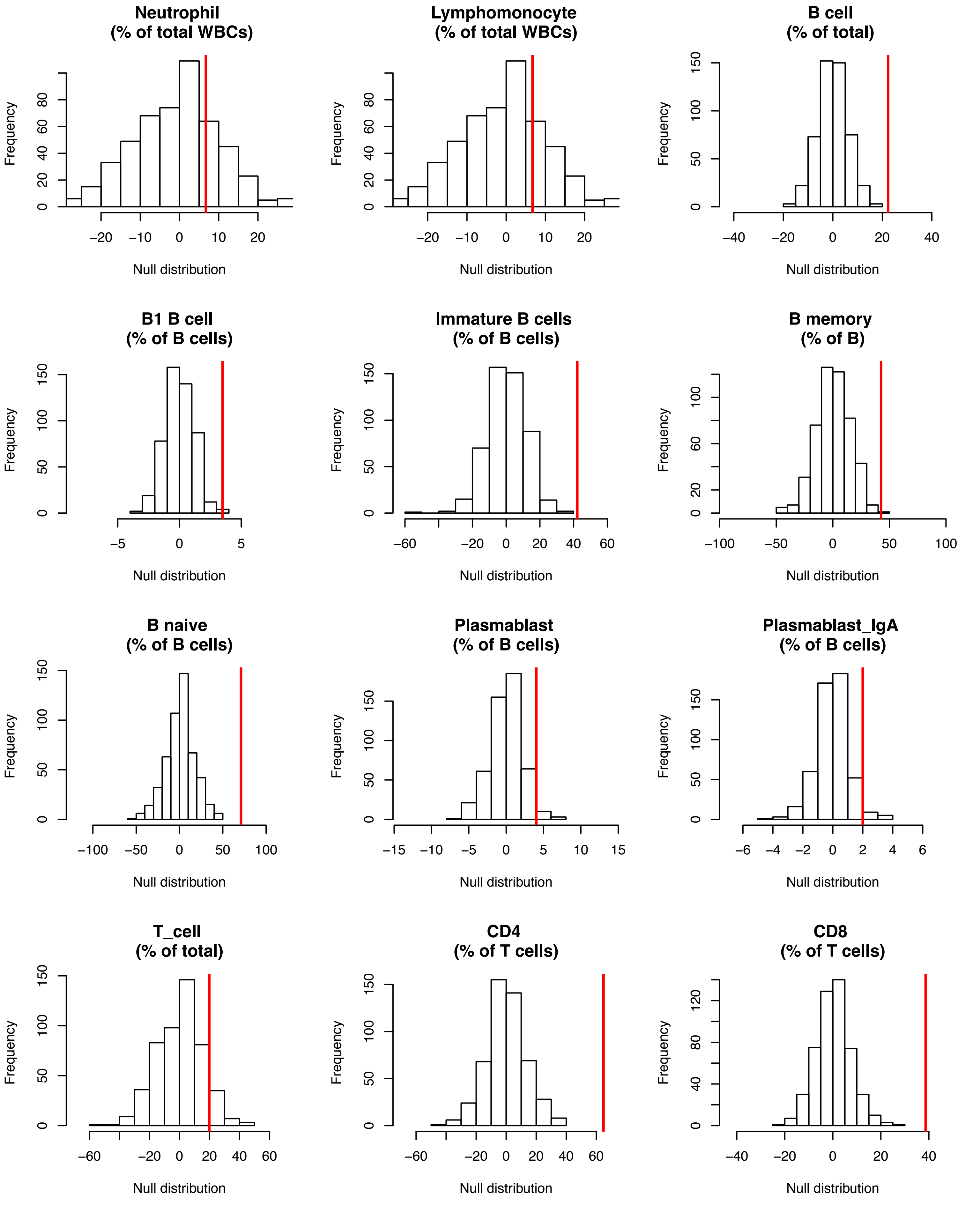

Neutrophil
(% of total WBCs)
Lymphomonocyte
(% of total WBCs)
B cell
(% of total)
B1 B cell
(% of B cells)
Immature B cells
(% of B cells)
B memory
(% of B)
B naive
(% of B cells)
Plasmablast
(% of B cells)
Plasmablast_IgA
(% of B cells)
T_cell
(% of total)
CD4
(% of T cells)
CD8
(% of T cells)
Frequency
Null distribution

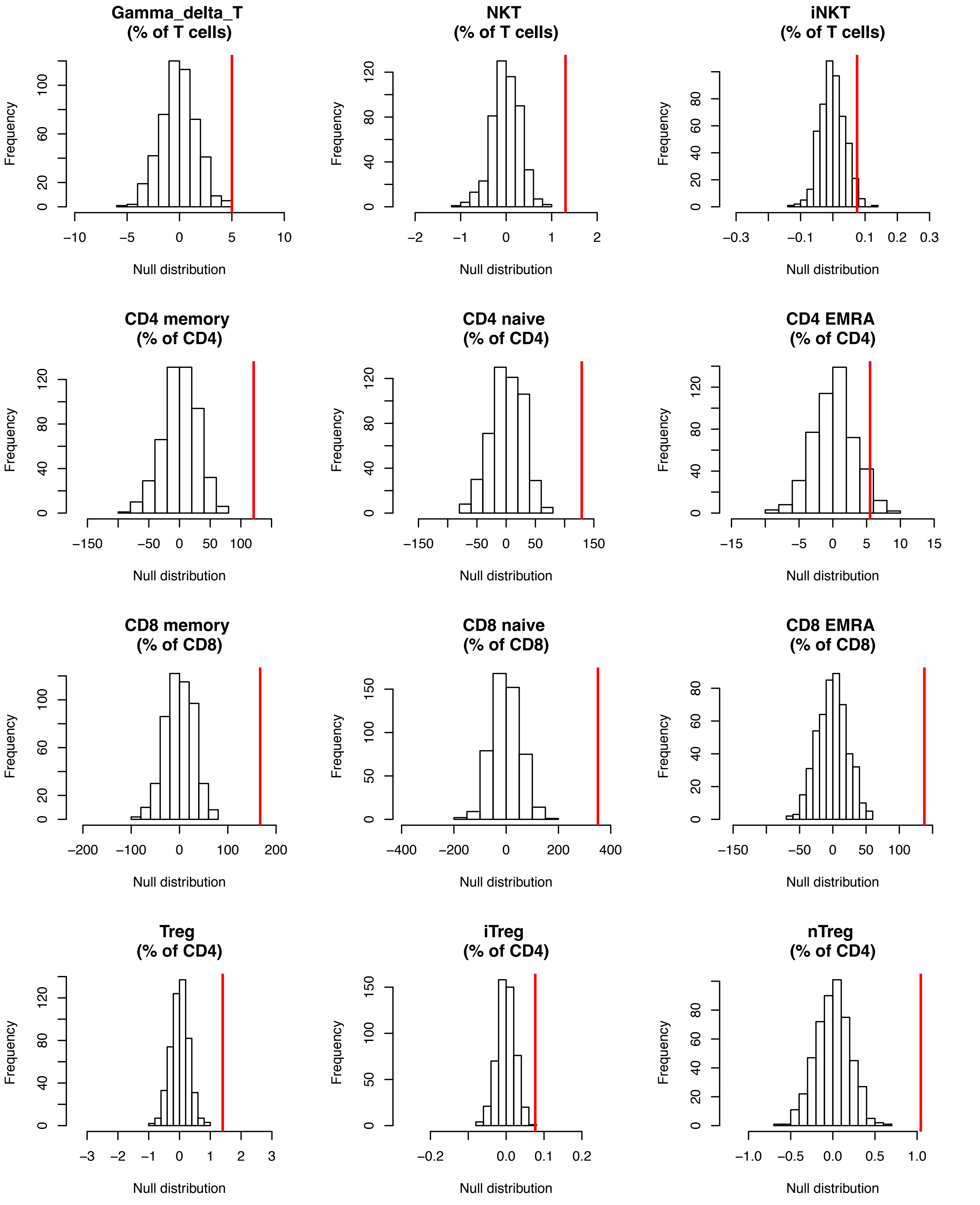

Gamma_delta_T
(% of T cells)
NKT
(% of T cells)
iNKT
(% of T cells)
CD4 memory
(% of CD4)
CD4 naive
(% of CD4)
CD4 EMRA
(% of CD4)
CD8 memory
(% of CD8)
CD8 naive
(% of CD8)
CD8 EMRA
(% of CD8)
Treg
(% of CD4)
iTreg
(% of CD4)
nTreg
(% of CD4)
Frequency
Null distribution

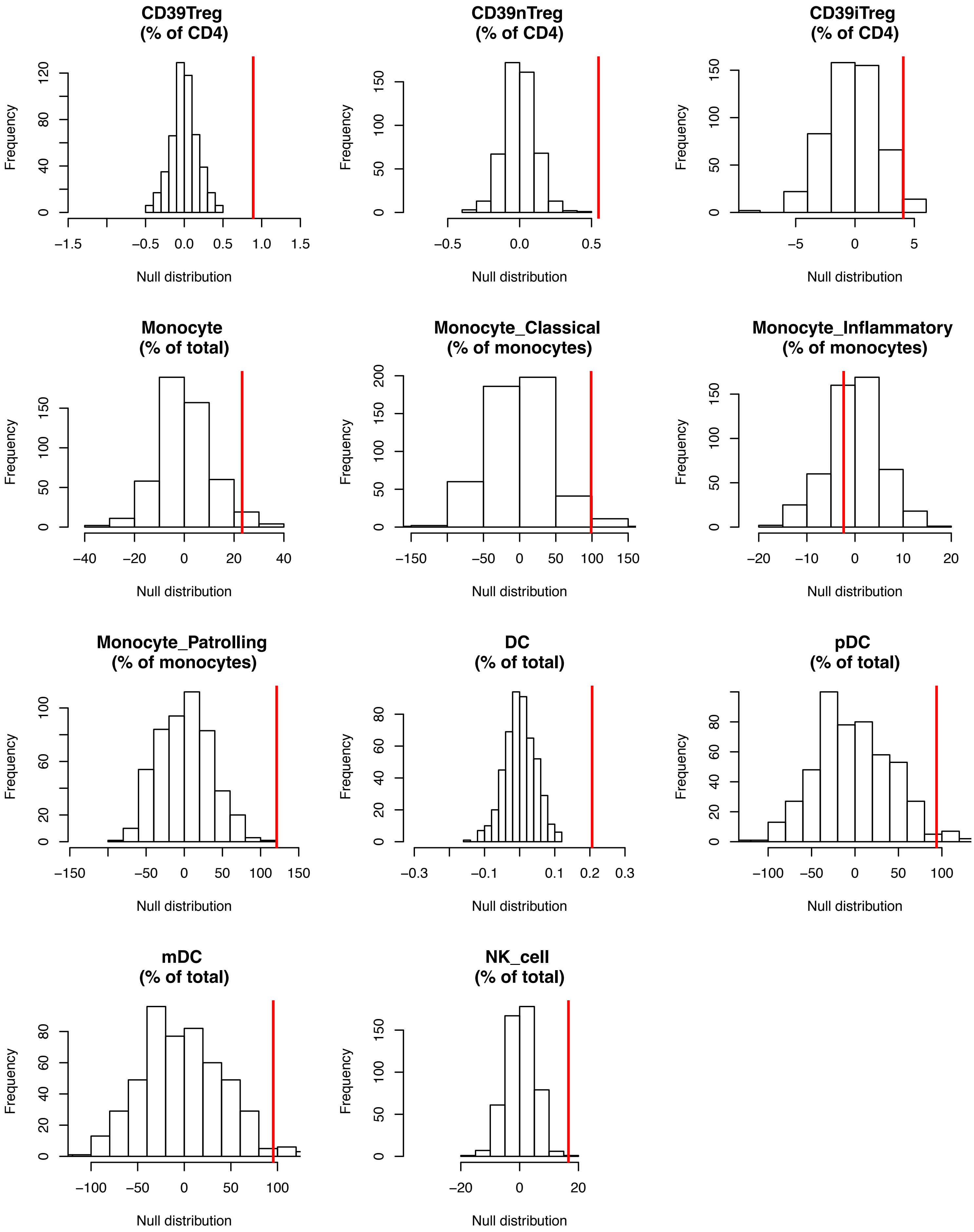

CD39Treg
(% of CD4)
Frequency
Null distribution
CD39nTreg
(% of CD4)
Frequency
Null distribution
CD39iTreg
(% of CD4)
Frequency
Null distribution
Monocyte
(% of total)
Frequency
Null distribution
Monocyte_Classical
(% of monocytes)
Frequency
Null distribution
Monocyte_Inflammatory
(% of monocytes)
Frequency
Null distribution
Monocyte_Patrolling
(% of monocytes)
Frequency
Null distribution
DC
(% of total)
Frequency
Null distribution
pDC
(% of total)
Frequency
Null distribution
mDC
(% of total)
Frequency
Null distribution
NK_cell
(% of total)
Frequency
Null distribution

SUPPLEMENTARY FIGURES

Characterization of biological variation of peripheral blood immune cytome in an Indian cohort

Parna Kanodia, Gurvinder Kaur, Poonam Coshic, Kabita Chatterjee, Teresa Neeman, Anna George, Satyajit Rath, Vineeta Bal, Savit B Prabhu.

Supplementary Figure 6

Principal component analysis (PCA) plots of data shown in Figure 5D with gender and age distribution of individuals. Each dot represents an individual. PCA is based on the temporal fluctuation of all immune subsets quantified.

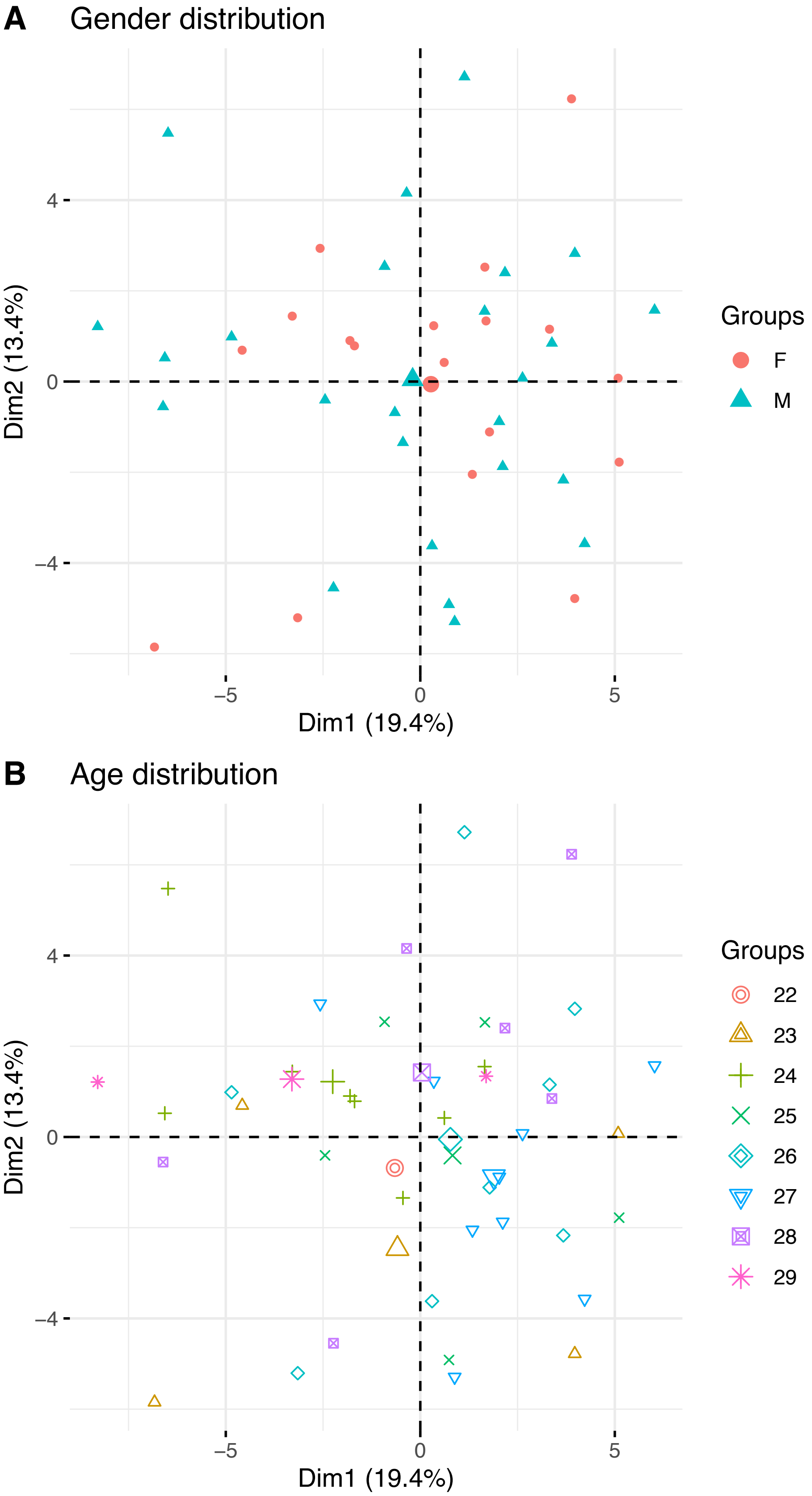
A
Gender distribution
Dim2 (13.4%)
Dim1 (19.4%)
4
0
−4
−5
0
5
Groups
F
M
B
Age distribution
Dim2 (13.4%)
Dim1 (19.4%)
4
0
−4
−5
0
5
Groups
22
23
24
25
26
27
28
29

SUPPLEMENTARY FIGURES

Characterization of biological variation of peripheral blood immune cytome in an Indian cohort

Parna Kanodia, Gurvinder Kaur, Poonam Coshic, Kabita Chatterjee, Teresa Neeman, Anna George, Satyajit Rath, Vineeta Bal, Savit B Prabhu.

Supplementary Figure 7

Immune subsets that show positive correlation with each other. Correlation coefficients and p-values are shown in Supplementary Table S8. Dots indicate frequency of the cell subset (expressed as % of parent gate) as indicated. This supplementary figure also refers to Figure 6 and Figure 7.

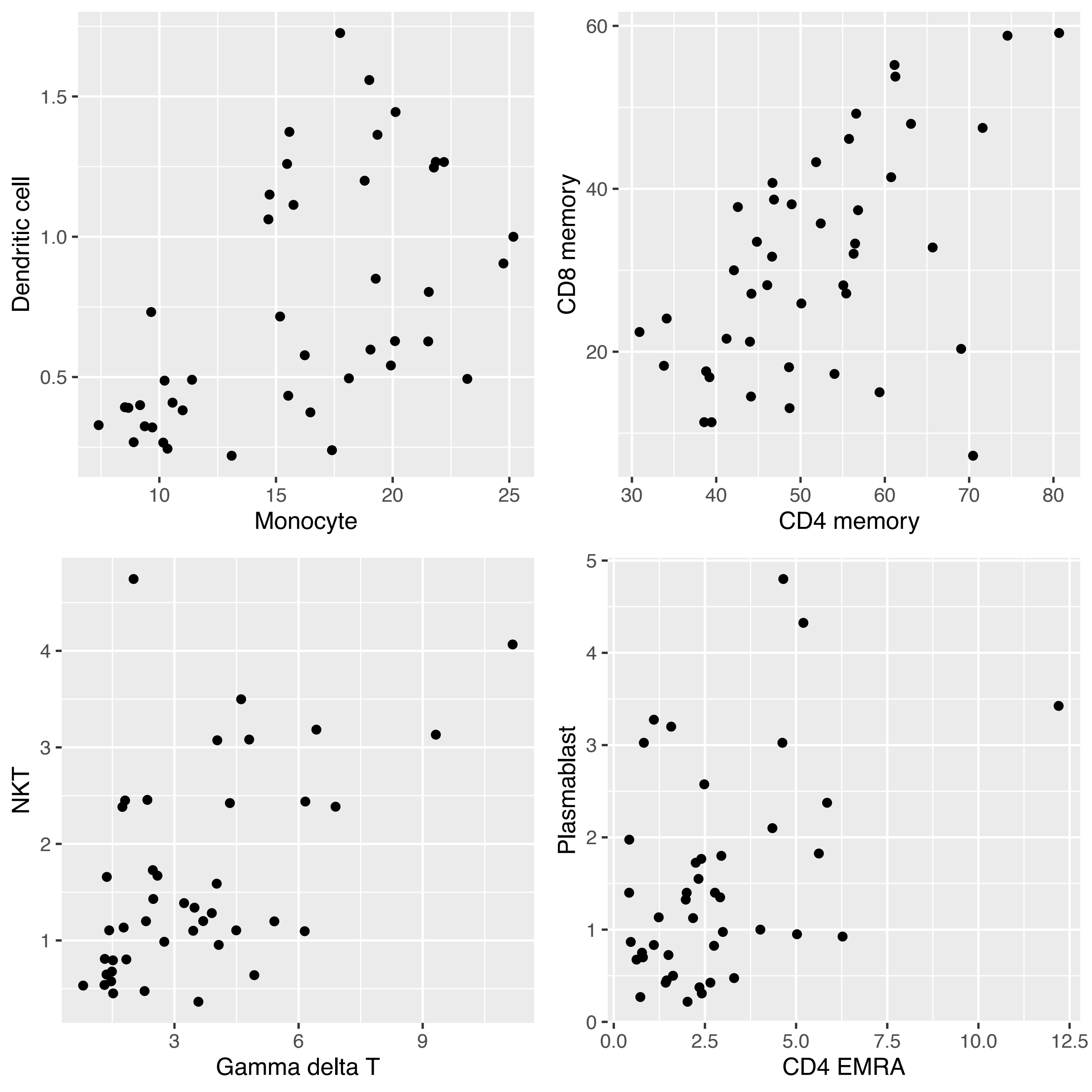

Dendritic cell
1.5
1.0
0.5
10
15
20
25
Monocyte
CD8 memory
60
40
20
30
40
50
60
70
80
CD4 memory
NKT
4
3
2
1
3
6
9
Gamma delta T
Plasmablast
5
4
3
2
1
0
0.0
2.5
5.0
7.5
10.0
12.5
CD4 EMRA

SUPPLEMENTARY FIGURES

Characterization of biological variation of peripheral blood immune cytome in an Indian cohort

Parna Kanodia, Gurvinder Kaur, Poonam Coshic, Kabita Chatterjee, Teresa Neeman, Anna George, Satyajit Rath, Vineeta Bal, Savit B Prabhu.

Supplementary Figure 8

Immune subsets that show negative correlation with each other. Correlation coefficients and p-values are shown in Supplementary Table S8. Dots indicate frequency of the cell subset (expressed as % of parent gate) as indicated. This supplementary figure also refers to Figure 6 and Figure 7.

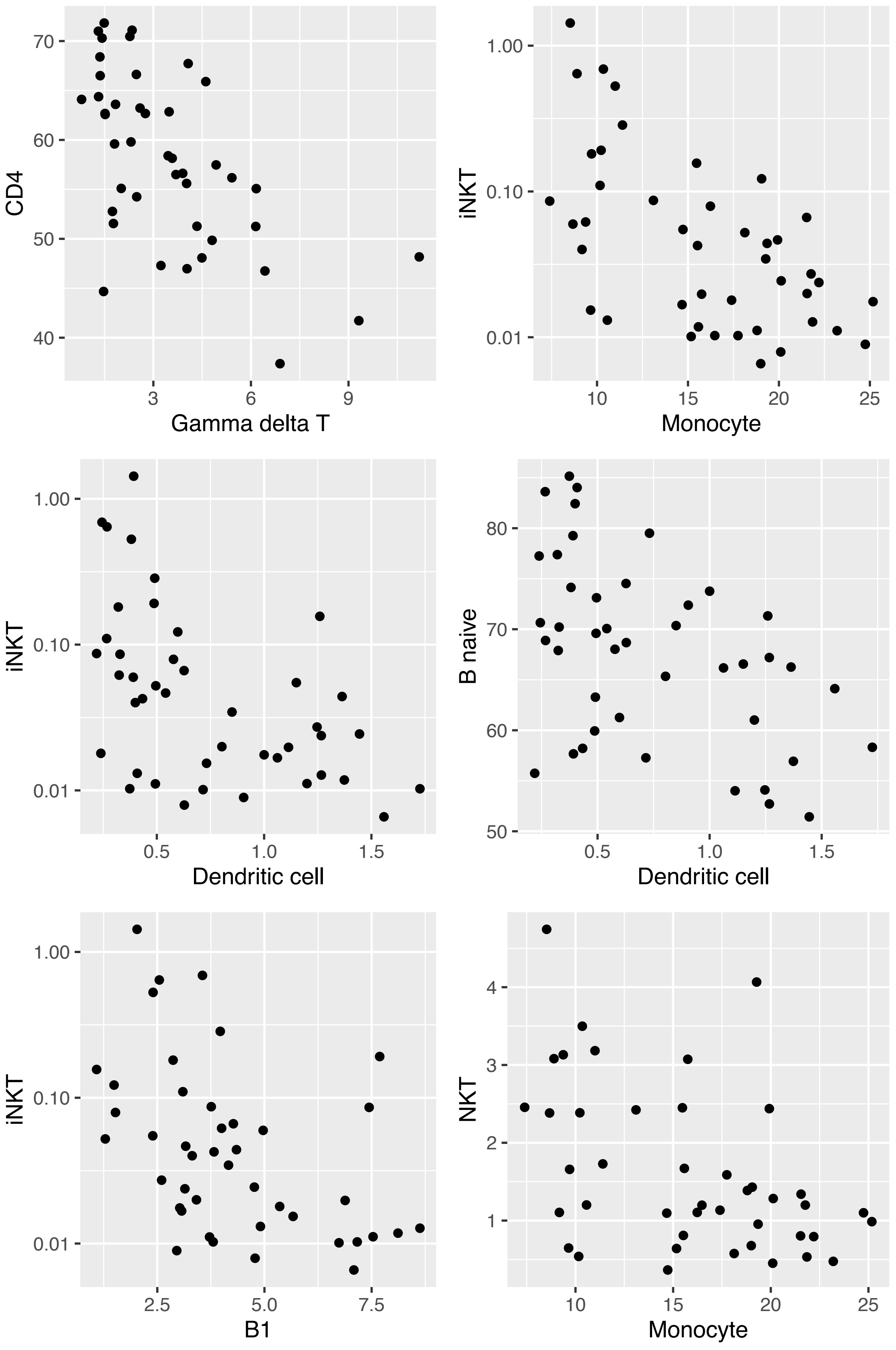
CD4
70
60
50
40
3
6
9
Gamma delta T
iNKT
1.00
0.10
0.01
10
15
20
25
Monocyte
iNKT
1.00
0.10
0.01
0.5
1.0
1.5
Dendritic cell
B naive
80
70
60
50
0.5
1.0
1.5
Dendritic cell
iNKT
1.00
0.10
0.01
2.5
5.0
7.5
B1
NKT
4
3
2
1
10
15
20
25
Monocyte

SUPPLEMENTARY FIGURES

Characterization of biological variation of peripheral blood immune cytome in an Indian cohort

Parna Kanodia, Gurvinder Kaur, Poonam Coshic, Kabita Chatterjee, Teresa Neeman, Anna George, Satyajit Rath, Vineeta Bal, Savit B Prabhu.

Supplementary Figure 9

Correlation heatmap showing correlation between degree of fluctuation of each immune parameter with others. Statistical analysis and p-values shown in Supplementary Table S9.

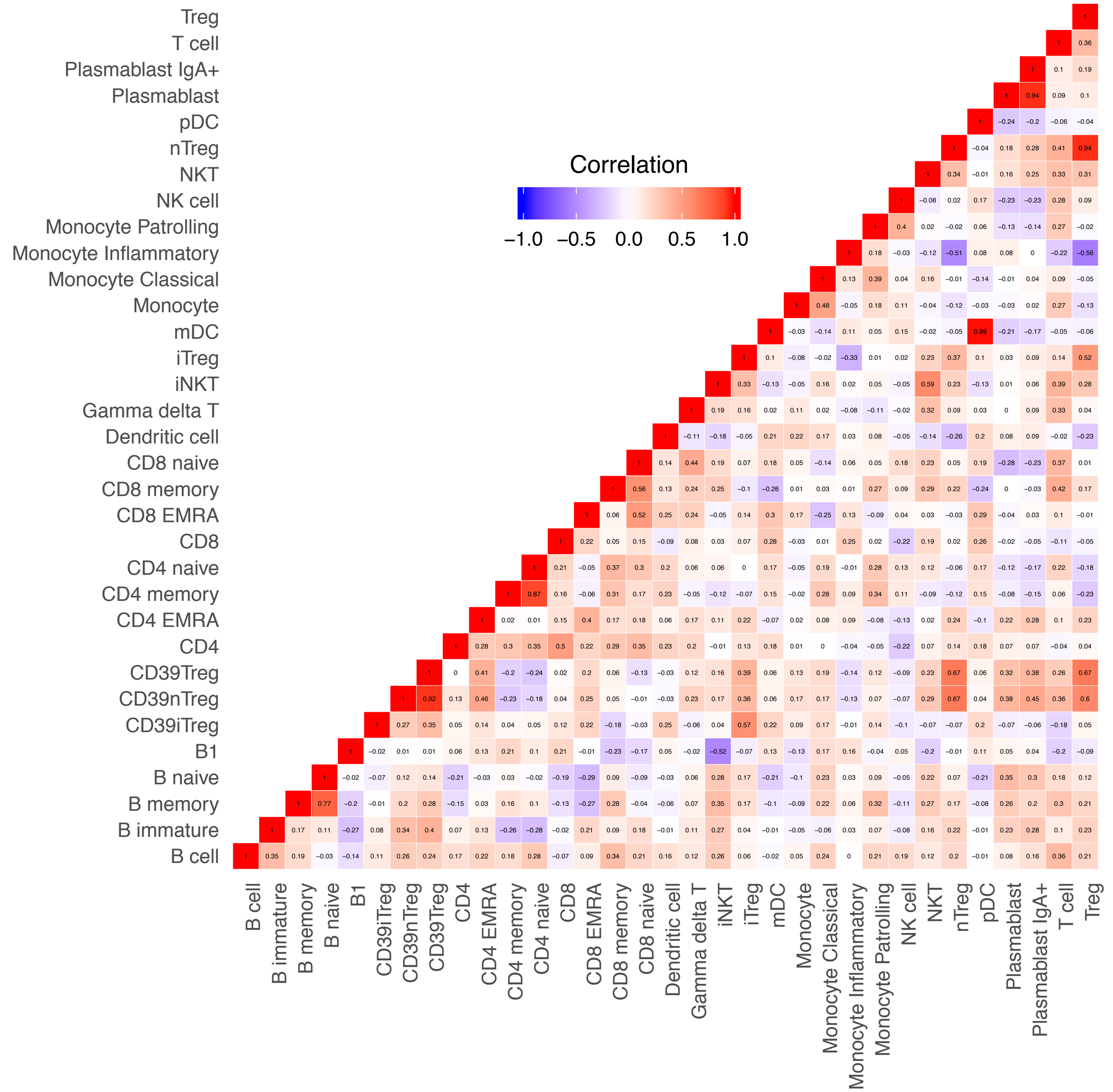
Correlation
−1.0
−0.5
0.0
0.5
1.0
Treg
T cell
Plasmablast IgA+
Plasmablast
pDC
nTreg
NKT
NK cell
Monocyte Patrolling
Monocyte Inflammatory
Monocyte Classical
Monocyte
mDC
iTreg
iNKT
Gamma delta T
Dendritic cell
CD8 naive
CD8 memory
CD8 EMRA
CD8
CD4 naive
CD4 memory
CD4 EMRA
CD4
CD39Treg
CD39nTreg
CD39iTreg
B1
B naive
B memory
B immature
B cell

SUPPLEMENTARY FIGURES

Characterization of biological variation of peripheral blood immune cytome in an Indian cohort

Parna Kanodia, Gurvinder Kaur, Poonam Coshic, Kabita Chatterjee, Teresa Neeman, Anna George, Satyajit Rath, Vineeta Bal, Savit B Prabhu.

Supplementary Figure 10

Comparison of immune parameter differences in sibling pairs and unrelated pairs. Histograms show the null distribution of differences between random pairs of individuals with permuted labels. Against this, the observed difference in unrelated pair minus sibling pair is shown as vertical line. The farther the vertical line from the center of the distribution, the greater is the difference in an unrelated pair compared to sibling pair. P-values are shown in Supplementary Table S11.

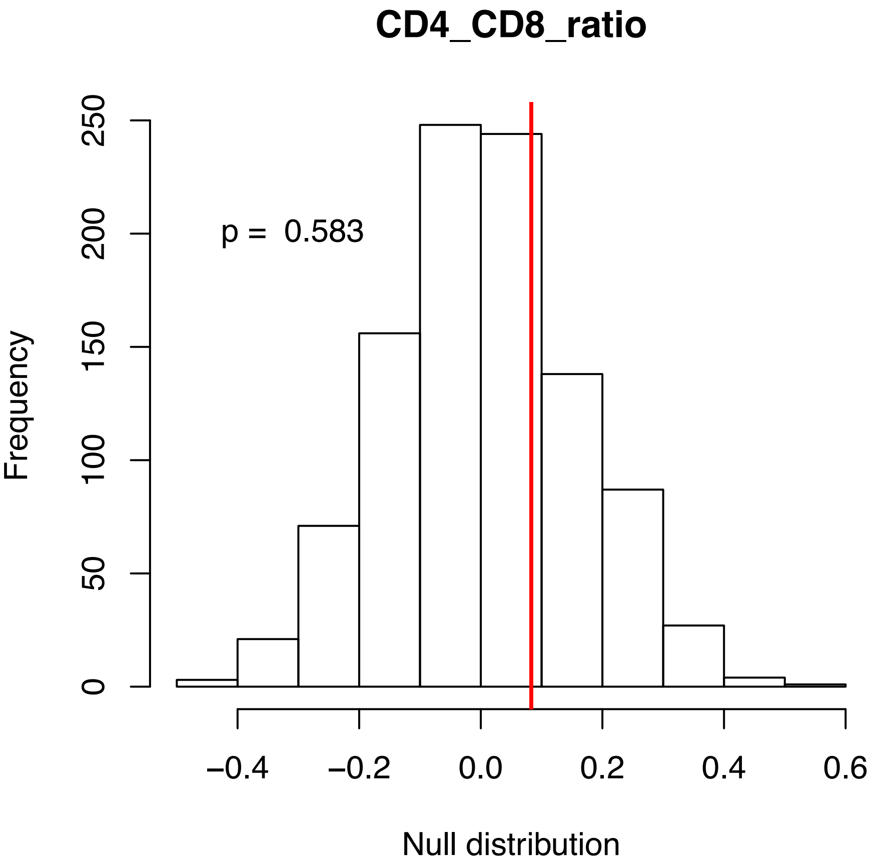
CD4_CD8_ratio
p = 0.583
Frequency
0
50
100
150
200
250
−0.4
−0.2
0.0
0.2
0.4
0.6
Null distribution

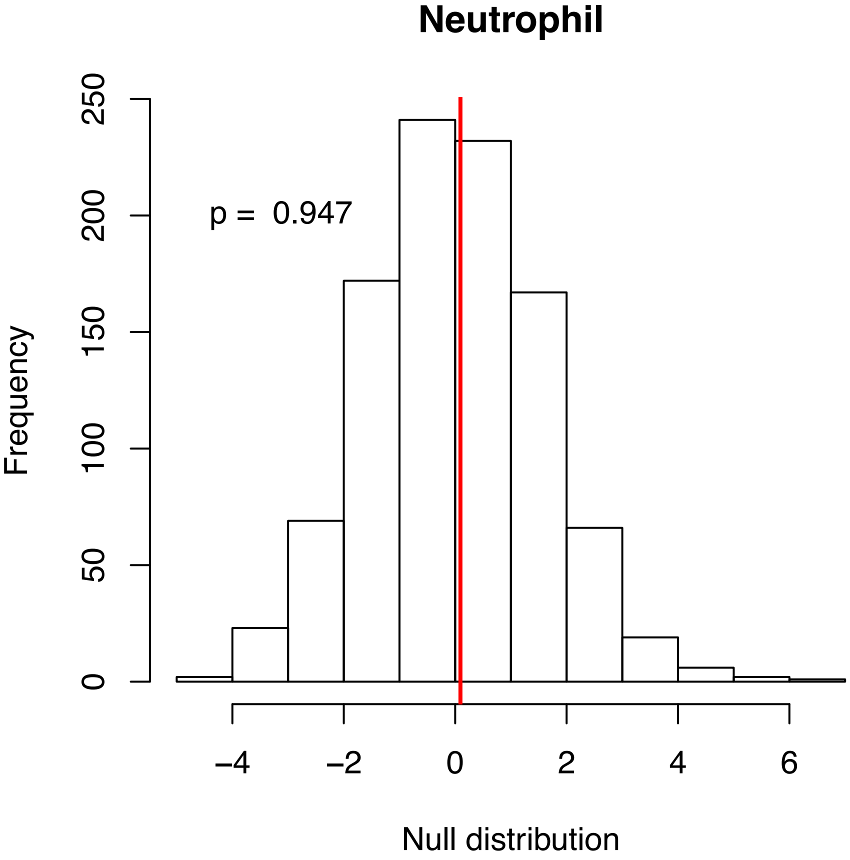
Neutrophil
p = 0.947
Frequency
0
50
100
150
200
250
−4
−2
0
2
4
6
Null distribution

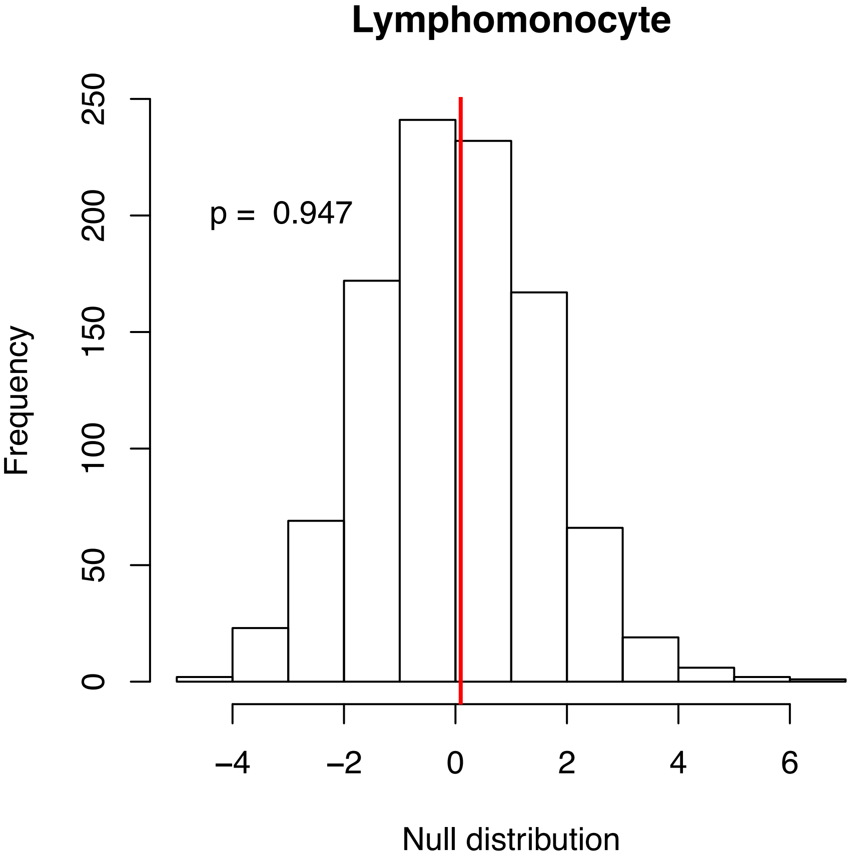

Lymphomonocyte
p = 0.947
Frequency
0
50
100
150
200
250
−4
−2
0
2
4
6
Null distribution

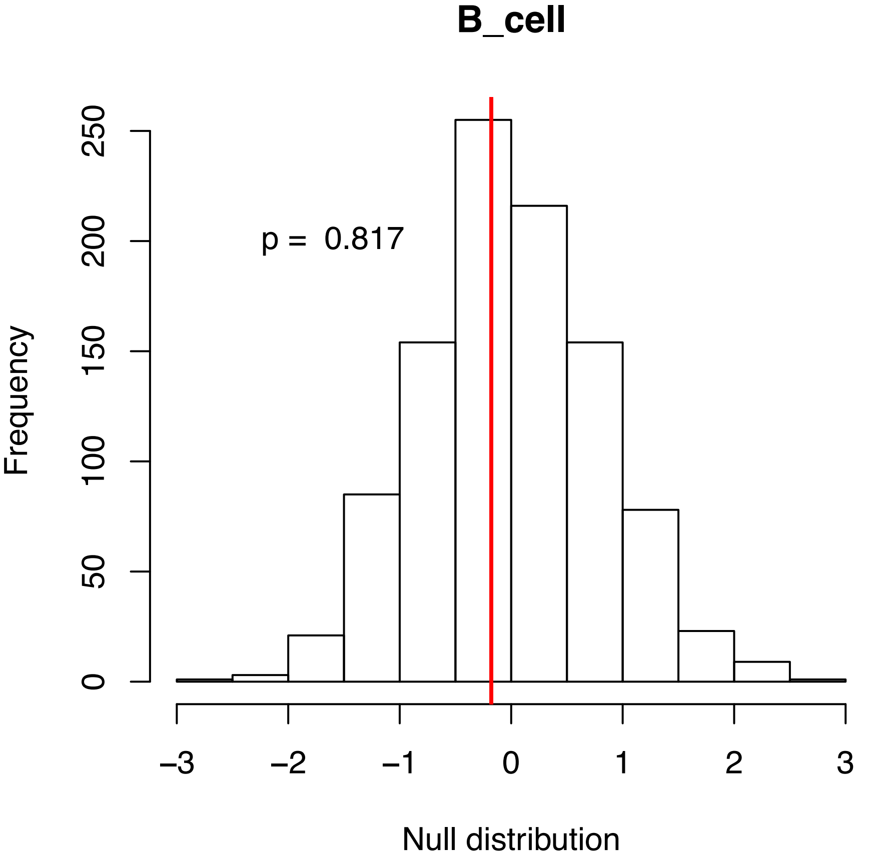

B_cell
p = 0.817
Frequency
0
50
100
150
200
250
−3
−2
−1
0
1
2
3
Null distribution

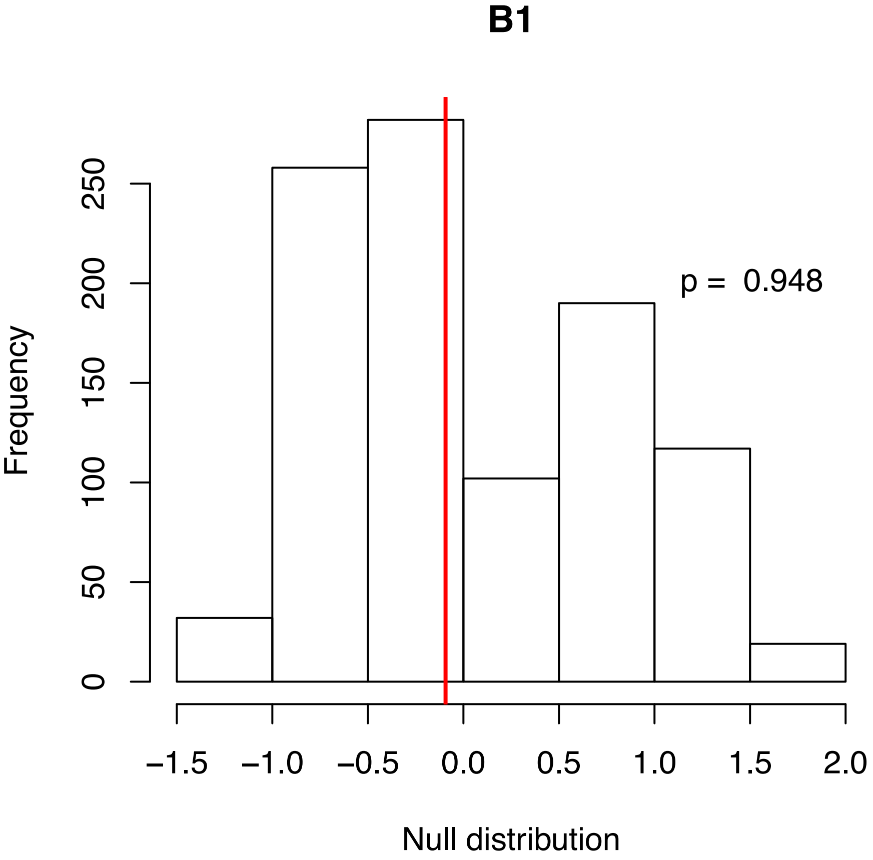
B1
p = 0.948
Frequency
0
50
100
150
200
250
−1.5
−1.0
−0.5
0.0
0.5
1.0
1.5
2.0
Null distribution

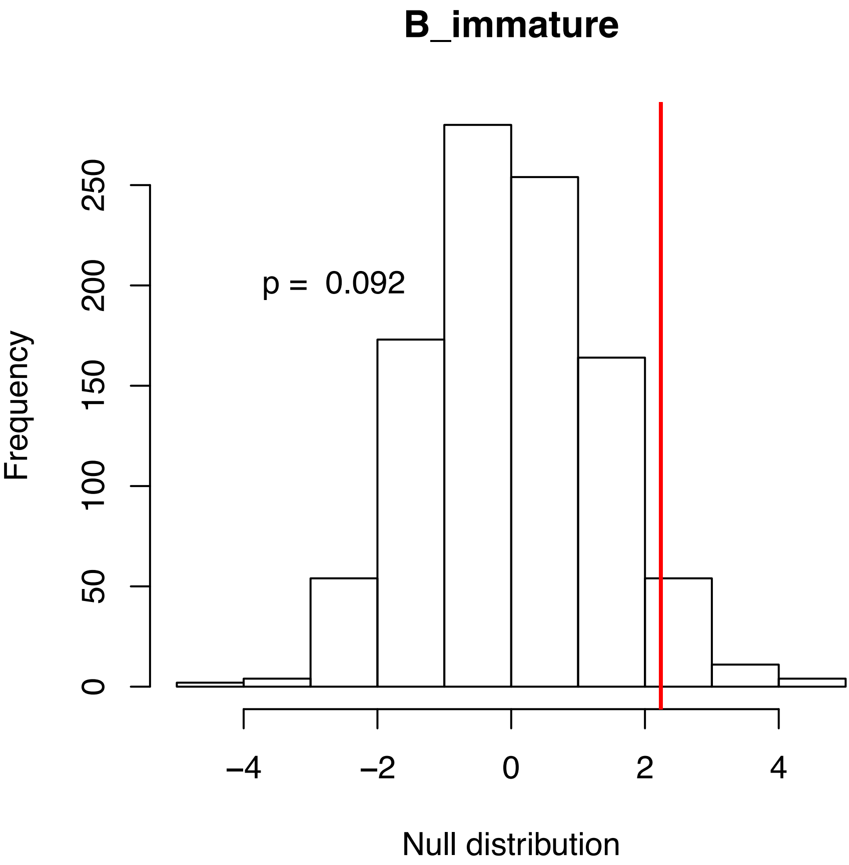

B_immature
p = 0.092
Frequency
0
50
100
150
200
250
−4
−2
0
2
4
Null distribution

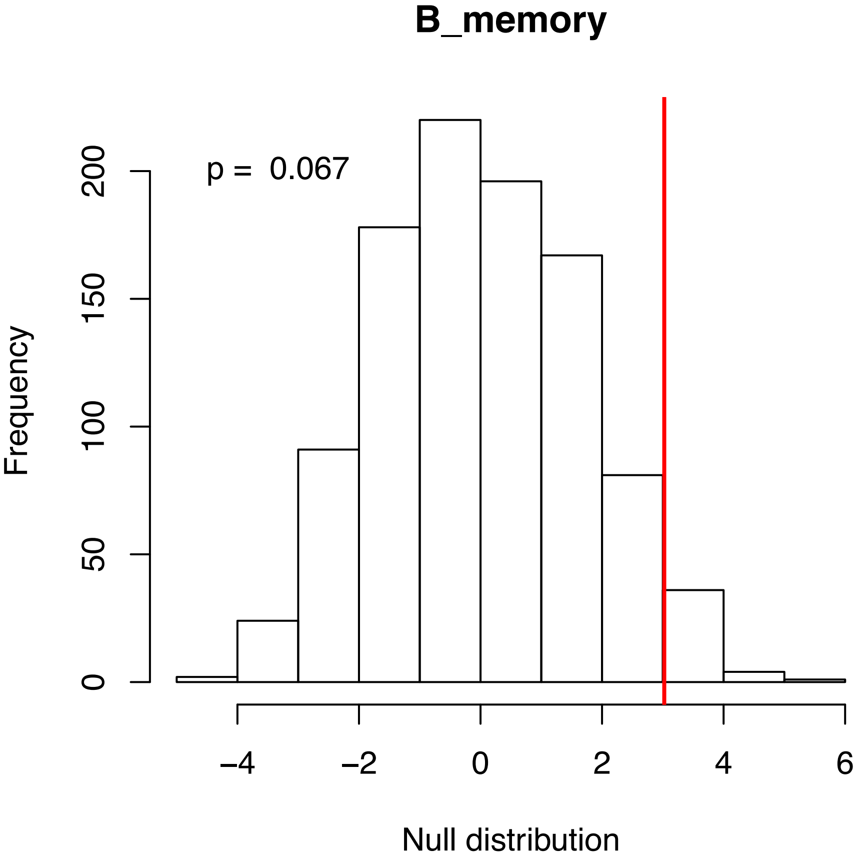

B_memory
p = 0.067
Frequency
0
50
100
150
200
−4
−2
0
2
4
6
Null distribution

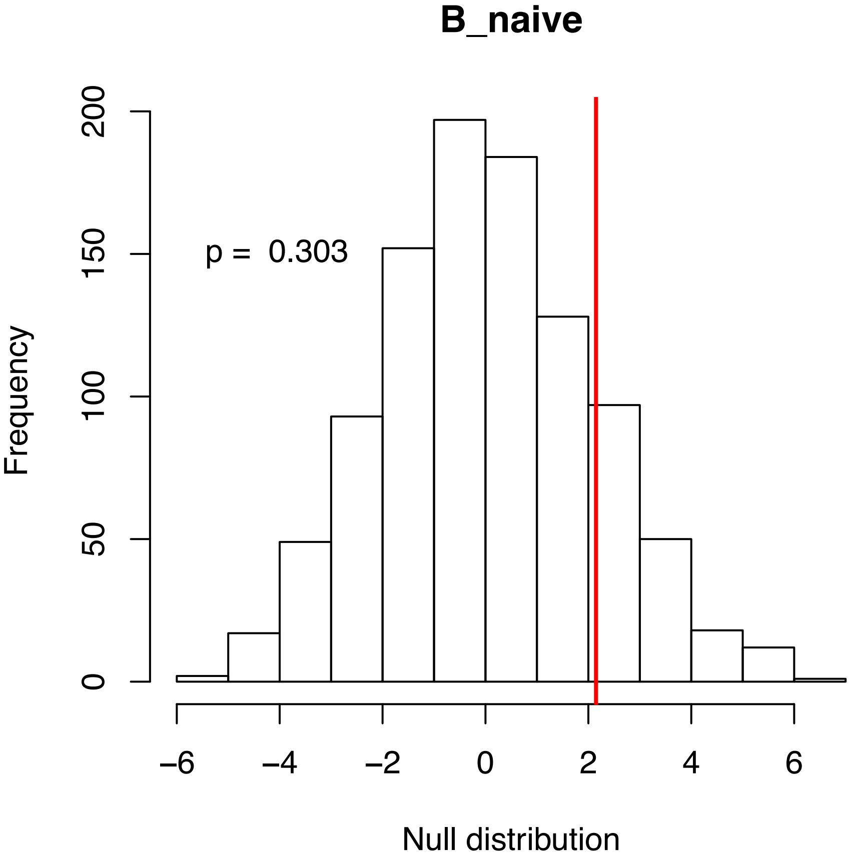

B_naive
p = 0.303
Frequency
0
50
100
150
200
−6
−4
−2
0
2
4
6
Null distribution

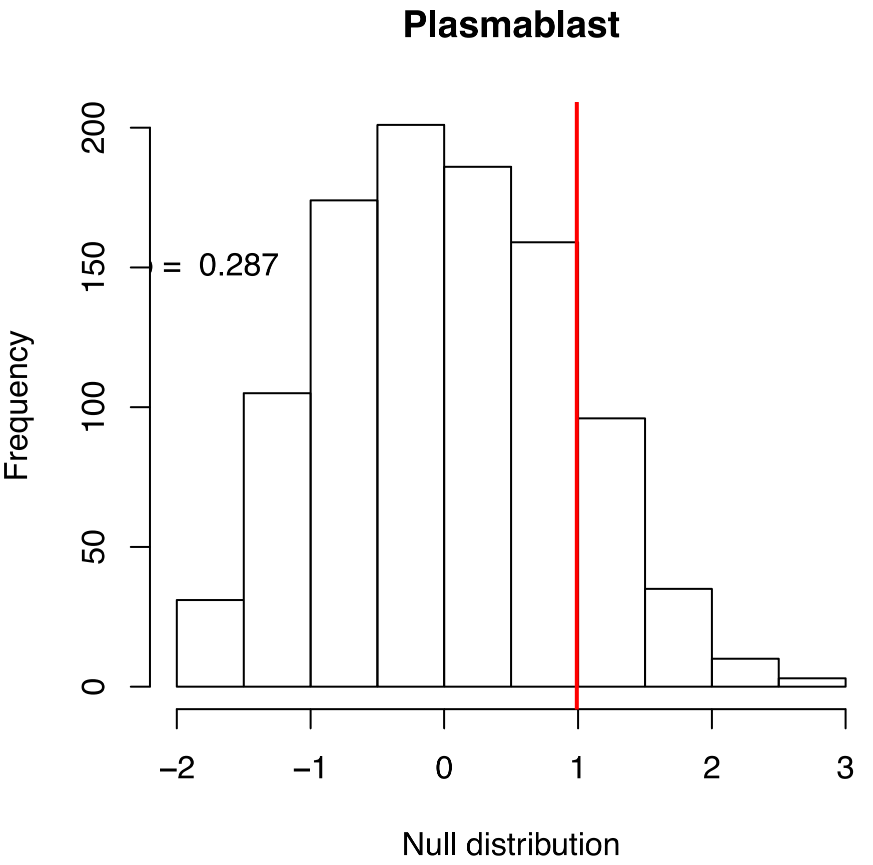

Plasmablast
= 0.287
Frequency
0
50
100
150
200
−2
−1
0
1
2
3
Null distribution

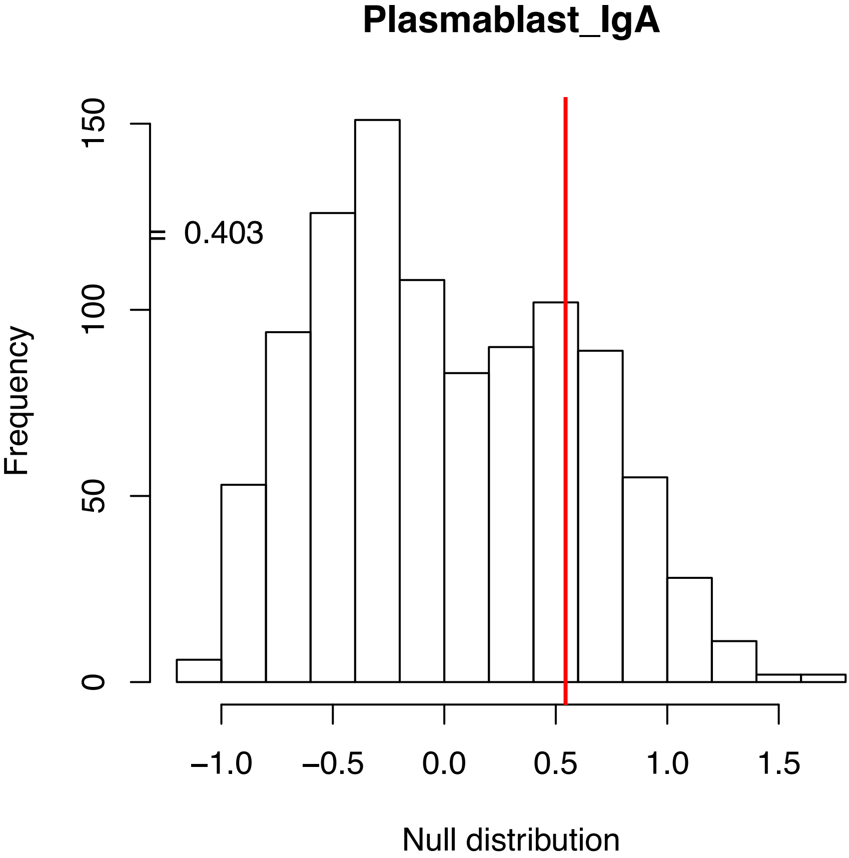

Plasmablast_IgA
= 0.403
150
100
50
0
Frequency
−1.0
−0.5
0.0
0.5
1.0
1.5
Null distribution

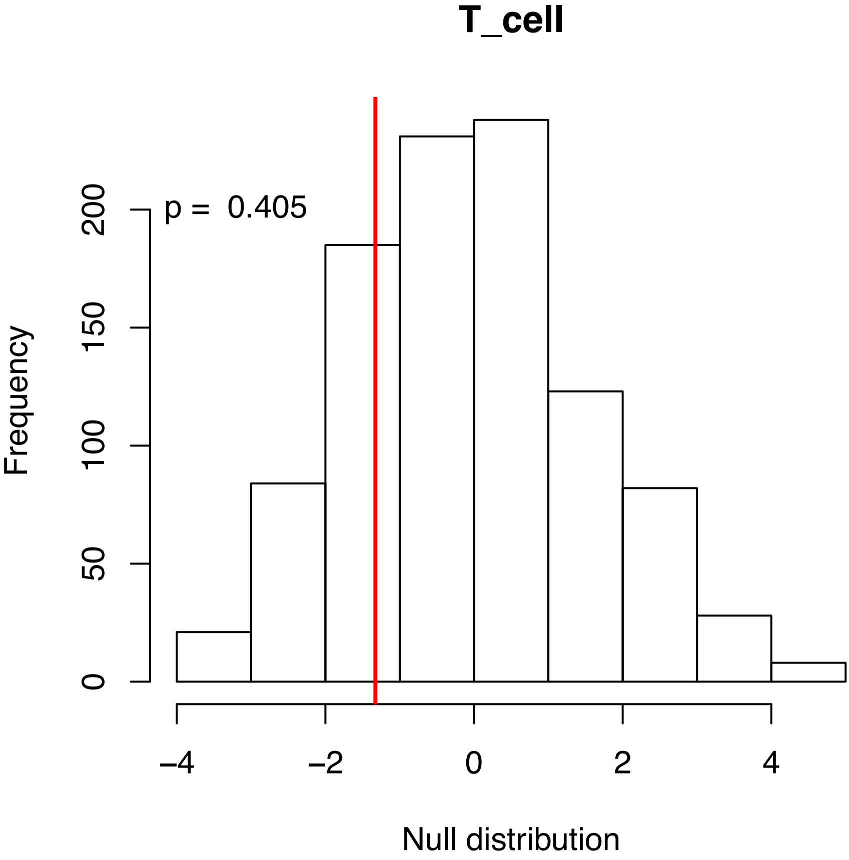

T_cell
p = 0.405
Frequency
0
50
100
150
200
−4
−2
0
2
4
Null distribution

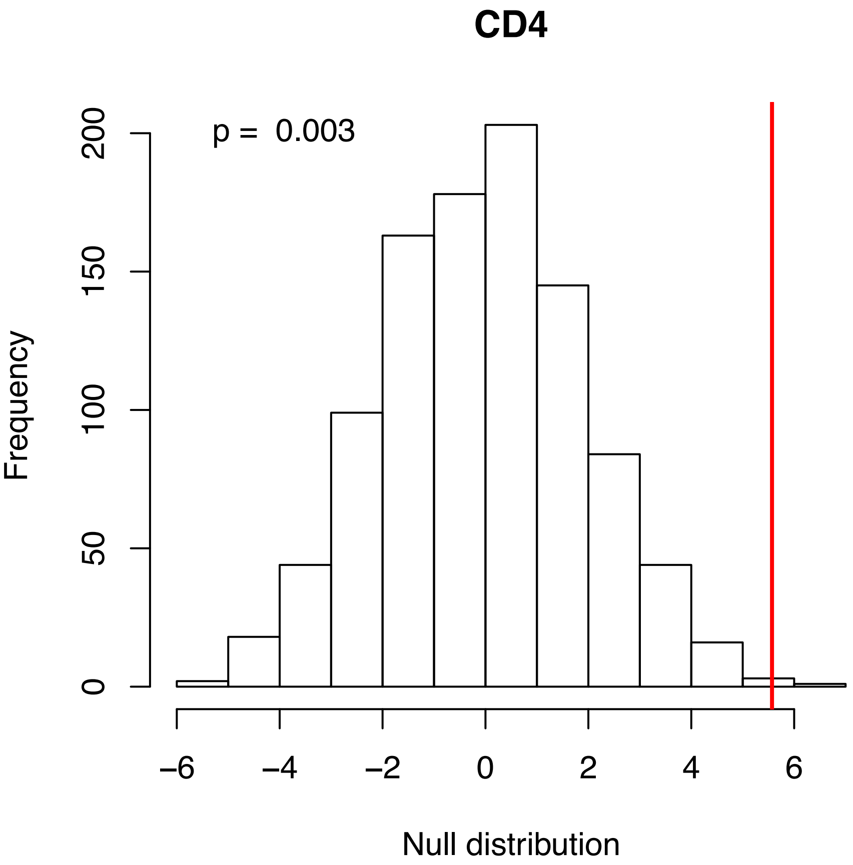

CD4
p = 0.003
Frequency
0
50
100
150
200
−6
−4
−2
0
2
4
6
Null distribution

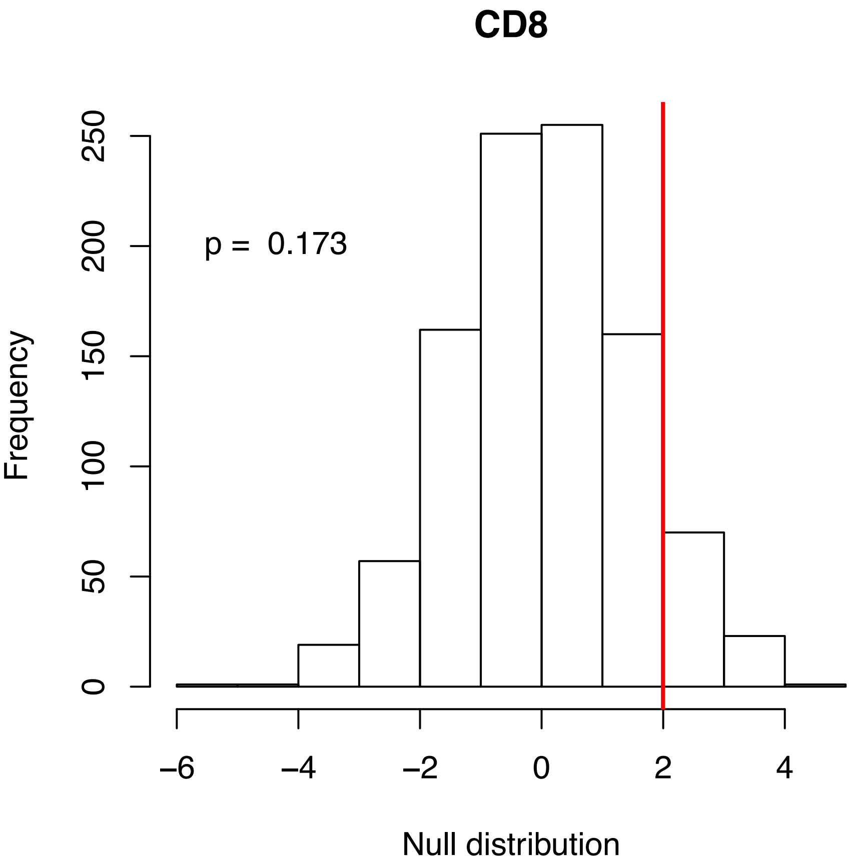

CD8
p = 0.173
Frequency
0
50
100
150
200
250
−6
−4
−2
0
2
4
Null distribution

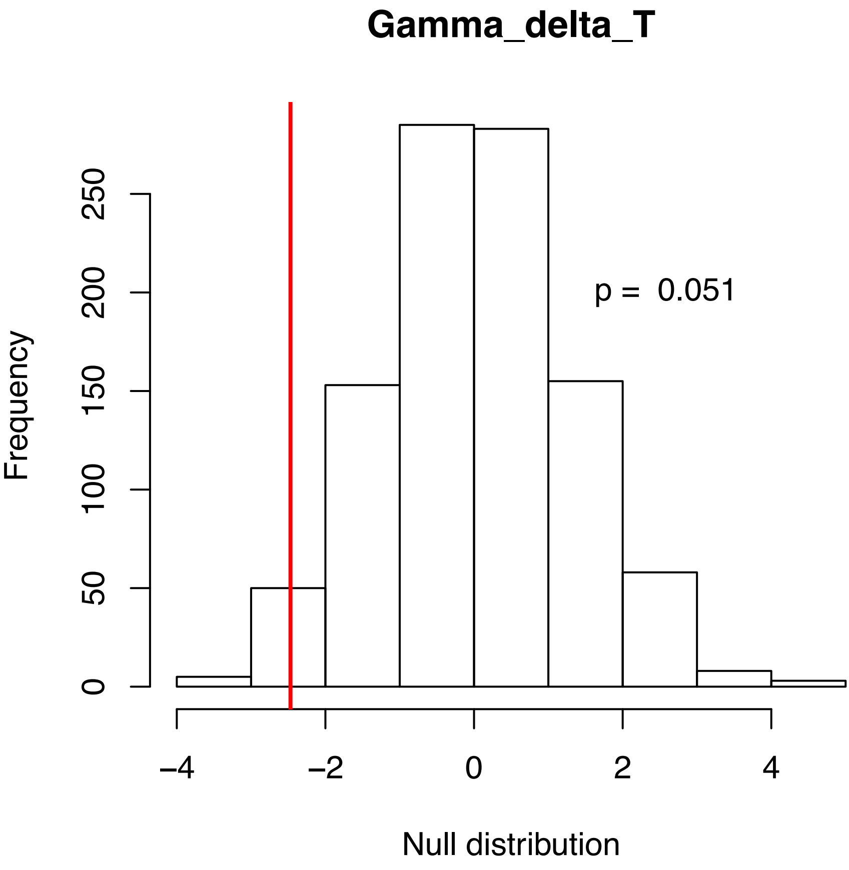

Gamma_delta_T
p = 0.051
Frequency
0
50
100
150
200
250
−4
−2
0
2
4
Null distribution

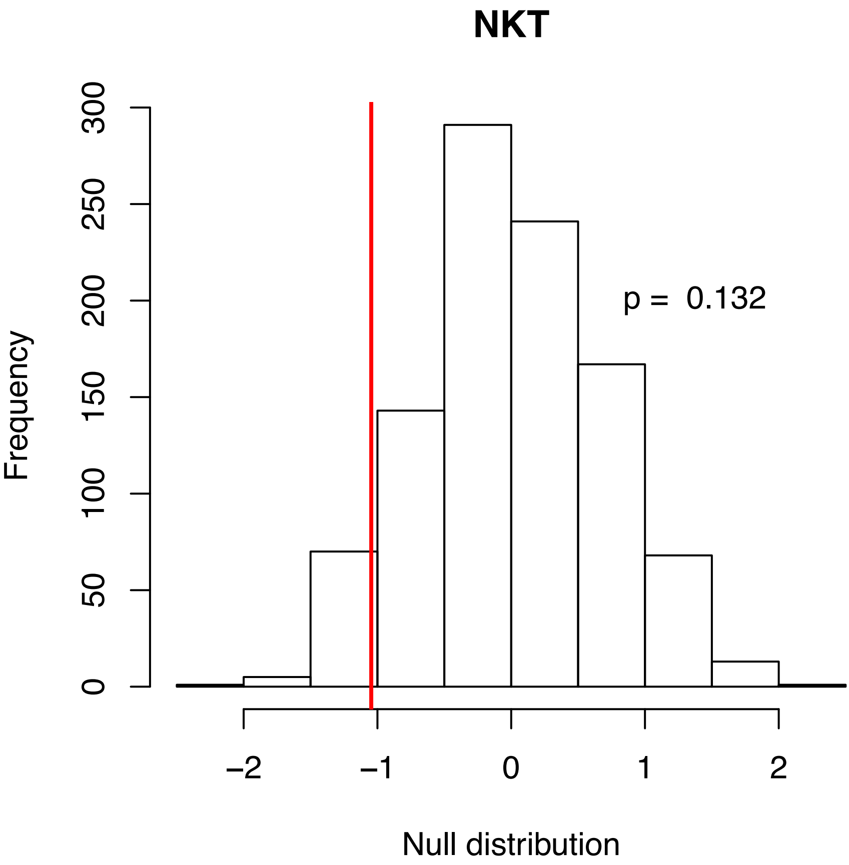
NKT
p = 0.132
Frequency
0
50
100
150
200
250
300
−2
−1
0
1
2
Null distribution

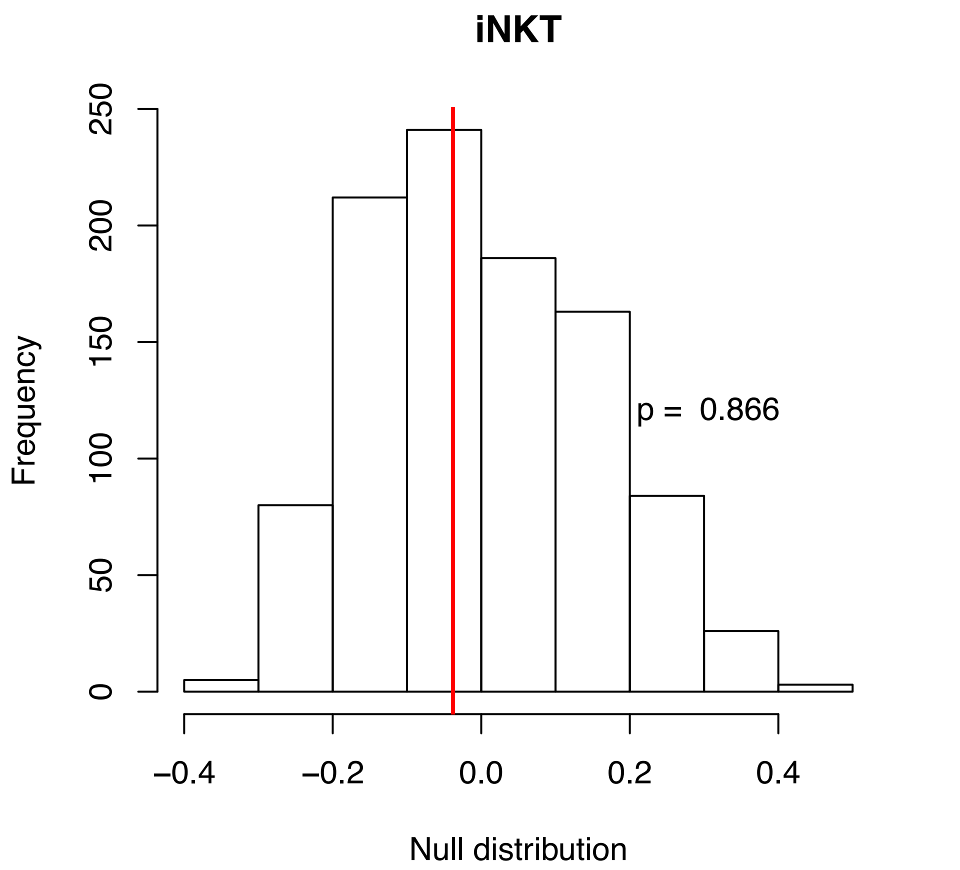

iNKT
Frequency
250
200
150
100
50
0
p = 0.866
−0.4
−0.2
0.0
0.2
0.4
Null distribution

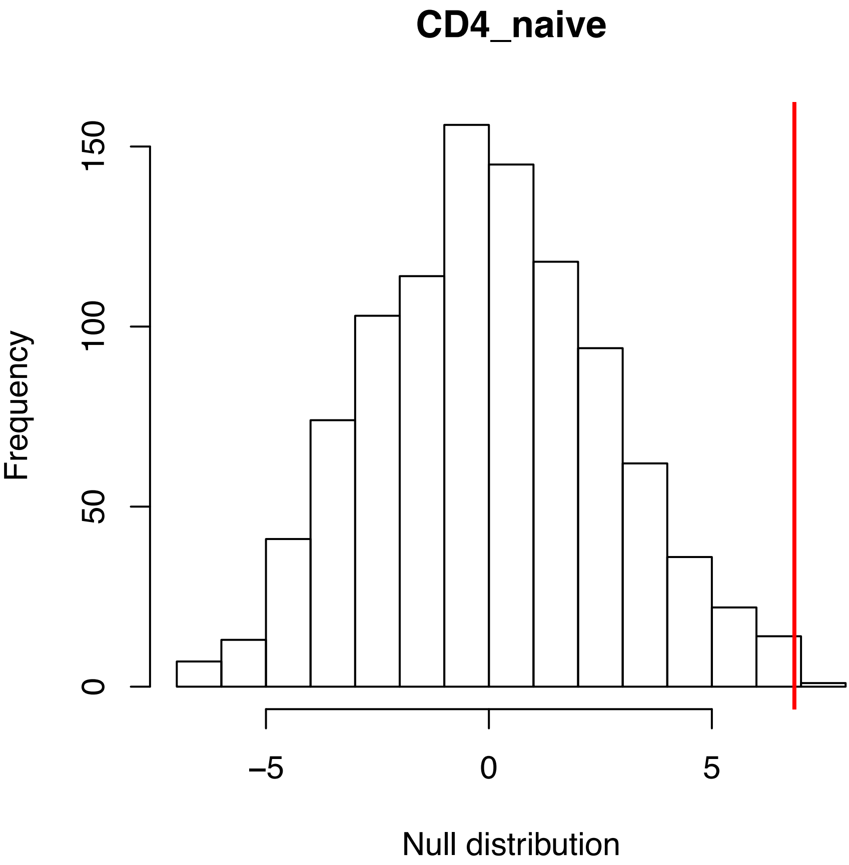

CD4_naive
Frequency
150
100
50
0
−5
0
5
Null distribution

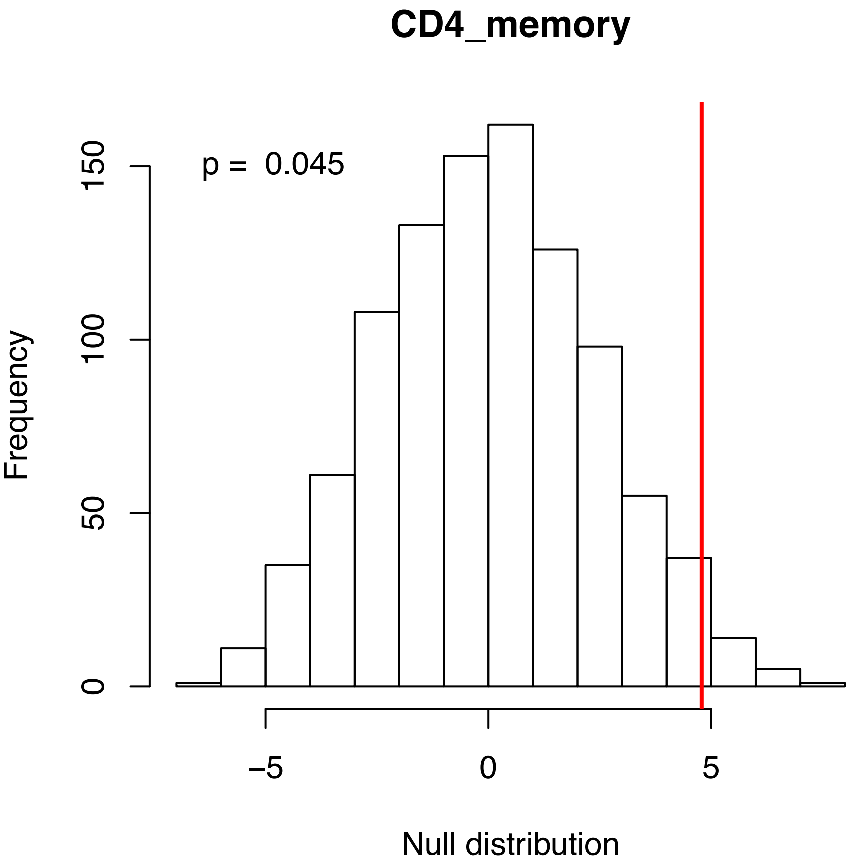
CD4_memory
p = 0.045
Frequency
0
50
100
150
−5
0
5
Null distribution

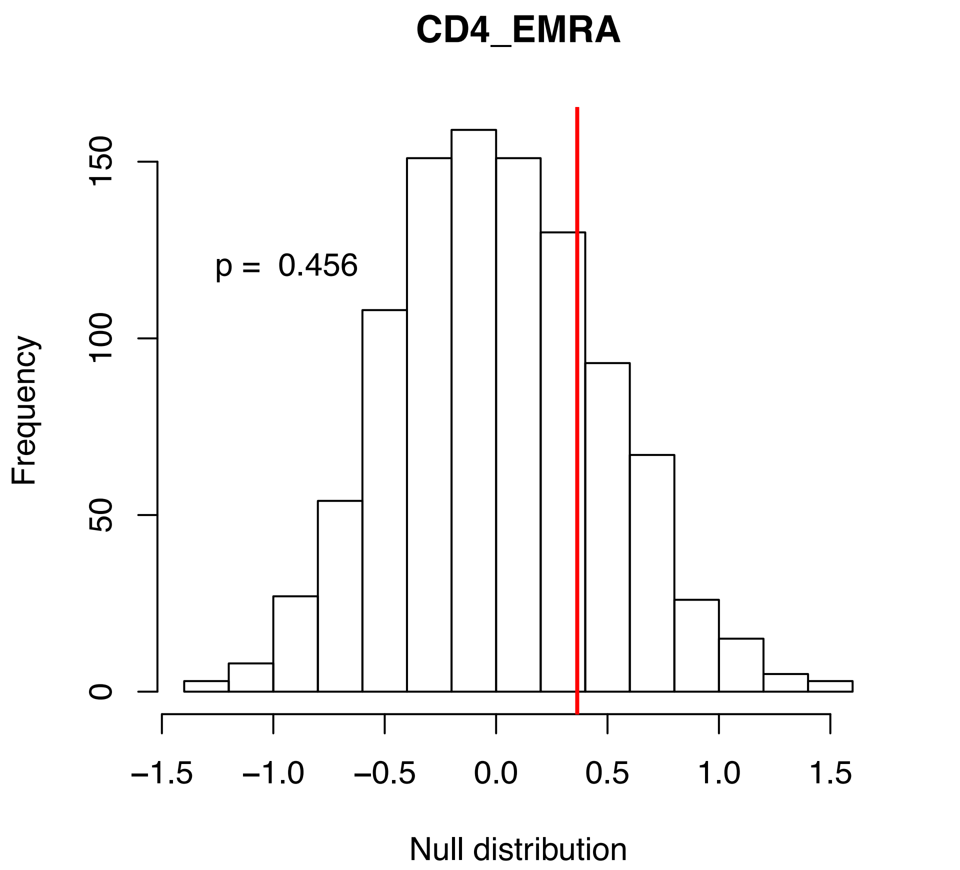

CD4_EMRA
p = 0.456
Frequency
0
50
100
150
−1.5
−1.0
−0.5
0.0
0.5
1.0
1.5
Null distribution

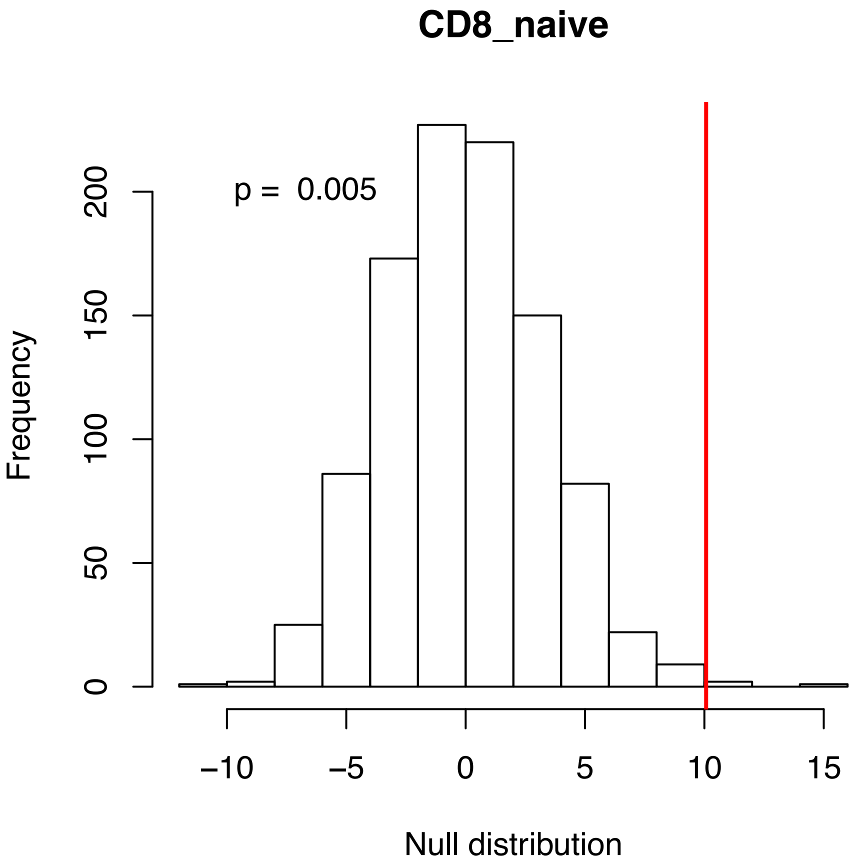

CD8_naive
p = 0.005
Frequency
0
50
100
150
200
−10
−5
0
5
10
15
Null distribution

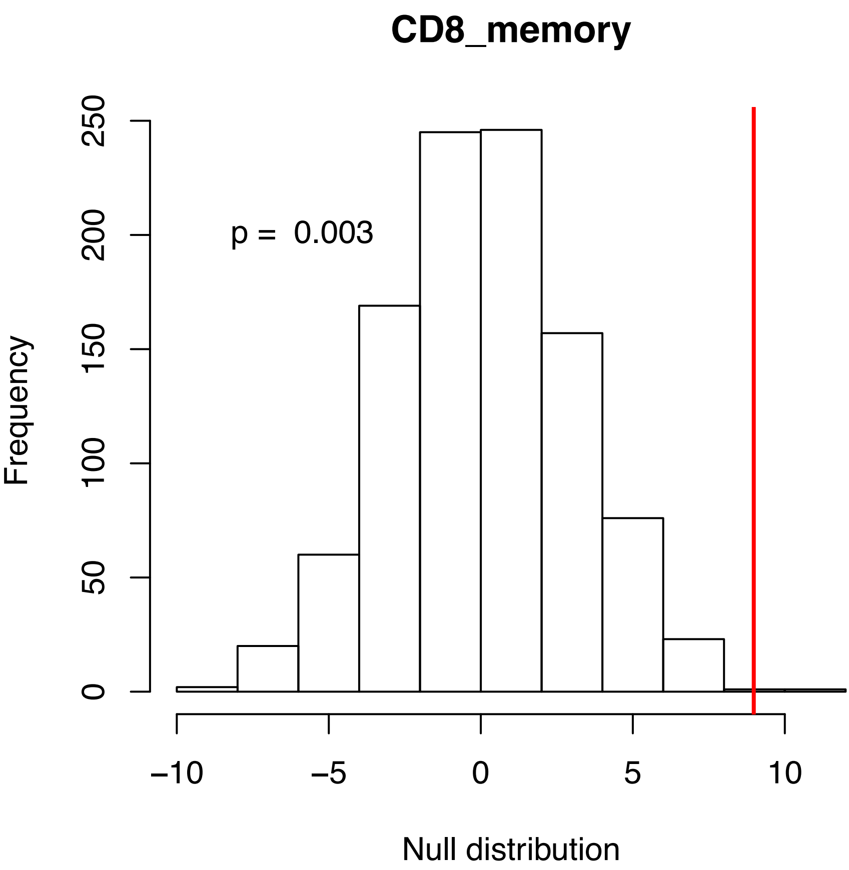

CD8_memory
p = 0.003
Frequency
250
200
150
100
50
0
−10
−5
0
5
10
Null distribution

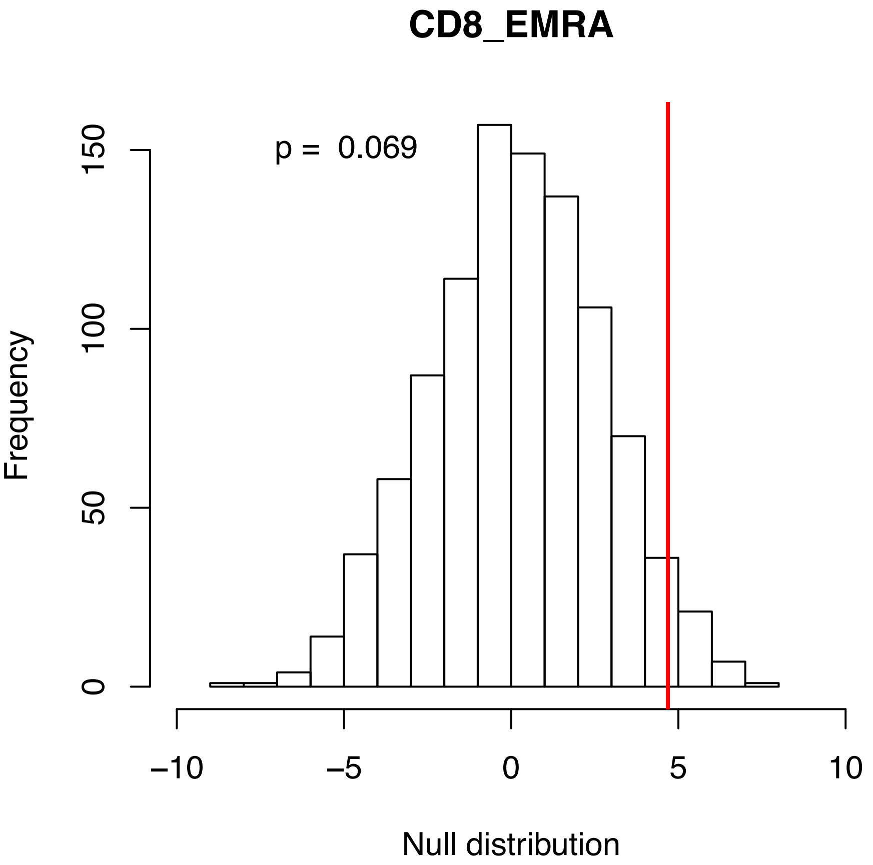

CD8_EMRA
p = 0.069
Frequency
0
50
100
150
−10
−5
0
5
10
Null distribution

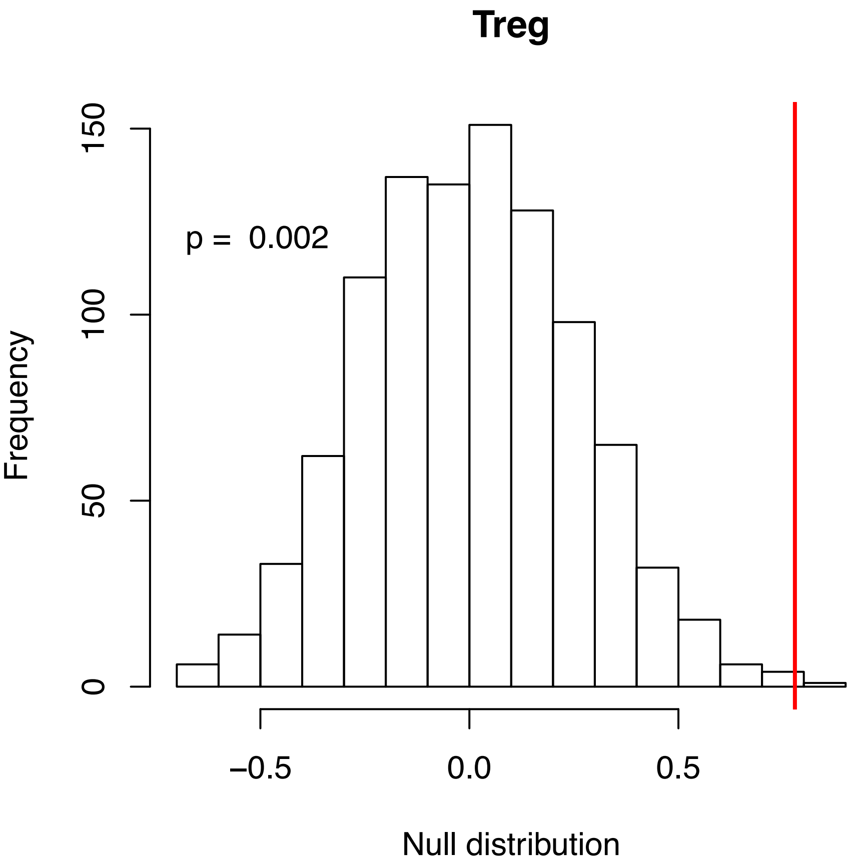
Treg
p = 0.002
Frequency
0
50
100
150
−0.5
0.0
0.5
Null distribution

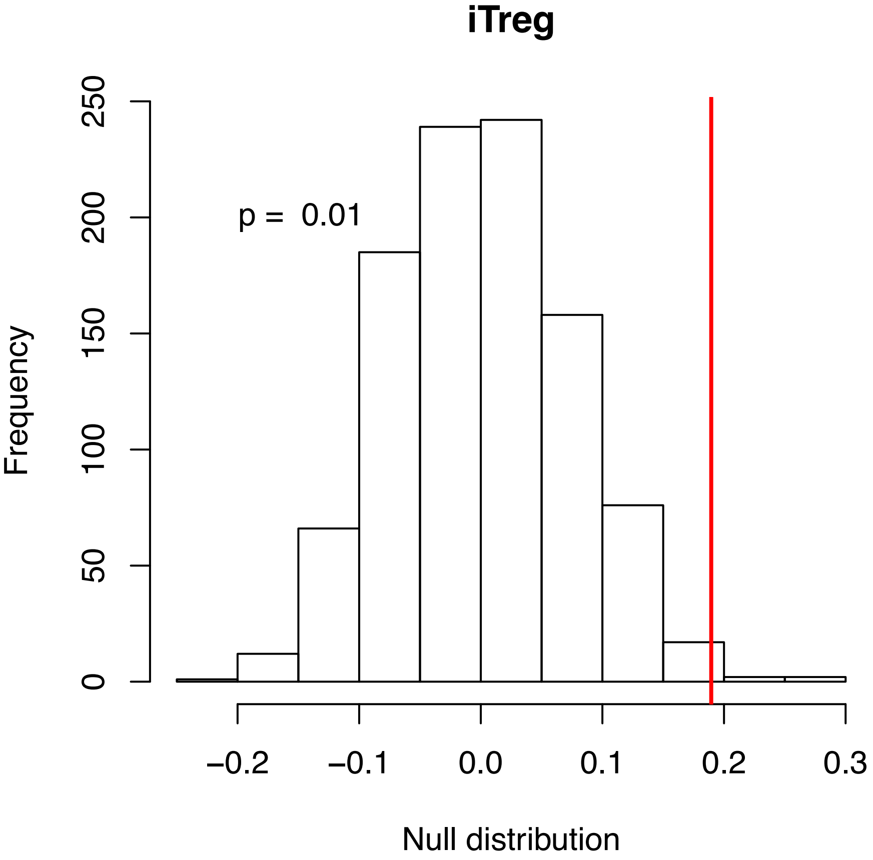
iTreg
p = 0.01
Frequency
0
50
100
150
200
250
−0.2
−0.1
0.0
0.1
0.2
0.3
Null distribution

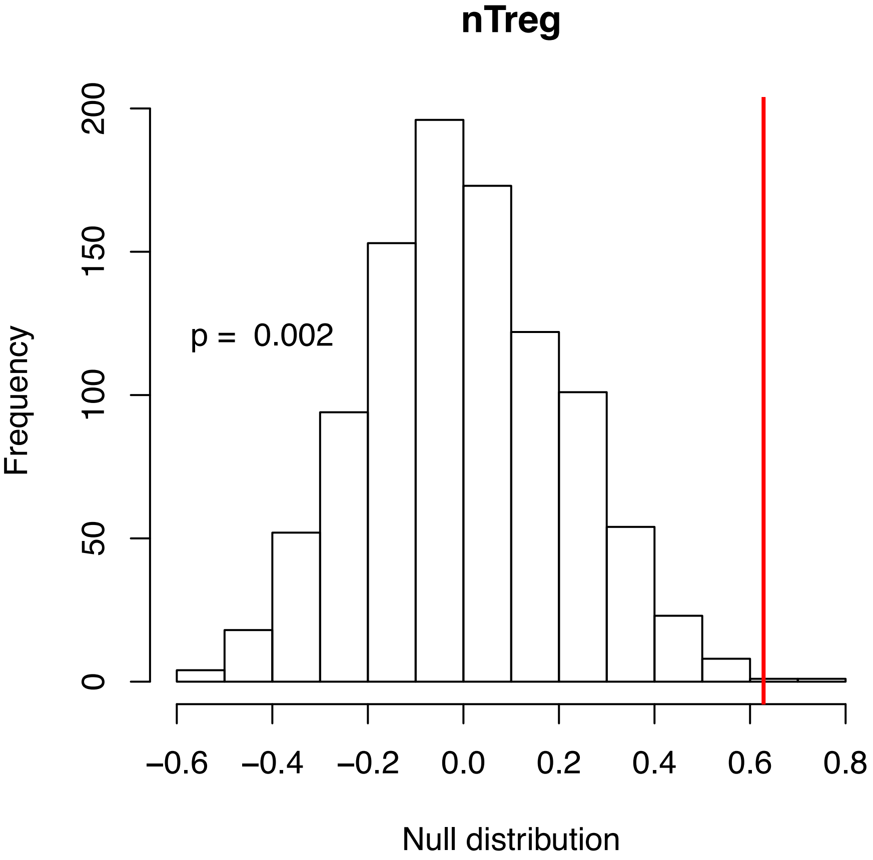
nTreg
p = 0.002
Frequency
0
50
100
150
200
−0.6
−0.4
−0.2
0.0
0.2
0.4
0.6
0.8
Null distribution

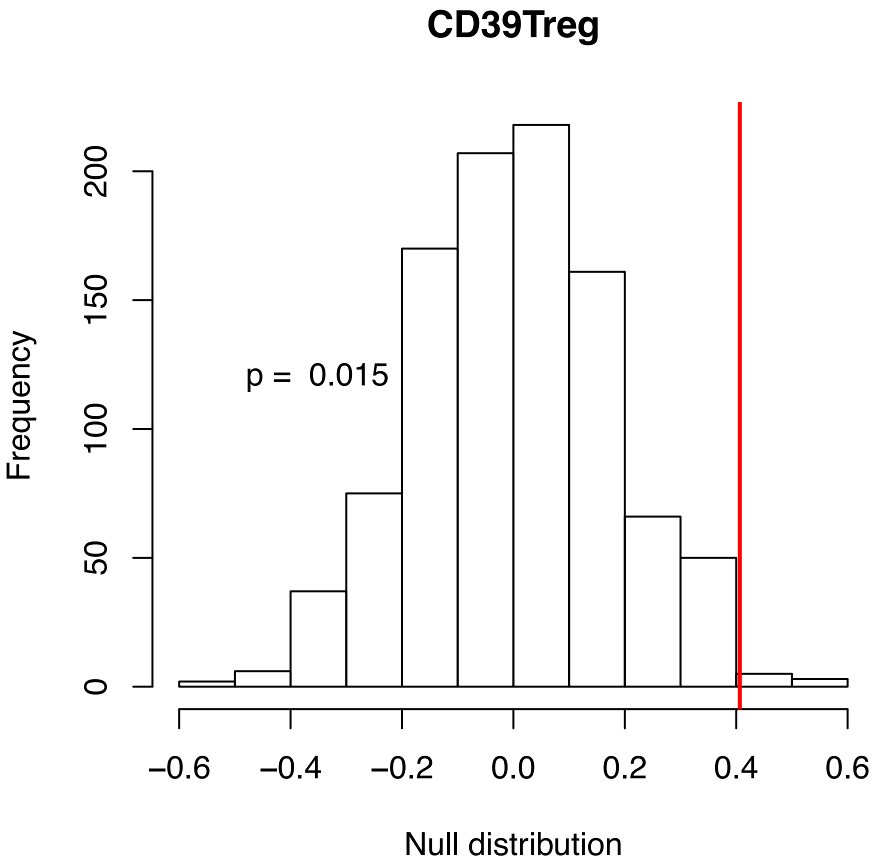
CD39Treg
Frequency
0
50
100
150
200
p = 0.015
−0.6
−0.4
−0.2
0.0
0.2
0.4
0.6
Null distribution

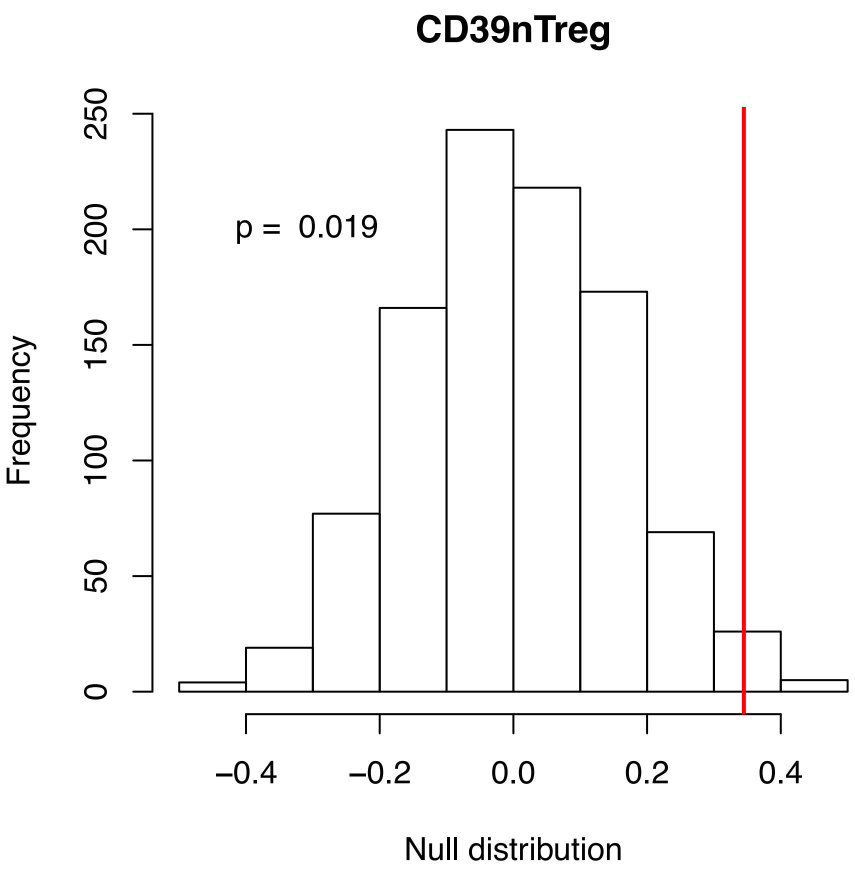
CD39nTreg
p = 0.019
Frequency
0
50
100
150
200
250
−0.4
−0.2
0.0
0.2
0.4
Null distribution

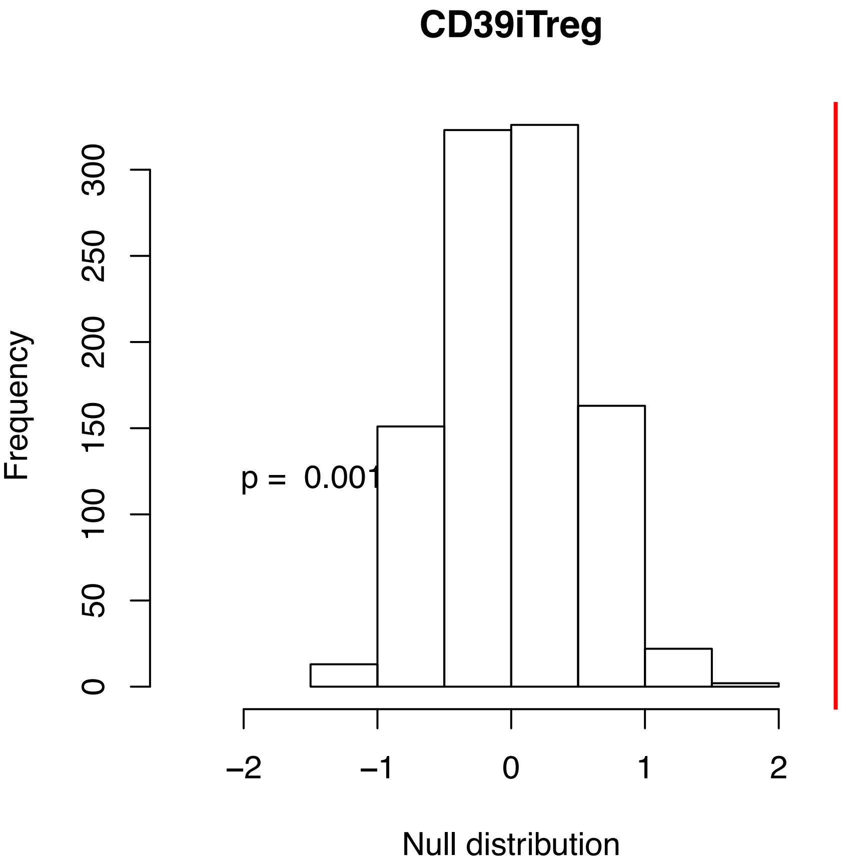
CD39iTreg
Frequency
0
50
100
150
200
250
300
p = 0.001
−2
−1
0
1
2
Null distribution

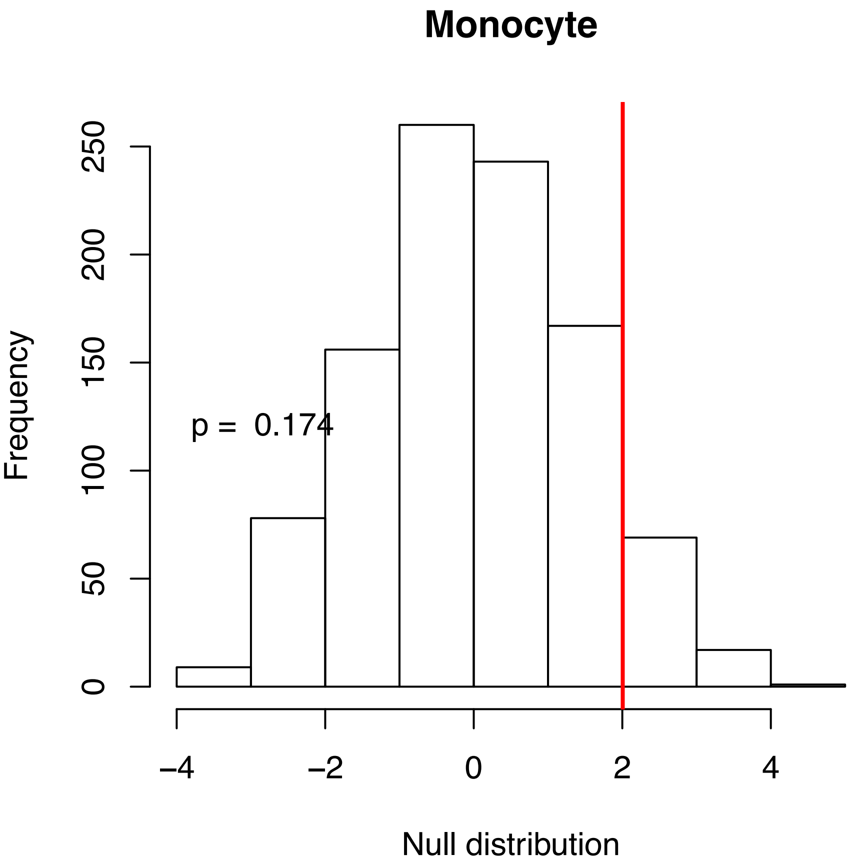

Monocyte
p = 0.174
Frequency
0
50
100
150
200
250
−4
−2
0
2
4
Null distribution

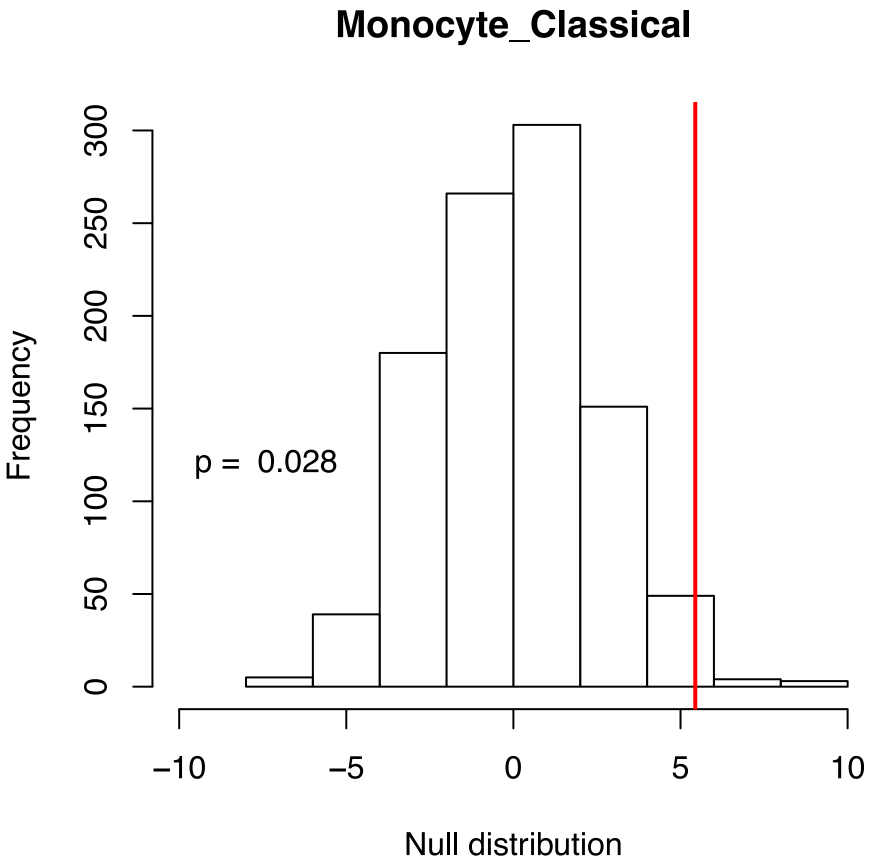
Monocyte_Classical
Frequency
0
50
100
150
200
250
300
p = 0.028
−10
−5
0
5
10
Null distribution

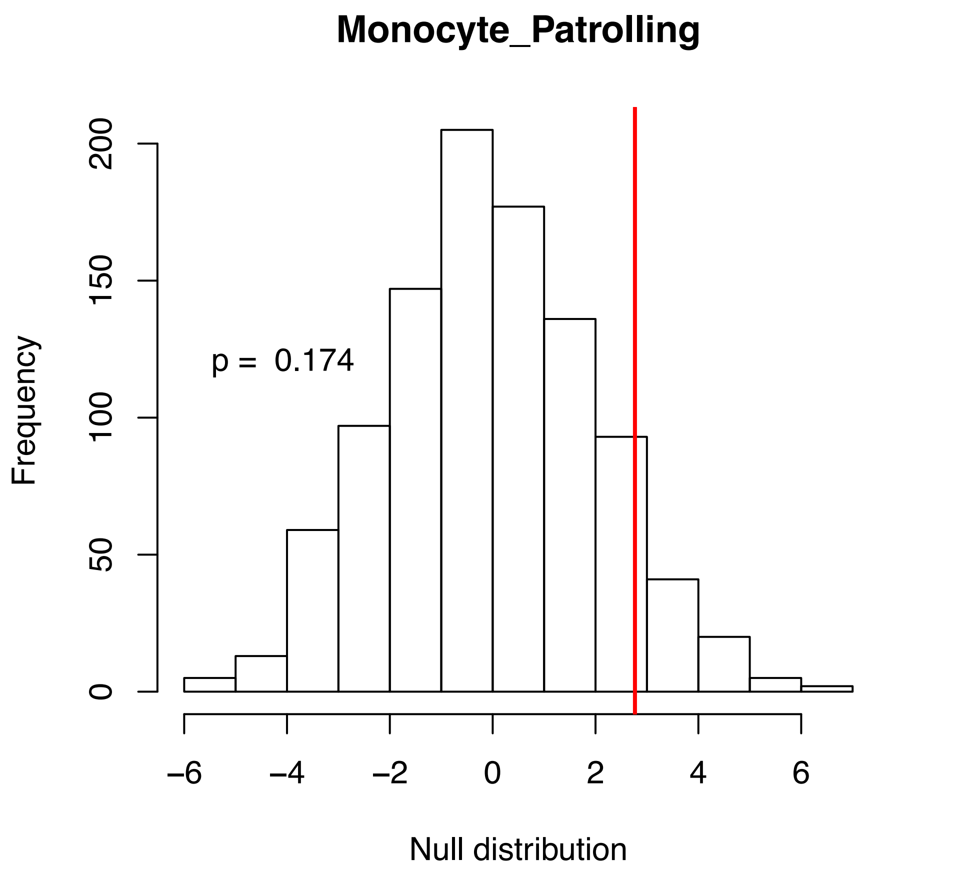

Monocyte_Patrolling
p = 0.174
Frequency
0
50
100
150
200
−6
−4
−2
0
2
4
6
Null distribution

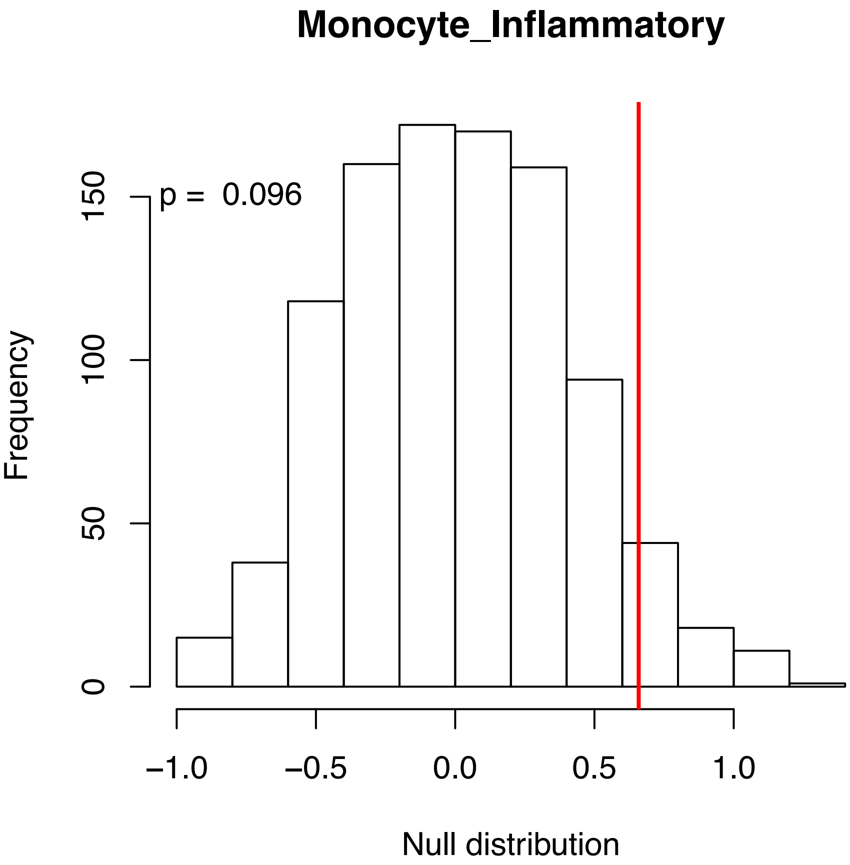

Monocyte_Inflammatory
p = 0.096
Frequency
0
50
100
150
−1.0
−0.5
0.0
0.5
1.0
Null distribution

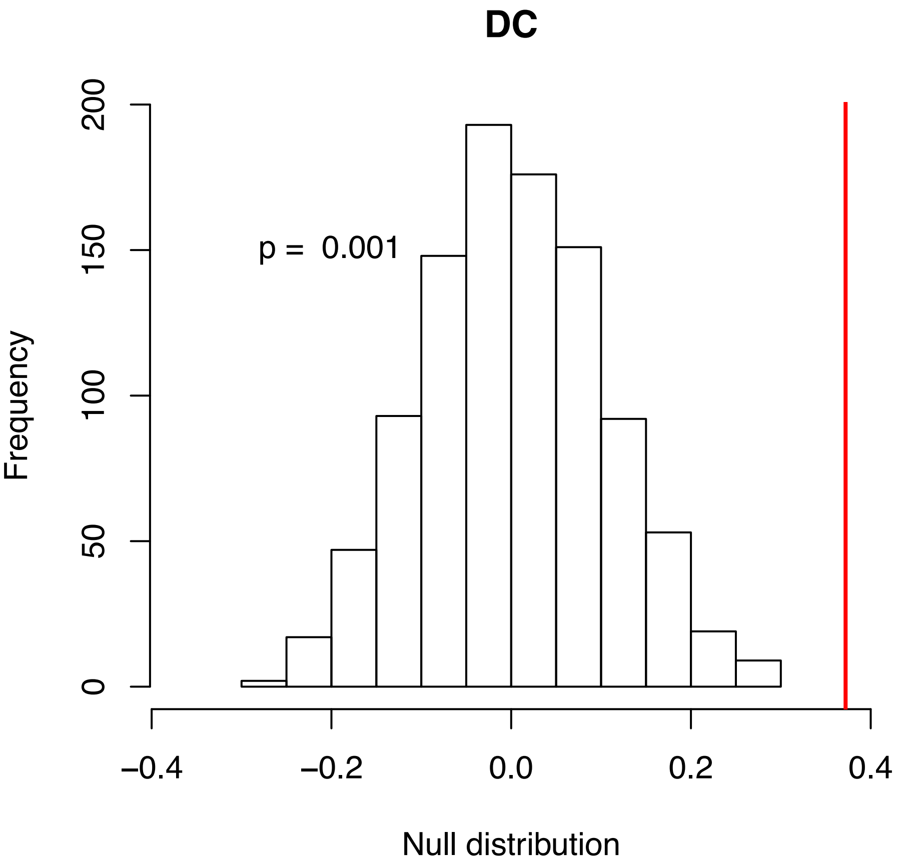
DC
p = 0.001
Frequency
200
150
100
50
0
-0.4
-0.2
0.0
0.2
0.4
Null distribution

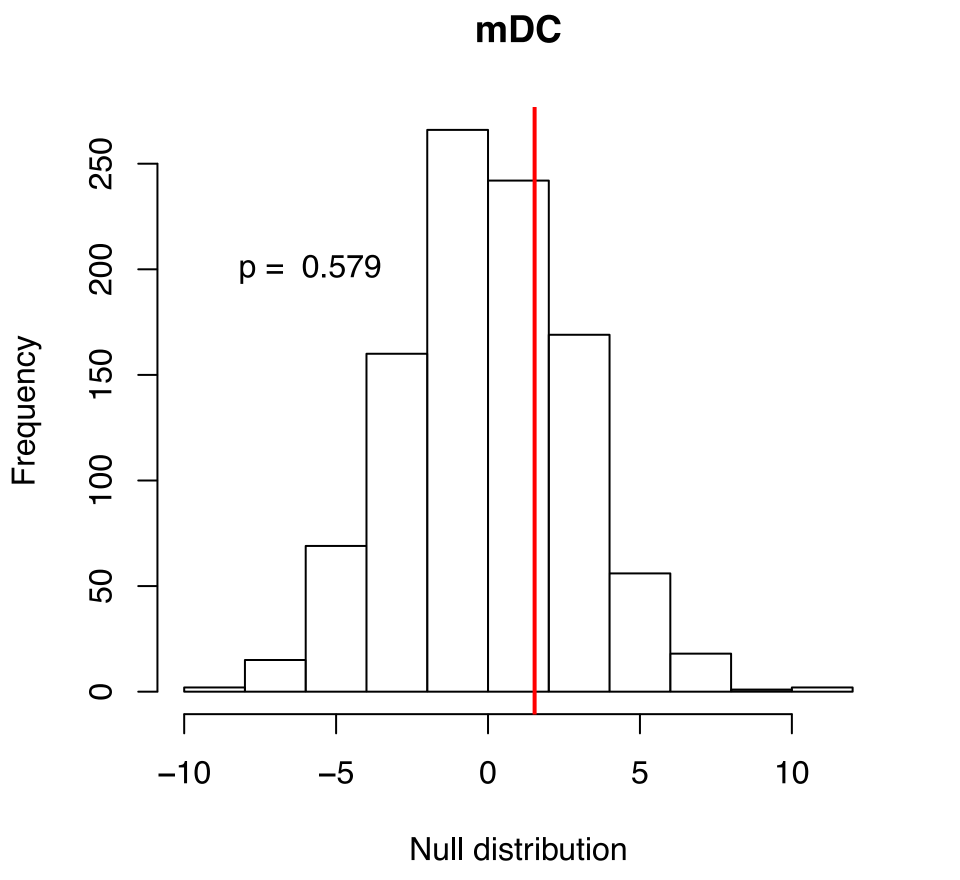

mDC
p = 0.579
Frequency
0
50
100
150
200
250
−10
−5
0
5
10
Null distribution

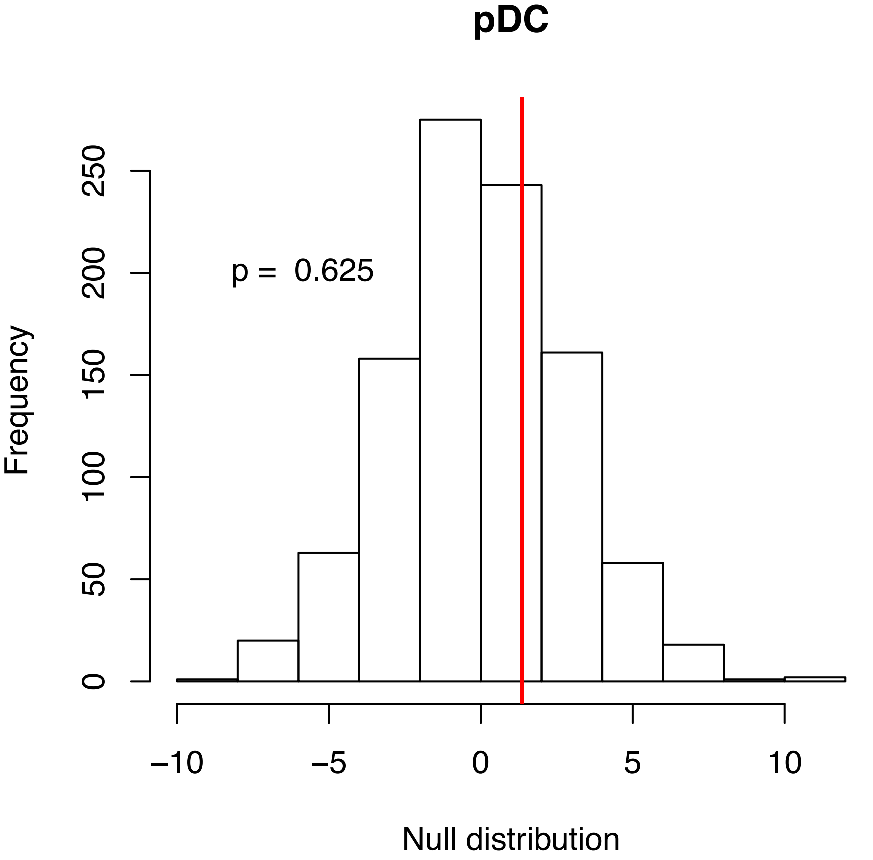

pDC
p = 0.625
Frequency
0
50
100
150
200
250
−10
−5
0
5
10
Null distribution

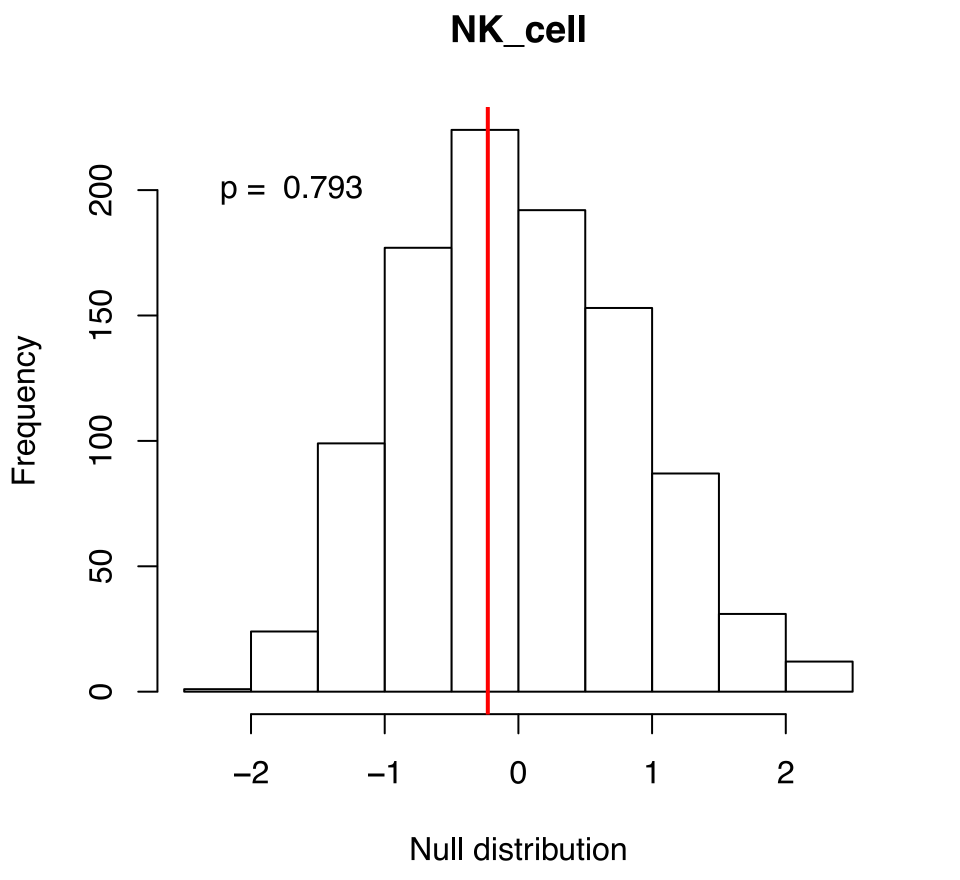
NK_cell
p = 0.793
Frequency
0
50
100
150
200
−2
−1
0
1
2
Null distribution

SUPPLEMENTARY FIGURES

Characterization of biological variation of peripheral blood immune cytome in an Indian cohort

Parna Kanodia, Gurvinder Kaur, Poonam Coshic, Kabita Chatterjee, Teresa Neeman, Anna George, Satyajit Rath, Vineeta Bal, Savit B Prabhu.

Supplementary Figure 11

Distance between immune subsets in unrelated pairs and sibling pairs. For the subsets indicated, sibling pairs and unrelated pairs did not differ in their immunological distance. Each subset is described as % of parent gate as defined in Supplementary Table S1. P-values for all subsets given in Supplementary Table S11.

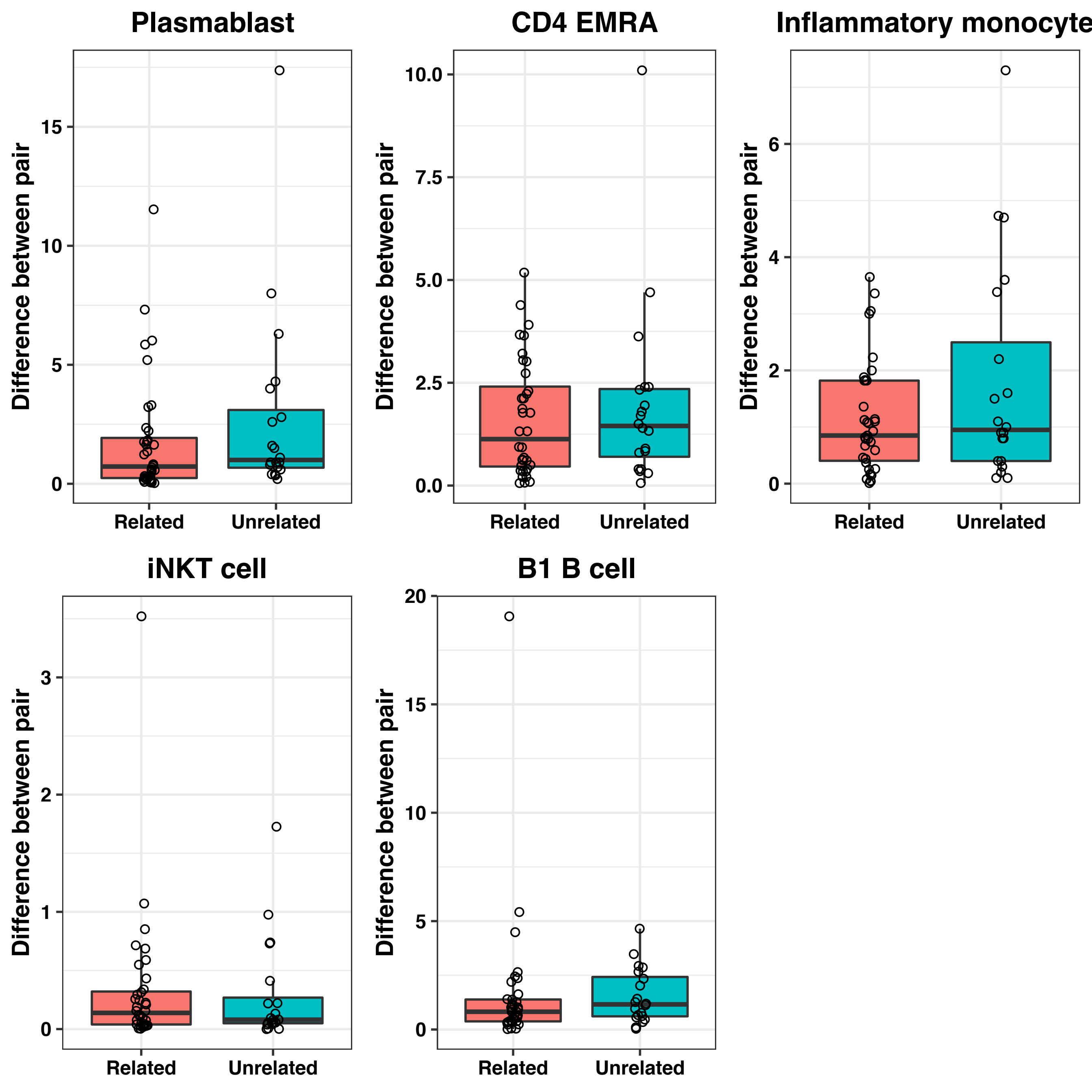
Plasmablast
CD4 EMRA
Inflammatory monocyte
iNKT cell
B1 B cell
Difference between pair
Related
Unrelated
0
5
10
15
0.0
2.5
5.0
7.5
10.0
0
2
4
6
0
1
2
3
0
5
10
15
20
